# Revision of the World Species of *Megaphragma* Timberlake (Hymenoptera: Trichogrammatidae) †

**DOI:** 10.3390/insects13060561

**Published:** 2022-06-20

**Authors:** Andrew Polaszek, Lucian Fusu, Gennaro Viggiani, Andie Hall, Paul Hanson, Alexey A. Polilov

**Affiliations:** 1 Department of Life Sciences, Natural History Museum, Cromwell Road, London SW7 5BD, UK; a.polaszek@nhm.ac.uk; 2 Research Group in Invertebrate Diversity and Phylogenetics, Faculty of Biology, Alexandru Ioan Cuza University, Bd. Carol I, Nr. 11, 700506 Iasi, Romania; 3 Laboratorio di Lotta biologica, Dipartimento di Agraria, Università degli Studi di Napoli “Federico II”, Via Università 100, 80055 Portici, NA, Italy; genviggi@unina.it; 4 Core Research Laboratories, Natural History Museum, Cromwell Road, London SW7 5BD, UK; a.hall@nhm.ac.uk; 5 Escuela de Biologίa, Universidad de Costa Rica, San Pedro de Montes de Oca, San Jose 11501-2060, Costa Rica; phanson91@gmail.com; 6 Department of Entomology, Faculty of Biology, Lomonosov Moscow State University, 119234 Moscow, Russia; polilov@gmail.com

**Keywords:** species delimitation, egg parasitoid, Chalcidoidea, integrative taxonomy, molecular phylogeny, DNA barcoding, mini-barcode

## Abstract

**Simple Summary:**

Parasitoid wasps of the genus *Megaphragma* are some of the smallest known insects, being as small as some unicellular protozoans. Their life history is not known in great detail, but all species with known biology are parasitoids of thrips eggs (Thysanoptera) and as such, they are potential biological control agents of these pests. At the current state of knowledge of the genus, it is impossible to identify with confidence most of the *Megaphragma* species (original descriptions lack essential details or illustrations; molecular markers are available for very few species; many species are still undescribed while others were described multiple times). We provide the first revision of the genus that includes the formal descriptions and naming of 22 species and a key to all 32 valid species.

**Abstract:**

*Megaphragma* species are important models for basic organismal research, and many are potential biological control agents. We present the first extensive revision of species of the genus *Megaphragma* based on morphological and molecular data. Our revision includes all previously described species, 6 of which are synonymized, and 22 of which are described here as new. We also provide the first key to all species of the genus and reconstruct their phylogeny based on 28S and CO1 molecular markers. The following species are synonymized with *M. longiciliatum* Subba Rao: *M. aligarhensis* Yousuf and Shafee **syn. nov.**; *M. amalphitanum* Viggiani **syn. nov.**; *M. decochaetum* Lin **syn. nov.**; *M. magniclava* Yousuf and Shafee **syn. nov.**; *M. shimalianum* Hayat **syn. nov.** *M. anomalifuniculi* Yuan and Lou **syn. nov.** is synonymized with *M. polychaetum* Lin. The following species are described as new: *M. antecessor* Polaszek and Fusu **sp. nov.**; *M. breviclavum* Polaszek and Fusu **sp. nov.**; *M. chienleei* Polaszek and Fusu **sp. nov.**; *M. cockerilli* Polaszek and Fusu **sp. nov.**; *M. digitatum* Polaszek and Fusu **sp. nov.**; *M. fanenitrakely* Polaszek and Fusu **sp. nov.**; *M. funiculatum* Fusu, Polaszek, and Viggiani **sp. nov.**; *M. giraulti* Viggiani, Fusu, and Polaszek **sp. nov.**; *M. hansoni* Polaszek, Fusu, and Viggiani **sp. nov.**; *M. kinuthiae* Polaszek, Fusu, and Viggiani **sp. nov.**; *M. liui* Polaszek and Fusu **sp. nov.**; *M. momookherjeeae* Polaszek and Fusu **sp. nov.**; *M. nowickii* Polaszek, Fusu, and Viggiani **sp. nov.**; *M. noyesi* Polaszek and Fusu **sp. nov.**; *M. pintoi* Viggiani **sp. nov.**; *M. polilovi* Polaszek, Fusu, and Viggiani **sp. nov.**; *M. rivelloi* Viggiani **sp. nov.**; *M. tamoi* Polaszek, Fusu, and Viggiani **sp. nov.**; *M. tridens* Fusu, and Polaszek **sp. nov.**; *M. uniclavum* Polaszek and Fusu **sp. nov.**; *M. vanlentereni* Polaszek and Fusu **sp. nov.**; *M. viggianii* Fusu, Polaszek, and Polilov **sp. nov**.

## 1. Introduction

Trichogrammatidae (Hymenoptera: Chalcidoidea) is a family of egg parasitoids that consists of approximately 100 genera and 1000 species [1,2,3]. The genus *Megaphragma* (tribe Oligositini) currently contains 15 species, all of which are egg parasitoids of Thysanoptera [1]. It includes some of the smallest insects: most species of this genus have a body length of only 0.16–0.3 mm, which is about the same size as a larger unicellular organism such as *Paramecium* [4]. Huber and Noyes [5] provided a review of the body size limit in insects with only three genera of Mymaridae having species smaller than the smallest *Megaphragma*. The genus has been recorded on all continents except Antarctica; though most of the species are confined to the tropics and warmer temperate regions. According to the available data, all species of this genus are egg parasitoids of thrips, but the biology of most species remains unknown. The type of the genus, *M. mymaripenne* Timberlake, was examined by Viggiani [6], who gave details on several previously unused features, in particular on the sculpture present on tergites of the metasoma. The same author described several new species of *Megaphragma* [7,8]. Lin [9] described five new species from China, and Hayat [10] revised the Indian species. The *Megaphragma* of Argentina were studied by Viggiani et al. [11]. Detailed biological data are available for *M. mymaripenne*, *M. polilovi* **sp. nov.** (under the name *M. mymaripenne*), and *M. longiciliatum* Subba Rao (under the name *M. amalphitanum* Viggiani) [12,13,14].

Due to their extremely small body size, *Megaphragma* species have become model organisms for studying the miniaturization of insects [15,16] and solving neurobiological problems [17]. The general anatomy and anatomical features associated with miniaturization have been described [18]; the structure of the eye [19], antenna [20,21], and leg structures used for grooming [22], and peculiar features of the genome [23,24,25] have been studied. Anucleate neurons have been found in three species of *Megaphragma* [18,26] and the unique phenomenon of lysis of the bodies and nuclei of cells at the pupal stage of development has been described [4]. Analysis of the connectome of *Megaphragma* [27,28] and reconstruction of sensory organs at the cellular and subcellular level [28] are currently underway.

In addition to their value for basic research, *Megaphragma* species are potential biological agents for the control of thrips, many of which are important agricultural pests [29,30].

Most *Megaphragma* species descriptions are very brief, and genetic markers are available only for one named species [23,25,31] and three unnamed species [32,33]. Identification keys to species are available only for a few regions and include only selected species. Many specimens cannot therefore be identified. Thus, the lack of a revision makes it extremely difficult to work with these wasps, which are important for basic and applied research.

## 2. Materials and Methods

### 2.1. Specimens and Depository Abbreviations

Specimens on slides, mostly type material, were received or deposited in the following institutions: Alexandru Ioan Cuza University of Iaşi, Romania, Lucian Fusu collection (**AICF**); Aligarh Muslim University, Aligarh, India (**AMU**: Mohammad Hayat); Australian National Insect Collection, Canberra, Australia (**ANIC**: Nicole Fisher); Canadian National Collection of Insects, Ottawa, Canada (**CNCI**: John Huber); CIRAD/UMR CBGP, Montferrier-sur-Lez, France (**CIRAD**: Gerard Delvare); Università di Napoli “Federico II”, Dipartimento di Agraria, Collezione di Entomologia, Portici, Italia (**DACE**: Gennaro Viggiani); Department of Zoology, Plant Protection College, Fujian Agricultural and Forestry University Fuzhou, Fujian, China (**FAFU**: Naiquan Lin); International Institute of Tropical Agriculture, Cotonou, Benin (**IITA**: Georg Goergen); Museo de Zoología, Universidad de Costa Rica (**MZUCR**). Natural History Museum of Oman (**NHMO**); Natural History Museum, London, UK (**NHMUK**: Natalie Dale-Skey); Musée Royal de l’Afrique Centrale, Tervuren, Belgium (**MRAC**: Eliane de Coninck); Plant and Food Research New Zealand (formerly **DSIR**: Jocelyn Berry); University of California, Riverside, USA (**UCRC**: Serguei Triapitsyn). Additional material was received for identification from several institutions, and a number of recent collections by the authors and Dr John Noyes (NHMUK) contributed substantial material to this revision.

### 2.2. Morphology

All material was examined on microscope slides for morphological characters using an Olympus BX63 microscope with Nomarski differential interference contrast (DIC) with 40× and 100× objectives. Since the lysis buffer used for DNA extraction (see below) contains proteinase K, there is no need to clear the body with KOH as usually performed before slide mounting. Instead, specimens were extracted from the lysis buffer with an adjustable volume pipette (0.5 to 10 µL) set at 1–2 µL to reduce liquid loss and transferred to distilled water to remove unwanted reagents. Afterward, they were dehydrated using a series of ethanol solutions of increasing concentration and cleared in clove oil as described by Noyes [34]. Afterward, some specimens were mounted laterally in Canada balsam while others were dissected and wings, antennae, head, and body were mounted separately under different coverslips following [34]. However, mounting the abdomen dorsal side up has the disadvantage of making the setae on the sides of the tergites very difficult to see. Where possible, the setae on the disc of the fore wing were counted on the upper and lower surfaces. Body colour was observed on both card-mounted specimens and on slide-mounted specimens in which the generally unremarkable body pigmentation remains preserved.

Selected specimens were dried using a critical point drier and examined with an electron microscope as described in Polilov [26].

**Figure 1:**
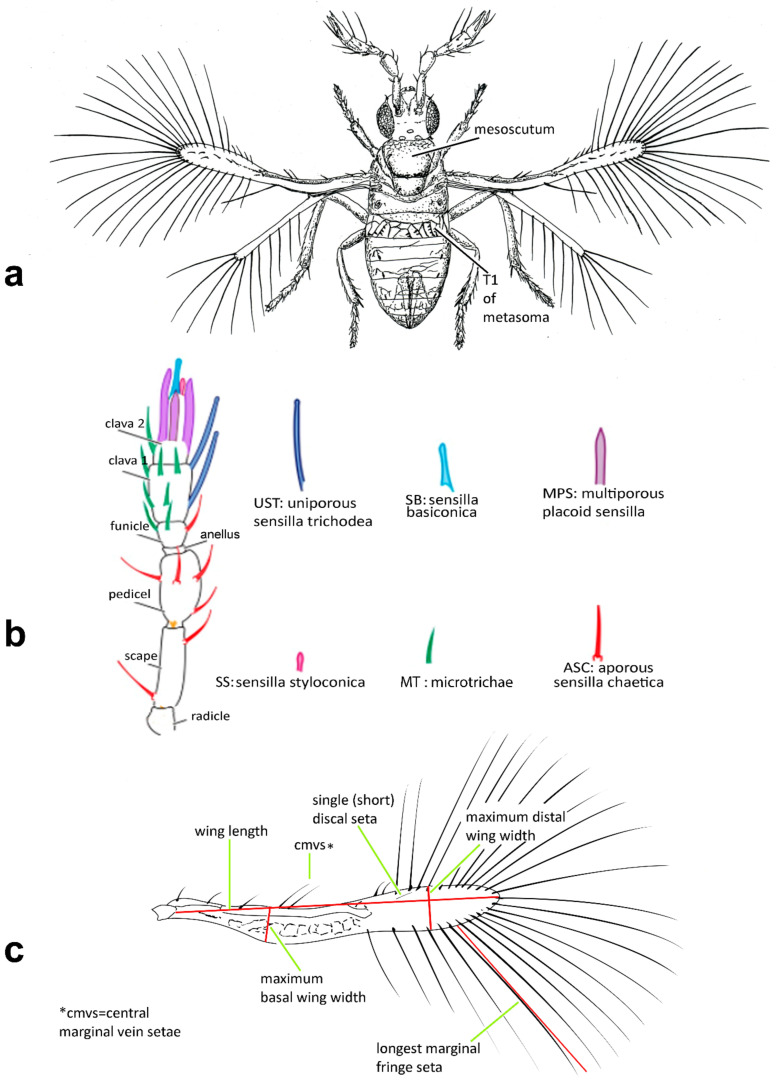
Line drawings of *Megaphragma* species: (**a**) *M. mymaripenne*, female habitus; (**b**) *Megaphragma* sp., stylized antenna; (**c**) *Megaphragma* sp. *ghesquierei*-group, fore wing.

Morphological terminology (see Figure 1) broadly follows Pinto [2], while terminology of the antennal sensilla follows Diakova et al. [20], albeit with abbreviations that follow the descriptions; e.g., “**UST**” for “uniporous sensilla trichodea” instead of Diakova et al. [20], who use “TS-UP”.

The following abbreviations are used for morphological terms (see Figure 1b): **ASC** = aporous sensilla chaetica; **C1** = 1st (basal) clavomere; **C2** = 2nd (central or apical) clavomere; **C3** = 3rd (apical) clavomere; **MPS** = multiporous placoid sensilla; **MT** = microtrichia (referred to as “aporous sensilla trichodea, type 1” by Diakova et al. [20]); **SB** = sensilla basiconica; **SS** = sensilla styloconica; **T1, T2** etc. = metasomal tergite 1, 2 etc.; **UST** = uniporous sensilla trichodea.

In species with a single discal fore wing seta, its length is important: “short” = shorter than or equal to the distance between the 2 proximal wing fringe setae (i.e., those closest to the seta); long = longer than the distance between the 3 proximal wing fringe setae (see Figure 1c).

A peculiar type of metafemoral spine with a unique shape, structure, and position is present in all species of the *ghesquierei*-group. Probably non-homologous metafemoral spines are present in other species groups.

### 2.3. Molecular Methods

DNA was extracted using a Qiagen DNeasy Blood and Tissue Kit with modifications as described in Cruaud et al. [35]; specimens were lysed whole for 6–8 h without grinding, then frozen at −80 °C overnight and thawed at room temperature before addition of buffer AL. After about the first 100 extractions the freezing stage was omitted as it appeared not to increase DNA yield significantly.

The primer pair D23F (5′-GAGAGTTCAAGAGTACGTG-3′) [36] and 28Sb also known as D3B (5′-TCGGAAGGAACCAGCTACTA-3′) [37,38] was used to amplify an approximately 850 bp fragment from the 5′ end of the nuclear ribosomal 28S gene spanning the D2–D3 region. In the instances where there was no detectable PCR product, we performed a second PCR using 1 µL of the primary PCR product and the semi-nested primer pair D23F combined with the newly designed reverse primer 28Sbsn (5′-GATGGTTCGATTAGTCTTTCG-3′), which amplified an approximately 800 bp fragment of the 28S rDNA.

The CO1 gene was amplified using the standard primer pair LCO1480 and HCO2198 [39], which amplifies the DNA barcode region for animals [40,41]. In case of failed reactions, we used a pair of internal primers from Fusu and Polaszek [42] that amplify the standard barcode region from two overlapping fragments (mini-barcodes, [43]): a modified LCO1490, named LCO1490M (5′-CAACAAATCATAAAGATATTGG-3′), pairs with MChaR1 (5′-CCYGTTCCAAYAAATATTCT-3′), and MChaF1 (5′-CCTCGAATAAATAATATAAGATT-3′) pairs with HCO2198.

The PCR conditions were as described in Fusu and Polaszek [42] except the standard barcode region was amplified at 42 °C.

All PCR products were checked by gel electrophoresis in 1% agarose gels, cleaned using AxyPrep PCR clean-up beads as per manufacturer’s instructions, then sequenced bi-directionally using BigDye terminator reaction mix v.3.1 in a 3730xl DNA analyser (Applied Biosystems) at the NHMUK sequencing facility.

The forward and reverse sequences were assembled and corrected using the Staden Package v.1.7.0 [44]. The resulting sequences were aligned in Mega v.7.0.14 [45] with the Clustal W program [46] for the CO1 gene; the 28S gene was aligned with the MAFFT web server [47] using the E-INS-i algorithm, a gap opening penalty of 2, leave gappy regions option activated, and UPGMA as a tree-building method for the guide tree. The CO1 sequences were also translated to amino acids to detect eventual stop codons that indicate NUMTs. The two alignments were first used in single-gene phylogenetic analyses in RAxML-NG v.1.0.0 [48] to detect eventual long branches and misplaced sequences (that might indicate pseudogenes or contaminants) that are to be checked/removed prior to the concatenation of the two datasets. A phylogenetic analysis of the concatenated but unpartitioned dataset using a simple substitution model (K2P) was also conducted in RAxML-NG since over-parameterization of the substitution and partition models might be a problem in a maximum likelihood framework [49], especially when using a comparatively small alignment. For the partitioned analyses, data blocks were delimited in Mesquite v.3.10 [50], CO1 being divided by codon position and 28S was treated as one block. The best partitioning scheme and substitution models were selected using PartitionFinder2 v.2.1.1 [51], with branch lengths proportionally linked and the search option set to all.

Partitioned analyses were run in RAxML-NG [48], which is maximum likelihood (ML) based, and MrBayes v.3.2.7 [52], which is based on Bayesian inference (BI) with the following substitution models as indicated by PartitionFinder2: GTR+G for 28S, HKY+I+G, TIM+I+G, and TIM+G for the 1st, 2nd, and 3rd codon positions of CO1, respectively. For MrBayes, we substituted TIM with GTR, since the former model is not available in this program. In MrBayes two parallel analyses, each with four chains, were run for 10^7^ MCMC generations, with trees and lnLs sampled every 100 generations; all estimated parameters were unlinked among partitions except for branch lengths; convergence of all parameters and estimated sample size values (ESS) above 200 were assessed by examining the trace files in Tracer v1.7.1 [53]. Support for the maximum likelihood analysis was estimated with rapid bootstrapping (number of replicates determined by the autoMRE criterion [54]). Bootstrap percentages (BP) over 85% were considered as strong support and BP smaller than 65% as weak. Posterior probabilities (PP) over 0.95 were considered as strong support and those below 0.90 as weak. The trees were imported and modified in FigTree v1.4.4 [55] and Adobe Illustrator.

All sequences were uploaded to GenBank (accession numbers ON555486–ON555643 for 28S and ON557406–ON557518 for CO1). Since a part of the DNA extractions did not yield PCR products, the presence of a DNA code after the label data of a specimen does not necessarily mean that it has an associated DNA sequence. A complete list of specimens with associated DNA sequences and their repository is provided in Appendix A.

### 2.4. New Species Left Undescribed

**Figure 2:**
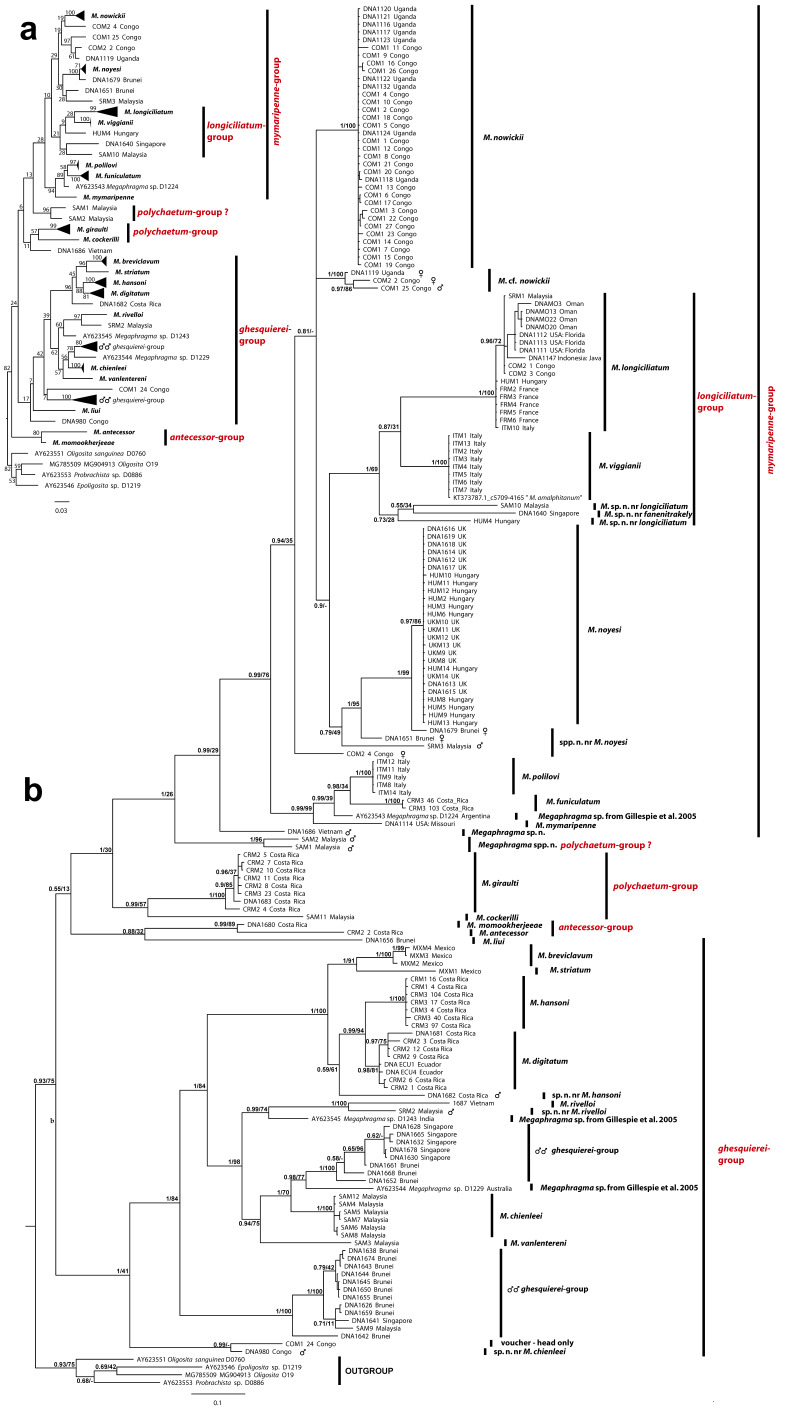
Combined CO1 and 28S sequences analysis of 174 *Megaphragma* specimens (170 from this study, 4 from GenBank) with species delineated and species-groups indicated: (**a**) unpartitioned ML analysis (bootstrap values indicated at nodes); (**b**) partitioned BI analysis (both posterior probabilities and bootstrap values indicated at nodes).

We have identified several species that are clearly new based either on their DNA sequences or morphology (or both), but are not described herein for one or more of the following reasons:

- Species known from males only. Within (e.g.) the *ghesquierei*-group, several new species have been identified (at least 7 or 8—see Figure 2), which are known only from males. Since in most cases females are essential for species recognition (e.g., antennal structure, ovipositor length), we have refrained from describing these species here.
- Incomplete specimens. In several instances, new species are indicated by both morphology and DNA sequences, but a crucial morphological character is missing, most often the antennae. These specimens and their sequences have been curated pending the discovery of fresh, complete specimens.
- Poorly-mounted specimens. In a few cases, slide-mounted specimens not represented by DNA sequences appear to be very likely new species. In many cases, the material is simply not in good enough condition for the designation of a holotype to represent the species.

Clearly, there is overlap and gradation between the above categories, and we have used our discretion when deciding whether or not to describe specimens. In all cases, information as to our opinion of species status is included on the specimens.

### 2.5. A Note on Figures Supplementing the Descriptions

While all new species are fully described, in many cases there are aspects of the morphology that differ so little between species that images of these structures would be superfluous. In these cases, “cf Figure” is used, where the reader is referred to a figure that to all intents and purposes can serve to illustrate the species while actually depicting a different one. This is especially true for many species of the *ghesquierei*-group, where several species are morphologically indistinguishable, and to a lesser extent for *M. mymaripenne*, *M. noyesi* **sp. nov.**, and *M. polilovi* **sp. nov.** in the *mymaripenne*-group. In every case of extreme morphological similarity, robust molecular data are available to support separate species status. In addition, where possible, illustrations were made from holotypes. Under each photograph, we mention whether it is that of a holotype, neotype, or paratype.

## 3. Results

### 3.1. Phylogenetic Analyses

We obtained DNA sequences for a total of 170 *Megaphragma* specimens (158 sequences for 28S and 113 sequences for CO1) (Appendix A). The 28S alignment was 1068 bp in length, while the CO1 alignment was 652 bp, though only shorter sequences (DNA mini-barcodes) were obtained for some species/specimens.

The phylogenetic trees from the single-locus analyses are in general agreement though, for example, the position of *M. antecessor* **sp. nov.** differs drastically between the two, while some species are present in one data matrix but not the other (Supplementary Figures S2 and S3). They are also in general agreement regarding the clustering of specimens into putative species: all species that are distinct based on CO1 are also distinct based on 28S. An exception is two specimens of *M. digitatum* **sp. nov.** that are very divergent on CO1, but almost identical on 28S. Both the ML and BI trees from the combined and partitioned dataset show similar topologies, with minor differences; hence, in Figure 2b on the BI tree, both posterior probabilities and bootstrap support were plotted at the nodes. The species that were included in the analysis are split into two major groups: one consisting of mostly species of the *mymaripenne*-group (species groups are discussed below), the other of species of the *ghesquierei*-group. Both have high posterior probabilities (0.99 and 1, respectively) but low bootstrap support, indicating strong support based on a low number of characters. Two other small groups are formed by two species each in the *antecessor*- and *polychaetum*-groups. The *antecessor*-group is sister to *M. liui* **sp. nov.** in both of these trees, while two unnamed species of the *polychaetum*-group (SAM1 and SAM2) are basal to the *mymaripenne*-group instead of clustering with *M. giraulti* **sp. nov.** and *M. cockerilli* **sp. nov.** (the other two species of the group). The two species of the *longiciliatum*-group, though forming a monophyletic group with a posterior probability of 1, and a medium bootstrap support of 69%, render the *mymaripenne*-group paraphyletic.

The two trees based on the combined analysis of both genes have a major difference from the unpartitioned analysis that used a simple substitution model and not the best fit model (Figure 2a); in this latter tree, *M. liui* is recovered in a basal position in the *ghesquierei*-group, where it belongs based on its morphology. Another major difference between the partitioned analyses and the unpartitioned one is the position of the *antecessor*-group that is not sister to the *ghesquierei*-group in the first analyses (Figure 2b) as would be expected by its morphology, while it is retrieved as basal to all other *Megaphragma* in the second tree (Figure 2a).

Even very short DNA sequences are sufficient to place a specimen, though in some cases this is also the explanation for the unstable and likely erroneous position in the phylogenies for some species. For example, HUM9 is correctly placed in the cluster with other *M. noyesi* based on a 296 bp CO1 sequence and the same is true for *M. momookherjeeae* **sp. nov.** retrieved as sister to *M. antecessor* based on a 394 bp CO1 sequence (the morphology of both species places them in the *antecessor*-group). *Megaphragma antecessor*, *M. liui,* and *M. momookherjeeae* that have their positions on the trees drastically altered depending on the analysis (partitioned versus unpartitioned) are represented by short sequences: 519 bp for 28S and 366 for CO1, 344 bp for 28S and 370 for CO1, and 394 bp for CO1, respectively.

### 3.2. Taxonomy

***Megaphragma* Timberlake, 1924**

*Megaphragma* Timberlake, 1924. *Proc. Haw. Entomol. Soc*. 5: 412–414. Type species: *Megaphragma mymaripenne* Timberlake, by original designation.
*Sethosiella* Kryger, 1932. *Bulletin de la Société Royale d’Egypte* 16: 38–39. Type species: *Sethosiella priesneri* Kryger, by original designation. Synonymy by Ghesquière 1939, p. 36.
*Paramegaphragma* Lin, 1992. *Entomotaxonomia* 14(2): 133–135, 138. Type species: *Paramegaphragma stenopterum* Lin, by original designation. Synonymy by Delvare 1993, p. 151.

*Diagnosis.* Female (Figure 1a). Body rather compact, extremely small, length 0.16–0.3 mm. Antenna (Figure 1b) inserted at mid level of the internal orbital line, with short radicle, scape usually elongate, pedicel, anellus, single funicle segment present or absent, clava one -, two -, or three-segmented. Antennal formula: 1 (scape), 1 (pedicel), (1) (anellus), 1 (funicle), 2 (clava); or 1,1,(1),1,1 or 1,1,(1),0,3. The antenna is counted as four- or five-segmented, since the anellus is not counted among the antennomeres. Claval segment 1 without multiporous placoid sensilla. Mandible with two small teeth. Maxillary palp very small and labial palp vestigial. Eye black unless otherwise stated. Mesosoma rather high, usually shorter than metasoma. Pronotum very short; mid lobe of mesoscutum not much longer than wide, either smooth or with polygonal or striate sculpture; one pair of adnotaular setae. Scutellum shorter than mid lobe of mesoscutum, with a pair of setae. Metanotum short; propodeum slightly longer than metanotum, or, in the middle, even longer, with a well-developed central area (disc) that may bear crenulae. Propodeal spiracle placed in an oval groove, and near the internal margin with two very small setae. Fore wing (Figure 1c) extremely narrow compared with other Trichogrammatidae genera, 5.3–10× as long as maximum discal width, with short submarginal vein; costal cell and parastigma not distinct; marginal vein very long, with one short seta at the base and with one or two setae centrally, which when paired may be of similar or very different lengths; stigmal vein very short with one or two short setae on the stigma; disc with one or a few setae in one or two rows or glabrous (when there is one seta it is located on the dorsal surface of the wing, when discal setae are more numerous they are located on both dorsal and ventral surfaces of the wing, cf Figure 22d,e). Hind wing without discal fringe on front margin. Legs robust, often with striate sculpture on coxae, also on femora and tibiae. T7 and T8, respectively, without spiracle and cercus.

*Male*: As female, but often with postanellar antennomeres shaped differently. Genitalia tubular, very simple and usually small.

*Relationship*. The closest relatives of *Megaphragma* appear to be *Prestwichia* Lubbock and *Sinepalpigramma* Viggiani and Pinto [56]. Unfortunately, sequences for neither of these genera were available for comparison. We have used an *Epoligosita* Girault, two *Oligosita* Walker, and a *Probrachista* Viggiani species as outgroups. These Oligositinae genera are close phylogenetically to *Megaphragma* according to a previous molecular study [33]. Species-group relationships are discussed below.

*Distribution*: Cosmopolitan.

*Hosts and biology*. The known species of *Megaphragma* are all egg parasitoids of Thysanoptera (Supplementary Figure S4) [57,58,59]. Biological data are available only for a few species, e.g., *M. mymaripenne, M. longiciliatum* (as *M. amalphitanum*) [12,13,14], and are given below where available. It is interesting that at the same locality there may be more than one species of *Megaphragma,* even in Europe. *Megaphragma viggianii* and *M. polilovi* were found in Italy at the same locality and on the same host, while in a single sample from near Barkás Lake in Hungary, there are three species (*M. longiciliatum*, *M. noyesi,* and the undescribed species represented by the specimen HUM4, close to *M. longiciliatum* but distinct genetically).

**Species groups in *Megaphragma***

On the basis of present knowledge, the following species group are proposed in *Megaphragma*:

*M. mymaripenne*-group: antenna with a single funicle segment that is longer than wide (this feature also shared by *polychaetum*- and *longiciliatum*- groups); **T1** with longitudinal and/or transverse cells, with some denticles laterally within the cells (Figures 17f, 18f and 20b); **T2**–**T4** each with a pair of short setae.

Included species: *M. funiculatum* Polaszek and Fusu **sp. nov.**, *M. mymaripenne* Timberlake, *M. nowickii* Polaszek, Fusu, and Viggiani **sp. nov.**, *M. noyesi* Polaszek and Fusu **sp. nov.**, *M. polilovi* Polaszek, Fusu, and Viggiani **sp. nov**.

*M. longiciliatum*-group: same as *mymaripenne*-group, but without cells on **T1**. According to the phylogenetic analysis, the group appears to be derived from within the *mymaripenne*-group having lost the denticulate cells on **T1**.

Included species: *M. longiciliatum* Subba Rao, *M. fanenitrakely* Polaszek and Fusu **sp. nov.**, *M. priesneri* Kryger, *M. viggianii* Polaszek, Fusu, and Polilov **sp. nov.** The species of the *macrostigmum*-group (*M. caribea* and *M. macrostigmum*) characterized by a four-segmented antenna, might be derived species within this group.

*M. polychaetum*-group: antenna with a long, cylindrical, funicle segment; spatulate sensilla at the end of each clava segment, and a robust terminal sensillum on **C2**; fore wing disc with more than seven setae, often arranged in two rows. Male antenna is particularly distinctive, with an elongate **C1**, short **C2** usually with very long sensilla.

Included species: *M. cockerilli* Polaszek and Fusu **sp. nov.**, *M. giraulti* **sp. nov.**, *M. polychaetum* Lin, *M. kinuthiae* Polaszek, Fusu, and Viggiani **sp. nov.** Our molecular analysis also includes two males of this group, representing two undescribed species (vouchers SAM1 and SAM2, NHMUK). They have the antennal structure characteristic for males of the group, but our analyses recover them basal to the *mymaripenne*- and *longiciliatum*- groups instead of clustering them with the other two species of the *polychaetum*-group.

*M. ghesquierei*-group: antenna without funicle segment and with clava three-segmented, because the funicle is fused with the clava along an oblique suture. Fore wing disc with one seta on the dorsal surface or no setae. Propodeum characteristically produced centrally, almost always with a row of crenulae. Metafemur with a robust spine close to the connection with the tibia. Because of the intergradation in the structure of the antenna between the *ghesquierei* and other groups, we do not currently consider *Paramegaphragma* as a valid genus for the species in the *ghesquierei*- plus *stenopterum*- groups. It is possible that future analyses, especially including multigene or reduced genome representation data, may lead to the reinstatement of *Paramegaphragma* Lin as a valid genus. The two species formerly assigned to *Paramegaphragma* by Lin [9], *M. stenopterum* and *M. macrostigmum*, are not closely related and clearly belong to different species-groups (*stenopterum*-group and *macrostigmum*-group, respectively), though on morphological grounds *stenopterum*-group is clearly related to *ghesquierei*-group or even integral part of it. This is another reason for not recognizing *Paramegaphragma*.

Included species: *M. breviclavum* **sp. nov.**, *M. chienleei* **sp. nov.**, *M. deflectum* Lin, *M. digitatum* **sp. nov.**, *M. ghesquierei* Ghesquière, *M. hansoni* **sp. nov.**, *M. liui* **sp. nov.**, *M. pintoi* Viggiani **sp. nov.**, *M. rivelloi* **sp. nov.**, *M. striatum* Viggiani, *M. tamoi* Polaszek, Fusu, and Viggiani **sp. nov.**, *M. tridens* Fusu and Polaszek **sp. nov.**, *M. vanlentereni* Polaszek and Fusu **sp. nov**.

*M. stenopterum*-group: same as *M. ghesquierei* but with clava two-segmented. The antennal structure is very suggestive of the *ghesquierei*-group, given the similarity between the apparent **C1** of the *stenopterum*-group and that of the *ghesquierei*-group; i.e., it is actually a funicle completely fused to the clava. In the *antecessor*-group, the funicle is distinct albeit transverse and anneliform. Pending further evidence, we consider the *stenopterum*-group as possibly nested within the *ghesquierei*-group. *Megaphragma macrostigmum* and *M. caribea* (*macrostigmum*-group) were considered by previous authors to belong in a group with *M. stenopterum* [60], and *M. macrostigmum* with *M. stenopterum* were both originally included by Lin [9] in his genus *Paramegaphragma*. However, the former two species lack any of the obvious apomorphies of the *ghesquierei*-group except for the apparently lost funicle. Members of the *macrostigmum*-group are otherwise similar in the structure of the fore wing and sculpture of the mesoscutum to the species in the *longiciliatum*-, *mymaripenne*-, and *polychaetum*- groups and are probably not related to the *ghesquierei*- and *stenopterum*- groups. Our molecular analysis did not include *M. stenopterum*, the only member of this species group.

Included species: *M. stenopterum* (Lin).

*M. antecessor*-group: antenna with a transverse funicle segment not much larger than the anellus, and clava one- or two-segmented. Metatibia with a characteristic row of setae (Figures 11d and 21j). The structure of the antenna seems intermediate between that characteristic of the *longiciliatum*- and *mymaripenne*- groups and that of the *ghesquierei*-group. In the latter species group, the antenna is apparently without a funicle, as the funicle is completely fused with the clava by an oblique suture and, hence, the clava appears three-segmented. Our phylogenetic analysis shows that *M. antecessor* and *M. momookherjeeae*, while resembling the *ghesquierei* species group in many features (including fore wing structure and the robust spine on metatibia), appear outside it, and basal to all remaining *Megaphragma* except members of *ghesquierei*-group (partitioned analyses) or the most basal species group of all *Megaphragma* (unpartitioned analysis).

Included species: *M. antecessor* Polaszek and Fusu **sp. nov.**, *M. momookherjeeae* Polaszek and Fusu **sp. nov.**, *M. uniclavum* Polaszek and Fusu **sp. nov**.

*M. macrostigmum*-group: as explained above, *M. macrostigmum* and *M. caribea*, while undoubtedly very closely related to each other, appear to have no connection with the *ghesquierei*-group (our molecular analysis did not include either of these two species). The antenna has the clava two-segmented and no funicle as in the *stenopterum*-group; the fore wing structure, however, is not similar to the *ghesquierei*-group but suggestive of the *longiciliatum*-group, especially *M. priesneri*; metafemur without the robust spine characteristic for the *antecessor*-, *ghesquierei-,* and *stenopterum*- groups.

Included species: *M. caribea* Delvare, *M. macrostigmum* (Lin).

**Previously described species**

**Figure 3:**
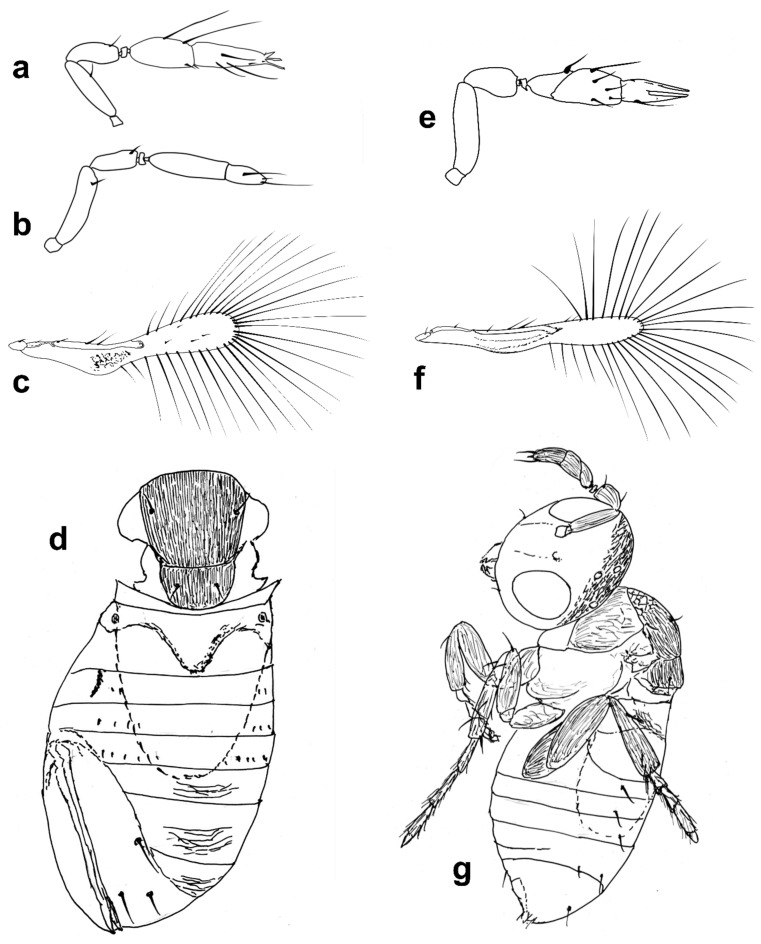
Line drawings of *Megaphragma* species: (**a**) *M. caribea*, female antenna (Paratype); (**b**) *M. caribea*, male antenna (Paratype); (**c**) *M. caribea*, female fore wing (Paratype); (**d**) *M. deflectum*, female body dorsal (Holotype); (**e**) *M. deflectum*, female antenna (Holotype); (**f**) *M. deflectum*, female fore wing (Holotype); (**g**) *M. ghesquierei*, male body lateral (Paratype).

***Megaphragma caribea* Delvare** (Figure 3a–c, Figure 12d–f and Figure 19a–c)

*Megaphragma caribea* Delvare, 1993. *Revue fr. Ent. (n.s.)* 15(4): 149–151.

*Diagnosis*. *Female*: Antenna four-segmented (excluding anellus), without funicle, clava two-segmented (Figure 3a and Figure 12d); **C1** with 16 **MT**, 2 long **UST**, 1 **SS**; **C2** with 4 **MPS**, 2 **MT**, 1 SB, 1 prominent apical **SS** (Figure 3a and Figure 12d).

Mid lobe of mesoscutum with large, but shallow polygonal cells (Figures 12e and 19c). Propodeum with central area short. Fore wing 5.5× as long as maximum discal width, marginal vein about twice length of submarginal vein, with two rather long setae (of equal length) present at midpoint of marginal vein (Figure 3c). Metasoma with lines of microspines evident on **T2**–**T6** (cf Figure 19a). Ovipositor 1.7× as long as mesotibia.

Body yellow, with the following slightly darkened: occiput, meso- and meta-coxae, apices of meso- and metafemora. Metasoma with pale brown transverse bands.

*Male*: Antenna with **C1** longer than in female (Figure 19b).

*Material examined*. Paratypes: **GUADELOUPE**: Vieux Habitants, 17.XI.1988, coll. J. Etienne, ex eggs *Selenothrips rubrocinctus* (Giard) on *Psidium guajava*. Slides n. 8002.4, 8002.6, 8002.9 (2♀, 1♂, NHMUK).

Non-types: **COLOMBIA**: Cartagena, i.2015, with *Heliothrips haemorrhoidalis* and *Selenothrips rubrocinctus* on *Terminalia catappa*, coll. A.A. Polilov (1♂, AICF).

Species-group placement: *M. macrostigmum*-group—possibly a subgroup of the *M. longiciliatum*-group.

*Distribution*: Colombia, Guadeloupe.

Host: *Selenothrips rubrocinctus* (Giard).

*DNA data*: no DNA sequences.

*Comments*: The species was described in detail by the author. *Megaphragma caribea* is clearly close to *M. macrostigmum* (Lin). At present, their discrimination is based on the absence of long **UST** on the basal clava (**C1**) of the antenna of the latter species (Figure 6a). Since the original description did not indicate whether the species-group name *caribea* is a noun or an adjective, following Art. 31.2.2. of ICZN, we treat it as a noun and do not make a gender agreement.

***Megaphragma deflectum* Lin** (Figure 3d–f)

*Megaphragma deflectum* Lin, 1992. *Entomotaxonomia* 14(2): 130–131.

*Diagnosis*. *Female*: Antenna (Figure 3e) without funicle, clava three-segmented, with **C1** and **C2** almost fused; **C1** with 1 **UST**; **C2** with 1 **UST**, and ≥4 **MT**; **C3** with ≥2 **MPS**, 1 **MT**, and 1 SB.

Mid lobe of mesoscutum (Figure 3d) with longitudinal striate sculpture extending to scutellum; propodeum (Figure 3d) with central area extended posteriorly, crenulae absent. Fore wing (Figure 3f) 9× as long as maximum width; longest fringe seta 5× as long as maximum discal width. Fore wing disc without setae. Marginal vein with two long subequal setae centrally. Stigmal vein not enlarged, with two sensilla apically. Middle tibia with one large spine basally; metafemur with spine. **T1** with elongate cells laterally, 2–3× as long as wide; **T2**–**T4** without setae laterally. Ovipositor 1.7× as long as mesotibia.

Body largely brown, the following paler: legs except coxae and metafemur. Antenna with pedicel pale; scape, **C1**–**C3** darker. Fore wing strongly infuscate basally; stigmal and marginal veins brown; marginal vein very dark centrally.

*Male*: As female but **C3** with fewer **MPS** and with ASC apically.

*Material examined*. Holotype ♀ (FAFU). **CHINA**: Wuyishan, Fujian, 19.x.1987, Wang Jiashe col.

Paratype: **CHINA**: Fuzhou, Fujian, 8.v.1987, N.Q. Lin col. (1♀, FAFU).

Non-type: **CHINA**: Wuyishan, Fujian, 10.x.1987, Wang Jiashe col. (1♂, FAFU).

*Species*-*group placement*: *M. ghesquierei*-group.

*Distribution*: China.

*DNA data*: no DNA sequences.

*Comments*: This species was correctly considered allied to *M. ghesquierei* Ghesquière mostly due to features of the antenna (Figure 3e), fore wing (Figure 3f), and other characteristics of the body, but *M. deflectum* can be distinguished easily from that species by the sculpture of the mid lobe of the mesoscutum and the central area of propodeum (Figure 3d). The male “allotype” (paratype) of *M. deflectum* is actually a male of *M. rivelloi* **sp. nov.** (see below).

**Figure 4:**
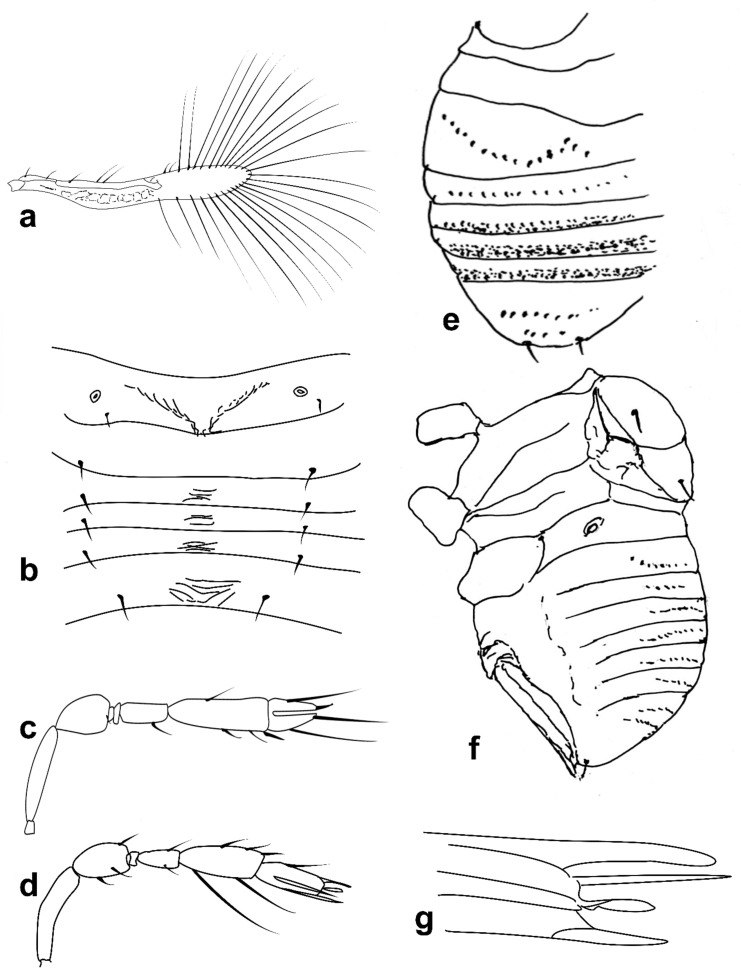
Line drawings of *Megaphragma* species: (**a**) *M. ghesquierei*, female fore wing (Holotype); (**b**) *M. ghesquierei*, male propodeum and tergites (Paratype); (**c**) *M. giraulti*, male antenna (Paratype); (**d**) *M. giraulti*, female antenna (Holotype); (**e**) *M. giraulti*, male metasoma (Paratype); (**f**) *M. giraulti*, female lateral meso- and metasoma (Holotype); (**g**) *M. giraulti*, female antenna, detail (Holotype).

***Megaphragma ghesquierei* Ghesquière** (Figure 3g and Figure 4a,b)

*Megaphragma ghesquierei* Ghesquière, 1939. *Rev. Zool. Bot. Afr.* 33(1): 36–38.

*Diagnosis*. *Female*: Antenna without funicle, clava three-segmented, **C1** distal margin transverse (cf Figure 3g). **C1** with ≥1 **MT**; **C2** with ≥2 **MT**, ≥2 **UST**; **C3** with ≥2 **MPS**, 1 **MT**, and 1 **SB**.

Mid lobe of mesoscutum anteriorly with reticulate sculpture, remainder with longitudinal striation continuing onto scutellum (cf Figure 3g). Propodeum with a large subtriangular central area. Fore wing 7× as long as maximum width (Figure 4a); the disc pointed distally, without setae. Metasoma with tergites with some short transverse striation centrally, and each with a pair of lateral setae (Figure 4b).

Body dark brown, with the following paler: frons and occiput, scutellum and propodeum, tarsi. Metasoma with tergites and sternites appearing as dark bands (in the slide-mounted types). Fore wing basally strongly infuscate with a dark marginal vein.

*Male*: Similar to female in all aspects of morphology except genitalia characters.

*Material examined*. Holotype ♀ (MRAC). **D. R. CONGO**: Rutshuru, i.1938, ex eggs of *Panchaetothrips noxius* Priesner on *Coffea arabica*.

Paratypes: **D. R. CONGO**: 1♂, on slide with holotype; 3♀ on one slide, with data as holotype except “Neotopotype” in Ghesquière’s writing (MRAC).

Non-type: 1♂, labeled type in the Nowicki collection, no other data (DACE).

*Species-group placement*: *M. ghesquierei*-group.

*Distribution*: D. R. Congo.

*Host*: *Panchaetothrips noxius* Priesner

*DNA data*: no DNA sequences.

*Comments*: The species is rather easily recognizable by the combination of features of the antenna, mid lobe of mesoscutum, propodeum, fore wing, and metasomal tergites.

The species was intended to be described by Nowicki, but was published by Ghesquière [61] (p. 36) because Nowicki’s manuscript on several African Trichogrammatidae never reached the journal Revue de zoologie et de botanique Africaines in Tervuren where Ghesquière was working. Ghesquière [61] (p.37) gives the date of collection as “XII.1937”, but, as given above, the holotype is labeled: “I.1938”.

**Figure 5:**
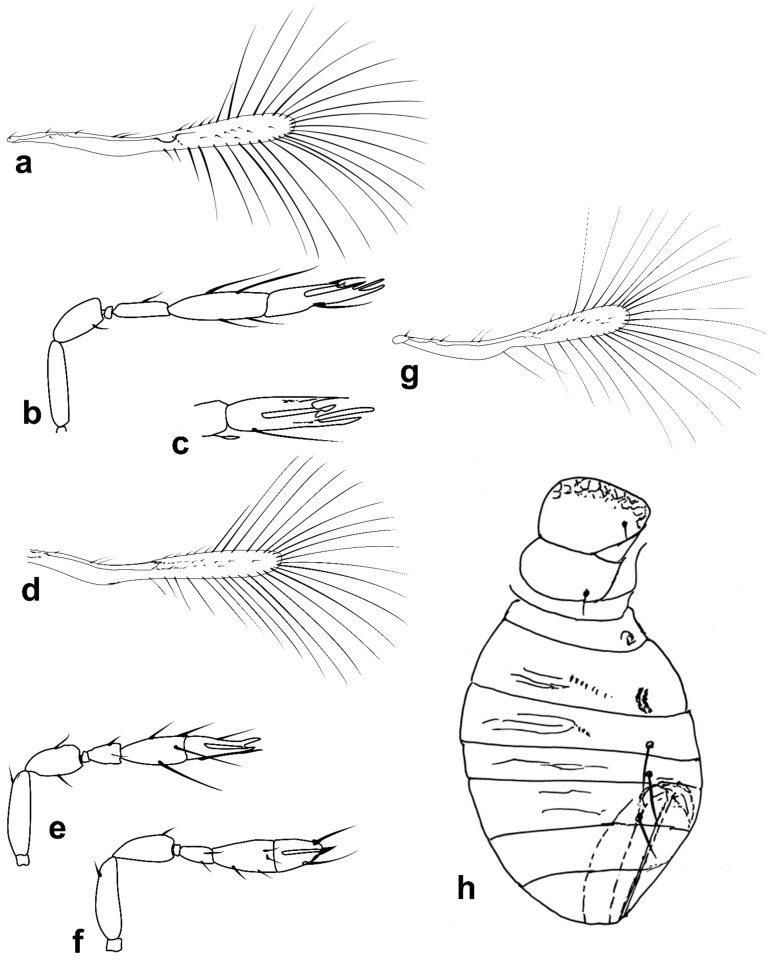
Line drawings of *Megaphragma* species: (**a**) *M. giraulti*, female fore wing (Holotype); (**b**) *M. kinuthiae*, female antenna; (**c**) *M. kinuthiae*, female antenna (detail); (**d**) *M. kinuthiae*, female fore wing; (**e**) *M. longiciliatum*, female antenna; (**f**) *M. longiciliatum*, male antenna; (**g**) *M. longiciliatum*, female fore wing; (**h**) *M. longiciliatum*, female dorsal meso-and metasoma.

***Megaphragma longiciliatum*Subba Rao** (Figure 5e–h and Figure 16e)

*Megaphragma longiciliatum* Subba Rao, 1969. *Proc. R. Ent. Soc. Lond. (B)* 38(7–8): 114.
*Megaphragma aligarhensis* Yousuf and Shafee, 1988. *Indian J. syst. Ent.* 4(2) [1987]: 114. **Syn. nov**.
*Megaphragma amalphitanum* Viggiani in Viggiani and Bernardo, 1997. *Boll. Zool. Agr. Bach. Ser. II* 29(1): 51–55. **Syn. nov**.
*Megaphragma magniclava* Yousuf and Shafee, 1988. *Indian J. syst. Ent*. 4(2) [1987]: 115–116. **Syn. nov**.
*Megaphragma decochaetum* Lin, 1992. *Entomotaxonomia* 14(2): 131–132. **Syn. nov**.
*Megaphragma shimalianum* Hayat, 2009. *Oriental Insects* 43: 212–213. **Syn. nov**.

*Diagnosis. Female*: Antenna (Figure 5e and Figure 16e) with clava two-segmented. Funicle with ≥2 **MT**; **C2** with ≥6 **MT**, 1 **UST**; **C3** with ≥3 **MPS**, and 1 **UST**.

Mid lobe of mesoscutum anteriorly with reticulate sculpture. Propodeum with a very short central area. Fore wing 8× as long as wide (Figure 5g). Metasoma (Figure 5h) without subpolygonal sculpture on tergites, but with some ridges, **T2**–**T4** each with a pair of long setae. Ovipositor 1.1× as long as mesotibia.

Body brown to dark brown, with the following paler: antenna, legs. Metasoma with tergites and sternites appearing as dark bands (in the slide-mounted types). Fore wing completely hyaline.

*Male*: Similar to female in most characters except genitalia; antennal funicle slightly more elongate than in female, and clava darker than remainder of antenna. **C2** without long **UST**; **C3** shorter than in female (Figure 5f).

*Material examined*. Holotype ♀ *M. longiciliatum* (NHMUK). **INDIA**: Bangalore, Avati, ex. *Frankliniella lilivora* Takahashi on *Polyanthes tuberosa*, x.1968, V. P. Rao. Paratypes: 13♀ 1♂, same data as holotype (NHMUK).

Holotype ♀ *M. aligarhensis* (AMU). **INDIA**: Aligarh, IX.1985, M. Yousuf.

Holotype ♀ *M. amalphitanum* (DACE). **ITALY**: Vietri sul mare (SA), x.1994, coll. G. Viggiani, ex egg of *Heliothrips haemorrhoidalis* on *Viburnum tinus*. Paratypes: 36♀, 32♂, mostly obtained from the same host collected in the same holotype locality (DACE).

Holotype ♀ *M. decochaetum* (FAFU). **CHINA**: Fuzhou, Fujian, 30.vi.1987, coll. Lin. Paratype: CHINA: Fuzhou, Fujian, 30.vi.1987, coll. Lin (1♂, FAFU).

Holotype ♀ *M. magniclava* (AMU). **INDIA**: Aligarh, 25.x.1985, M. Yousuf.

Paratypes *M. shimalianum*. **INDIA**: Uttar Pradesh, Mainpuri Malau, slide XIV 1, 2, 4, 6.ix.2007, F. R. Khan col. (3♀, 1♂, AMU); Firozabad, Nagla Prabhu, slide IX, 4.ix.2007, F. R. Khan col. (12♀, AMU).

Non-types: **ARGENTINA**: INTA Oliveros Santa Fe, v.2004, ex *Caliothrips phaseoli*, A. M. Molinari col. (2♀, 7♂, DACE, AICF); San Miguel de Tucuman, x–xi.2006, ex Thysanoptera eggs on corn, E. Luft col. (2♂, DACE, AICF); Salta Prov., Aguas Blancas, Routa 19, 22.72° S, 64.40° W, 447 m, 23.iii.2003, swp rainforest along Bolivia border, J. Munro 003-03-23-01 (1♀, UCRC); Salta Prov. Rosario de la Frontera (grounds of Hotel Termas), 25.84° S, 64.93° W, 447 m, 20.iii.2003, sweeping, J. Munro 003-03-20-10 (1♀, UCRC); Salta Prov., RN81, 66 km E. jct RP 24, 23.24° S, 63.40° W, 260 m, 24.iii. 2003, swp Dry Chaco, J. Munro 003-03-24-01 (6♀, AICF, UCRC). **AUSTRALIA**: WA, Margaret R, Warner Glen Rd, Stone Cottages, 34°04.44′ S, 115°08.14′ E, eucalyptus forest, YPT, 15–16.xi.2002, George, Owen, Hawks, Munro PEET02-010P (1♀, UCRC). **CHINA**: 24.v.1987 and 26.v.1987, coll. Lin, identified as *M. decochaetum* (1♀, 1♂, FAFU). **D. R. CONGO**: Province Orientale, Yangambi Biosphere Reserve 0°45.822′ N 24°30.285′ E, 15.v.2012, screen sweep primary forest, A. Polaszek col. BMNH 2012-88, DNA: COM 2.1 and COM 2.3 (2♀, AICF, NHMUK). **FRANCE**: Dept Gironde, St Colombe (nr Castillon-la-Bataille), Pitray, 1.viii.2000, S. Bessart, M. van Helden (3♀, UCRC); Dordogne, 3.5 km E Issigeac, 44°43′ N 0°38′ E, 100 m, 31.vii.2013, J.S. Noyes col. NHM(Ent.) 2013-144, DNA: FRM2 to FRM6 (4♀, 1♂, AICF, NHMUK). **HUNGARY**: Őrség Nemzeti Park, Barkás Lake, 46°52′ N 16°26′ E, 268 m, 28.vi.2010, screen-sweep, J.S. Noyes col., BMNH(Ent) 2010-63, DNA: HUM1 (1♂, NHMUK). **INDIA**: Uttar Pradesh, New Delhi, IARI, 220 m, 28°37′51″ N 77°09′50″ E, 5–7.xi.2003, pan trap, J. Heraty col. (1♀ 4♂, UCRC); Karnataka, W of Mudigere, 850–912 m, 13°07′05″ N 75°30′20″ E, 24.xi.2003, sweep evergreen forest, J. Heraty col. (3♀, UCRC). **INDONESIA**: W Java, Gunung Halimun NP, Tea-Forest Junction, 1066 m, 6°41′07″ S 106°31′16″ E, 17.ix.2015, screen-sweep, A. Polaszek col., DNA1147 (1♀, NHMUK). **ITALY**: Vietri sul mare, Benincasa, 40°40′ N 44°20′ E, 17.vii.2013, ex *Heliothrips haemorrhoidalis* on *Viburnum tinus*, G. Viggiani, DNA: ITM10 (1♀, NHMUK). **MALAYSIA**: Sarawak, Mentawai 4°14′ N 114°52′ E, ix.2011, screen sweep, A. Polaszek col., DNA: SRM1 (1♀, NHMUK). **OMAN**: Hajar Mts, screen-sweep, 20.i.2017 A. Polaszek col., DNA: MO3, MO13, MO20, MO22 (MO13 was destroyed during the DNA extraction) (2♀ 1♂, NHMO). **PAPUA NEW GUINEA**: Central Province, 15km SE Port Moresby, 1.i.1986, screen-sweep eucalyptus grassland, G. Gordh col. 86-01-01-1 (1♀, ANIC). **UNITED ARAB EMIRATES**: Abu Dhabi Emirate, Al Ain, Al Khabisi garden, 24°13.521N 55°41.95E, 25–30.iii.2019, yellow pan trap, A. Polaszek, B. Howarth col. (1♀, NHMUK). **USA**: Florida, Lake Seminole Park, Seminole, 27°50–51′ N 82°46′ W 9.vii.2015, sweep, Z. Lahey col., DNA1111, 1112, 1113 (3 specimens, NHMUK, currently misplaced).

*Species-group placement*: *M. longiciliatum*-group.

*Distribution*: Argentina, Australia, China, D. R. Congo, France, India, Indonesia (Java), Italy, Malaysia (Borneo, Sarawak), Oman, Papua New Guinea, Portugal [62] (as *M. amalphitanum*), UAE, and USA.

*Hosts*: *Caliothrips phaseoli* (Hood) (Argentina); *Frankliniella lilivora* Takahashi (India); *Heliothrips haemorrhoidalis* (Bouché) (Italy). The record from Argentina “ex Thysanoptera eggs on corn” could be from *Frankliniella williamsi* Hood.

*DNA data*: CO1: 8 sequences from 4 countries: D. R. Congo, France, Hungary, Malaysia (Sarawak); 28S: 18 sequences from 8 countries: D. R. Congo, France, Hungary, Indonesia (Java), Italy, Malaysia (Sarawak), Oman, USA.

*Comments*: *Megaphragma longiciliatum* is the most widely distributed *Megaphragma* species; hence the large number of synonyms. We have examined 150 specimens from 14 countries and have DNA sequences for 18 specimens from 8 very widely distributed countries. We have carefully assessed morphological variation within the specimens examined, and consider that it encompasses the morphological characteristics of the type material of the species synonymized above [7,9,10,63,64].

The holotype of *M. longiciliatum* is in extremely poor condition. The mountant, presumably gum chloral, has turned black. It is to be hoped that in a few years’ time, the holotype will be destroyed completely, and one of the paratypes, all of which are still in excellent condition, can be designated a neotype. Unfortunately, there is no current provision under the Code to legitimately replace a holotype specimen that has deteriorated irremediably.

**Figure 6:**
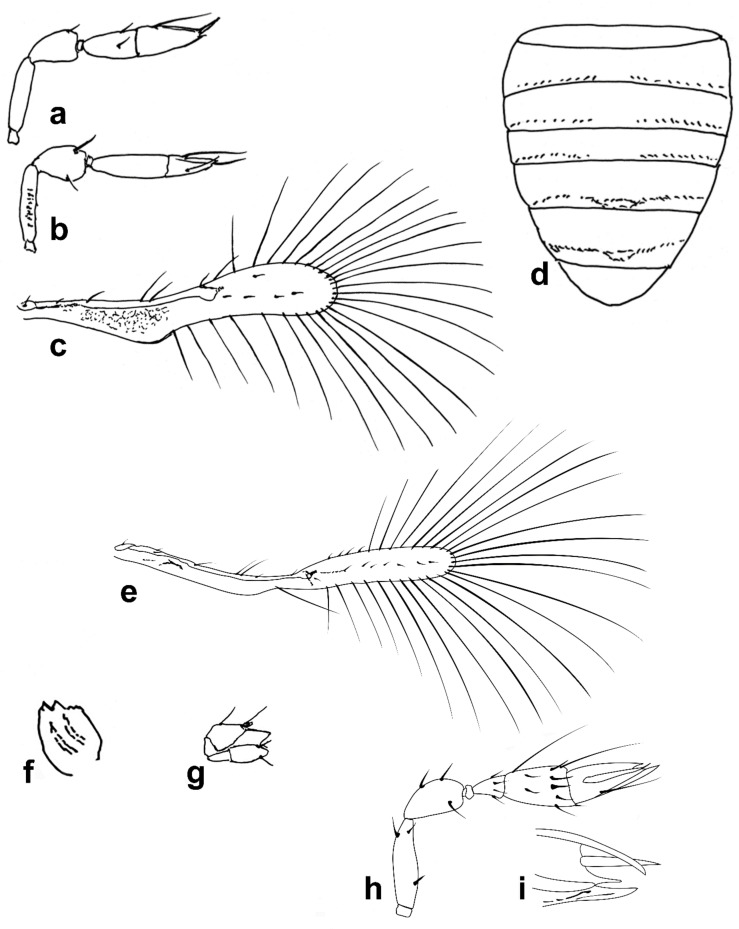
Line drawings of *Megaphragma* species: (**a**) *M. macrostigmum*, female antenna (Paratype); (**b**) *M. macrostigmum*, male antenna (Holotype); (**c**) *M. macrostigmum*, female fore wing (Paratype); (**d**) *M. macrostigmum*, male dorsal metasoma (Holotype); (**e**) *M. mymaripenne*, female fore wing (Holotype); (**f**) *M. mymaripenne*, mandible (Holotype); (**g**) *M. mymaripenne*, maxillary palp (Holotype); (**h**) *M. mymaripenne*, female antenna (Holotype); (**i**) *M. mymaripenne*, female antenna (detail of apex) (Holotype).

***Megaphragma macrostigmum* (Lin)** (Figure 6a–d)

*Paramegaphragma macrostigmum* Lin, 1992. *Entomotaxonomia* 14(2): 135–136.

*Megaphragma macrostigmum*: Delvare, 1993. *Revue fr. Ent. (n.s.)* 15(4): 151.

*Diagnosis*. *Female*: Antenna without funicle and clava two-segmented (Figure 6a); **C1** with two short **MT**; **C2** with one **MPS**, one **SB**, and one **UST**.

Mid lobe of mesoscutum with some large, but shallow polygonal cells. Propodeum with a very short central area. Fore wing 5.3× as long as maximum discal width, with two rather long setae in the middle of marginal vein (Figure 6c); disc with 4–5 setae not in a row. Metasoma with a line of microspines evident on **T2**–**T6** (cf Figure 6d). Ovipositor 2.1× as long as mesotibia. The main features of the antenna and fore wing are illustrated in Figure 6a,c.

Body uniformly pale brown, fore wing slightly to moderately infuscate below marginal vein.

*Male*: Similar to female in most characters except genitalia; antennal funicle slightly shorter than in female, **C1** longer, **C2** with long **UST** (Figure 6b).

*Material examined*. Holotype ♂ (FAFU). **CHINA**: Fuzhou, Fujian, 31.viii.1987, N.Q. Lin col.

Paratype: **CHINA**: Guangzhou, 3.xi.1985 N.Q. Lin col. (1♀, FAFU)

Non-type: **CHINA**: Guangzhou, 30.x.1985, N.Q. Lin col. (1♂, FAFU).

*Species-group placement*: *M. macrostigmum* group—possibly a subgroup of the *M. longiciliatum*-group.

*Distribution*: China.

*DNA data*: no DNA sequences.

*Comments*: This species is very similar to *M. caribea*; at present, the only difference from the latter species appears to be the absence of long sensilla on **C2**.

***Megaphragma mymaripenne* Timberlake** (Figure 1a, Figure 6e–i and Figure 17d)

*Megaphragma mymaripenne* Timberlake, 1924. *Proc. Haw. Entomol. Soc*. 5: 414–415.
*Megaphragma mymaripenne*: Viggiani, 1997. *Boll. Lab. Ent. agr. Filippo Silvestri* 53: 117–122.

*Diagnosis*. *Female*: Antenna (Figure 6h and Figure 17d) with clava two-segmented, funicle with ≥4 **MT**; **C1** trapezoid in lateral view with length 1.5× maximum width or less (longer in dorsal or ventral view), with 2 **UST**, but without linear sensilla (**MPS**), ≥9 **MT**; **C2** with ≥3 **MPS**, 1 **MT**, 1 **SB**, and 1 **SS** (Figure 6i).

Mid lobe of mesoscutum anteriorly with subpolygonal sculpture, but often appearing smooth in slide-mounts. Propodeum with a very short central area. Fore wing (Figure 6e) 9–10× as long as wide, marginal vein with two long setae in the middle, setae on disc more or less regularly in a row of 10–15 setae, and longest fringe seta 5–6× as long as maximum disc width. **T1** with sculpture represented by a combination of transverse and longitudinal cells, lateral ones twice as long as wide; sides of some cells with denticles present. The subsequent tergites show rather variable sculpture, differing from the pattern on the first tergum. **T2**–**T4** each with a pair of very short setae.

Body uniformly pale brown, scutellum paler than mesoscutum. Legs pale, wings hyaline. Clava slightly darker than the remainder of the antenna.

**Figure 7:**
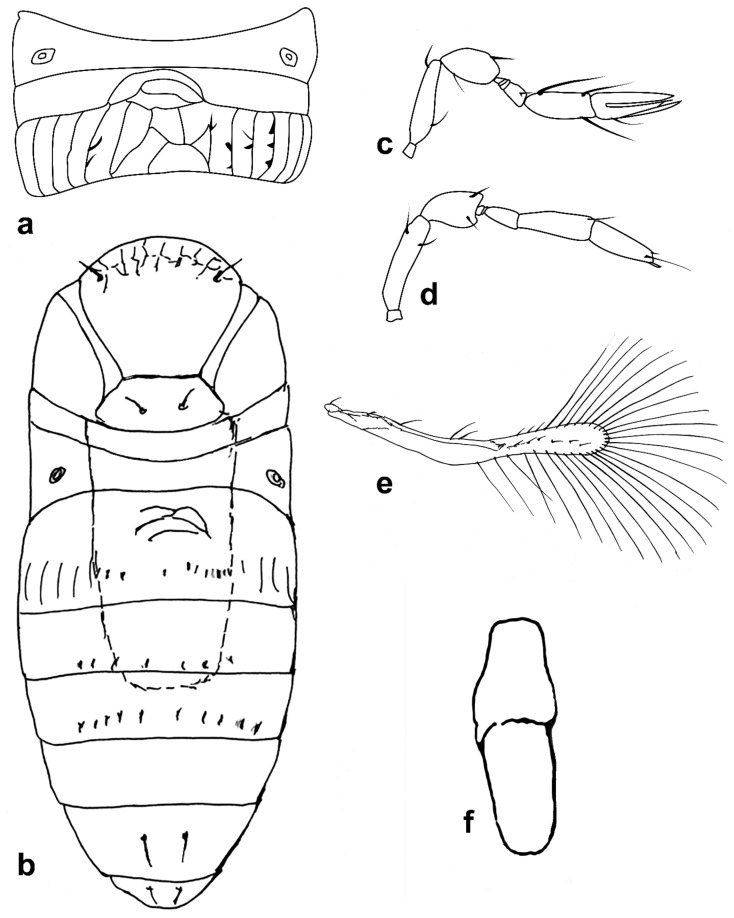
Line drawings of *Megaphragma* species: (**a**) *M. nowickii*, female propodeum and **T1** (Holotype); (**b**) *M. nowickii*, male meso- and metasoma (Paratype); (**c**) *M. nowickii*, female antenna (Holotype); (**d**) *M. nowickii*, male antenna (Paratype); (**e**) *M. nowickii*, female fore wing (Holotype); (**f**) *M. nowickii*, male aedeagus (Paratype).

*Male* (hitherto undescribed): same as female but antenna slender, with funicle twice as long as wide and **C1** about 1.7× as long as **C2**. **T1** with sculpture not as complete as in the female. Genitalia simple, tubular, 4.5× as long as wide (cf Figure 7f).

*Material examined*. Holotype ♀ (USNM). **USA**: Hawaii, Mountain View, i.1920, C.E. Pemberton col.

Paratype: same data and on the same slide with holotype (1♀ USNM).

Non-types: **ARGENTINA**: San Miguel de Tucuman, x–xi.2006, ex Thysanoptera eggs on corn, E. Luft col. (5♀, DACE); ix.2006, from corn, E. Vinla col. (3♂, DACE); Salta Prov., Rosario de la Frontera, 25.83° S 64.88° W, 745 m, 20.iii.2003, sweep forest, J. Munro 003-03-20-01 (1♀, UCRC); La Rioja Prov., Chuquis, 28°53′40″ S 67°00′31″ W, 1575 m, 17.iii.2003, sweep acacia scrub, J. Munro 003-03-17-05 (1♀, UCRC); Salta Prov., Orán, rd to San Andres along Rio Blanca, 23.11° S, 64.52° W, 535 m, 23.iii.2003, sweep scrub and ginger, J. Munro 003-03-23-02 (2♀, UCRC). **BRAZIL**: Santa Catarina, Nova Teutonia, 17.x.1949, F. Plaumann col. BM 1957-341 (1♀, NHMUK). **COSTA RICA**: Limón, Hitoy-Cerere Reserve, 9°40′ N 83°02′ W, 100 m, 24–26.ii.2008, J.S. Noyes col. NHM(E)2010-21AQ (1♀, NHMUK). **DOMINICAN REPUBLIC**: San Cristobal, S. Cristobal Manomatuey, 20 km NW valley, 500 m, 23.iii.1991, L. Masner col. (1♀, UCRC). **ECUADOR**: 1♀, Galapagos Is., Sta Cruz, Bellavista 2 mi N, 360 m, guava thicket, v–vii.1985, S. and J. Peck col. (CNCI). **GUADELOUPE**: Petit Borg, Domaine Duclos, 28.ii.1989, with *Solenothrips rubrocinctus* and *Heliothrips haemorrhoidalis* on *Inga ingoides*, J. Etienne col. (3♀, NHMUK). **ISRAEL**: Bet Dagan, ix.1996, ex *Heliothrips haemorrhoidalis*, M. Wysoki col. (7♀, DACE, AICF). **MEXICO**: Chiapas, 6.2 miles N Berriozabal, premontane rain forest, 9.viii.1990, 4000′ J.B. Woolley col. (3♀, 2♂, UCRC). **USA**: California, Orange Co., Irvine, 13.vi.1990, ex *Heliothrips haemorrhoidalis* on avocado, H.G. Johnson (1♀, NHMUK); California, Orange Co., South Coast Field Station, El Toro, ex *Heliothrips haemorrhoidalis* on avocado, H.G. Johnson (2♀, 1♂, UCRC); California, Orange Co., 10.ix.1989, ex *Heliothrips haemorrhoidalis* on avocado N. Hessein col. (1♀, DACE); Virginia, Montgomery Co., 8 km NW Blacksburg, 19–30.vi.1987, **MT**, rural, 1000 m, BRC HYM. TEAM (1♀, CNCI); California, San Diego Co., Valley Center, Weslilac Rd, Playa Grove, on avocado, H.G. Johnson (7♀, UCRC); Missouri, Parkville, 39°12′17″ N, 94°40′38″ W, 5.vii.2015, swept, Z. Lahey col., DNA: 1114 (1♀, NHMUK—currently misplaced).

*Species-group placement*: *M. mymaripenne*-group.

*Distribution*: Argentina, Brazil, Chile, Costa Rica, Dominican Republic, Ecuador, Guadeloupe, Israel, Mexico, USA, and Venezuela.

*Hosts*: *Megaphragma mymaripenne* is a solitary egg endoparasitoid of several species of Panchaetothripinae (Thripidae). The most common host is the widespread *Heliothrips haemorrhoidalis*. The populations recorded in the USA [12] are represented mainly by females. The population reared in Argentina from maize and identified as *M*. *mymaripenne* [11] differs from the known populations of the species: the reared specimens from maize appear to be normally bisexual.

*DNA data*: 28S: 1 sequence, Missouri (USA).

*Comments*: This species was described in detail by Timberlake [65], and additional features were given by Viggiani [6]. *Megaphragma mymaripenne* is extremely difficult to distinguish morphologically from the closely related species *M. polilovi*, and even from the more distantly related species *M. noyesi*, with which it has been previously confused. They differ, however, in the length and shape of **C1**, length of the scape and colour of the radicle, and length of the ovipositor, respectively, as outlined in the key. Without the molecular data, these subtle differences would have been overlooked or treated as intraspecific variability. The correlation between the molecular clades and morphological characters indicates, however, that there are three species involved.

Records from Israel are the only Old-World records for this species; previous records of *M. mymaripenne,* e.g., from Italy [13,14,66], turned out to be misidentifications of the new species *M. polilovi*.

**Figure 8:**
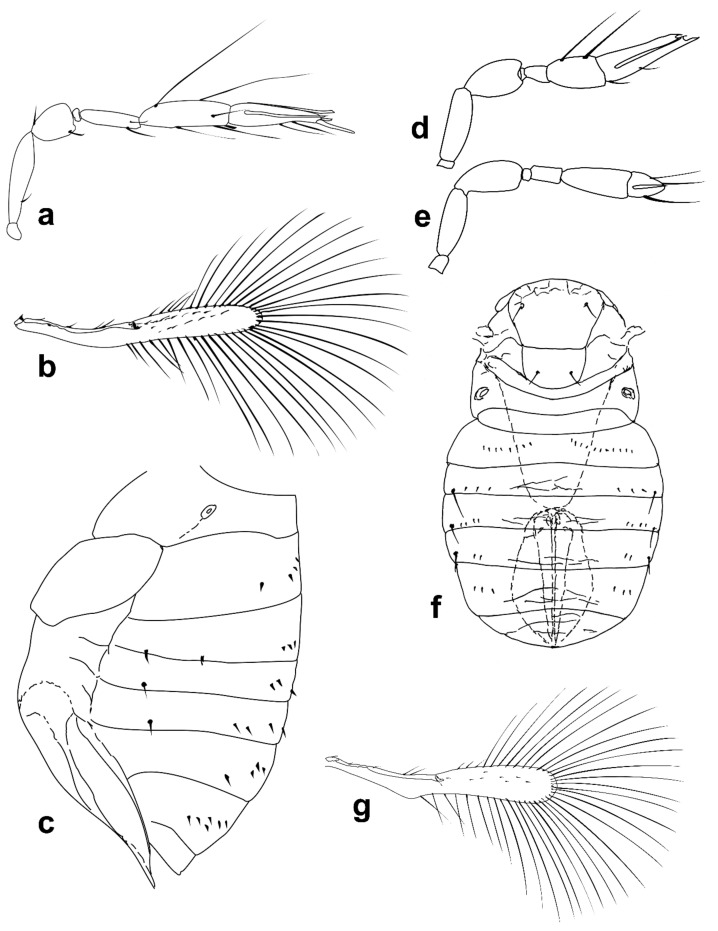
Line drawings of *Megaphragma* species: (**a**) *M. polychaetum*, female antenna (Paratype); (**b**) *M. polychaetum*, female fore wing (Paratype); (**c**) *M. polychaetum*, female propodeum and metasoma, lateral view (Paratype); (**d**) *M. priesneri*, female antenna (Neotype); (**e**) *M. priesneri*, male antenna (non-type); (**f**) *M. priesneri*, female dorsal meso- and metasoma (Neotype); (**g**) *M. priesneri*, female fore wing (Neotype).

***Megaphragma polychaetum* Lin** (Figure 8a–c)

*Megaphragma polychaetum* Lin, 1992. *Entomotaxonomia* 14(2): 132–133.
*Megaphragma anomalifuniculi* Yuan et Lou in Yuan et al., 1997. *Journal of Northeast Normal University* 4: 62–63. **Syn. nov**.

*Diagnosis*. *Female*: Antenna long and narrow (Figure 8a) with pedicel shorter than the subcylindrical funicle, which has two **MT**. Clava two-segmented, **C1** twice as long as funicle, with two **MT** and two long **UST**; **C2** with two **MPS**, two **MT**, and a terminal basiconic sensillum (**SB**) slightly shorter than half **C2** length.

Mid lobe of mesoscutum with subpolygonal sculpture. Propodeum with a very short central area (Figure 8c). Fore wing (Figure 8b) 8–9× as long as wide, with two short setae in the middle of the marginal vein, and a disc with two distinct rows of 6–8 setae (Figure 8b). Tergites of metasoma without sculpture, but with some short and strong setae. The ovipositor is 1.1× as long as the mesotibia.

Head (including antenna), metasoma, meso-, and metacoxae are very dark. Remainder of body, including legs, pale brown. Fore wing strongly infuscate basally.

*Male*: Unknown.

*Material examined*. Paratypes: **CHINA**: Wuyishan, Fujian, 30.vii.1987, Wang Jiashe col. (3♀, FAFU); 10.vii.1987, 14-051, 14-052 (2♀, FAFU).

*Species-group placement*: *M. polychaetum*-group.

*Distribution*: China.

*DNA data*: no DNA sequences. DNA sequences are very likely to be close to those of *M. cockerilli***sp. nov.** (see below).

*Comments*: The type material of *M. anomalifuniculi* was not available to the authors. According to the illustration given by Yuan and Lou [67], *M. anomalifuniculi* appears to be similar, if not identical, to *M. polychaetum* Lin. The features concerning the funicular segment appear to derive from a preparation artifact.

***Megaphragma priesneri* (Kryger)** (Figure 8d–g and Figure 22e)

*Sethosiella priesneri* Kryger, 1932. *Bulletin de la Société Royale d’Egypte* 16: 40.
*Megaphragma priesneri*: Ghesquière, 1839. *Rev. Zool. Bot. Afr*. 33(1): 38.

*Diagnosis*. Female: Antenna (Figure 8d) with pedicel slightly shorter than scape, funicle as long as half pedicel. Clava two-segmented with two long **UST** on **C1** of female; **C2** with one **MPS** and two **MT**.

Mid lobe of mesoscutum (Figure 8f) anteriorly with subpolygonal sculpture; propodeum with a very short central area. Fore wing (Figure 8g) 7× as long as wide, with maximum distal width less than 2× width measured at apex of marginal vein (Figure 8g); maximum fringe seta length 4× maximum discal width; setae on ventral disc surface short, penultimate one not reaching to the base of the distal (Figure 22e). **T1** without sculpture, but with a row of microspines; **T2**–**T4** each with a pair of setae, shorter than their corresponding tergites. Ovipositor 1.1× mesotibia.

Entire head and body are very dark. Legs and antenna paler. Wings hyaline.

*Male*: Similar to female in most characters except genitalia. Antenna with funicle and **C1** more elongate than in female, without **UST** on **C1**; **C2** much shorter than in female (Figure 8e).

*Material examined*. Neotype ♀ (NHMUK), here designated. **EGYPT**: Tanta, 30.11.30, vine leaves with *Retithrips*.

Non-types: **ISRAEL**: Higwe Yisrael, xi.1996, M. Wysoki coll., ex eggs *Retithrips syriacus* on *Vitis vinifera* (15♀, 3♂, NHMUK, DACE, AICF).

*Species-group placement*: *M. longiciliatum*-group.

*Distribution*: Egypt, Israel.

*Host*: *Retithrips syriacus* (Mayet).

*DNA data*: no DNA sequences.

*Comments*: Following extensive inquiries over the decades since 1990 in Egypt and Denmark, the holotype (and indeed the remainder of the type series of four specimens) appears to be lost. A specimen with data almost identical to the holotype is in the NHMUK, but has aberrant antennae. Nevertheless, we here designate that specimen as neotype, given that the data are very similar to those of the original type [68] (only the collection date differs by less than a month). Furthermore, all of the remaining morphology accords perfectly with the original description. Unfortunately, extensive efforts to collect fresh specimens in both Egypt and Israel failed.

The neotype designation for *M. priesneri* (Kryger) satisfies the provisions of Article 75.3 of the International Code of Zoological Nomenclature by: (1) clarifying the taxonomic identity of the species in its accepted modern concept (Article 75.3.1); (2) defining the combination of features of the sculpture of the mesoscutum and **T1**, propodeal structure and wing proportions as diagnostic for the species (Article 75.3.2); (3) providing data and description sufficient to ensure recognition of the specimen designated (Article 75.3.3); (4) giving reasons (no references available heretofore) for believing that the original type material is lost (Article 75.3.4); (5) selecting a neotype specimen consistent with the original description of the species and that was collected not long (less than 1 month) after the original description (specimen in this case) and, as such, represents the type species (Article 75.3.5); (6) choosing a neotype from the originally cited type locality, Tanta, Egypt (Article 75.3.6); and (7) recording that the neotype is the property of a recognized scientific institution, NHMUK in London (Article 75.3.7).

**Figure 9:**
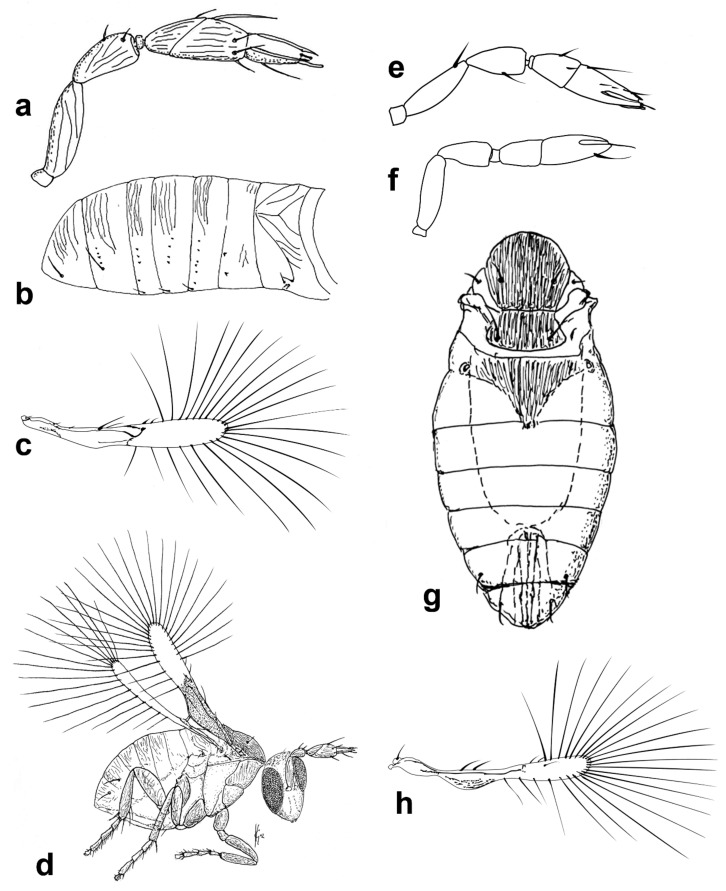
Line drawings of *Megaphragma* species: (**a**) *M. rivelloi*, female antenna (Holotype); (**b**) *M. rivelloi*, female metasoma (Holotype); (**c**) *M. rivelloi*, female fore wing (Holotype); (**d**) *M. rivelloi*, female habitus (Holotype); (**e**) *M. stenopterum*, female antenna (Paratype); (**f**) *M. stenopterum*, male antenna (Paratype); (**g**) *M. stenopterum*, female dorsal meso- and metasoma (Paratype); (**h**) *M. stenopterum*, female fore wing (Paratype).

***Megaphragma stenopterum* (Lin)** (Figure 9e–h)

*Paramegaphragma stenopterum* Lin, 1992. *Entomotaxonomia* 14(2): 134–135.
*Megaphragma stenopterum*: Delvare, 1993. *Revue fr. Ent. (n.s.)* 15(4): 151.

*Diagnosis*. *Female*: Antenna (Figure 9e) without funicle, clava two-segmented, and **C2** twice as long as **C1**. **C1** with two **MT**; **C2** with one **MPS**, two **MT**, one **SB**, and one **UST**.

Mid lobe of mesoscutum, scutellum, and central area of propodeum longitudinally striate (Figure 9g). Fore wing (Figure 9h) 9× as long as wide, and longest fringe seta 7× as long as maximum discal width, with two long central setae on the marginal vein, one long discal seta; hind margin sinuate. Tergites of metasoma without sculpture or crenulae (Figure 9g).

Body brown, the head darker brown. Fore wing basally strongly infuscate.

*Male*: Almost no discernible differences from female except genitalia characters. Even the antennae are very similar (Figure 9e,f).

*Material examined*. Paratype: **CHINA**: Fuzhou, Fujian, 20.xii.1987, N.Q. Lin col. (1♀, FAFU).

Non-types: **CHINA**: Fuzhou, Fujian, 6.x.1987, N.Q. Lin col. (1♀, FAFU); Fuzhou, Fujian, 20.vi.1987, N.Q. Lin col. (1♂, FAU).

*Species-group placement*: *M. stenopterum*-group.

*Distribution*: China.

*DNA data*: no DNA sequences.

*Comments*: This species, described in detail by the author, has the unique combination of a single seta on the fore wing and four-segmented antenna without any apparent funicle. A transverse, anelliform funicle is present in *M. uniclavum*, the only other species with a four-segmented antenna *and* a single seta on the fore wing.

There are differences between the Chinese text and the English text of the original description concerning the collecting dates of the type series. The examined paratype is mentioned in the English part but not in the Chinese part.

**Figure 10:**
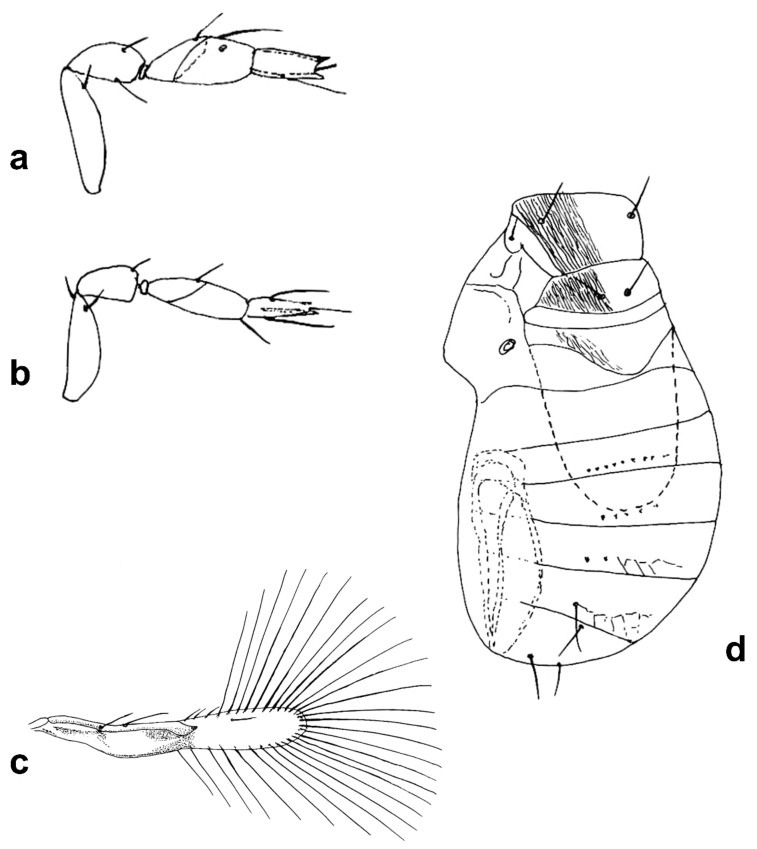
Line drawings of *Megaphragma* species: (**a**) *M. striatum*, female antenna (Holotype); (**b**) *M. striatum*, male antenna (Paratype); (**c**) *M. striatum*, female fore wing (Holotype); (**d**) *M. striatum*, female dorsal meso- and metasoma (Holotype).

***Megaphragma striatum* Viggiani** (Figure 10a–d and Figure 21b,c)

*Megaphragma striatum* Viggiani, 1997. *Boll. Lab. Ent. Agr. Filippo Silvestri* 53: 119–120.

*Diagnosis*. *Female*: Antenna (Figure 10a and Figure 21b) without funicle, clava three-segmented, with **C1** having a transverse distal margin. **C1** with ≥1 **MT**; **C2** ≥8 **MT**, and 2 **UST**; **C3** with 2 **MPS**, ≥2 **MT**, and 1 **SB**.

Mid lobe of mesoscutum and scutellum longitudinally striate (Figure 10d). Propodeum with a pronounced subtriangular central area (Figure 21c). Fore wing 8× as long as wide, with one long central seta on the marginal vein, one discal seta, longest fringe seta 4–5× as long as maximum discal width. Metasoma with a row of crenulae on **T2** (Figure 21c).

Head and metasoma very dark, mesoscutum brown, the remainder, including legs and antenna, paler. Fore wing infuscate basally.

*Male*: Similar to female in most characters except genitalia.

*Material examined*. Holotype ♀ (DACE). **MEXICO**: Chiapas, Ocozocoautla, El Aquacero, 1800–2200′, 8.8.1990, coll. JB Woolley.

Paratypes: 2♀, 2♂, same data as holotype (UCRC).

Non-types: **MEXICO**: Tamaulipas, Alta Cima (nr Goméz Farias), 23°01′ N 99°09′ W, 2.xi.2009, screen-sweep A. Polaszek col., DNA: MXM1 (1♀, NHMUK); 1♀, 1♂, same data as holotype, but not mentioned in the original description (CNCI, UCRC).

*Species-group placement*: *M. ghesquierei*-group.

*Distribution*: Argentina, Belize, Costa Rica, Mexico.

*DNA data*: CO1: one sequence; 28S: one sequence (both Mexico).

**Descriptions of new species**

**Figure 11:**
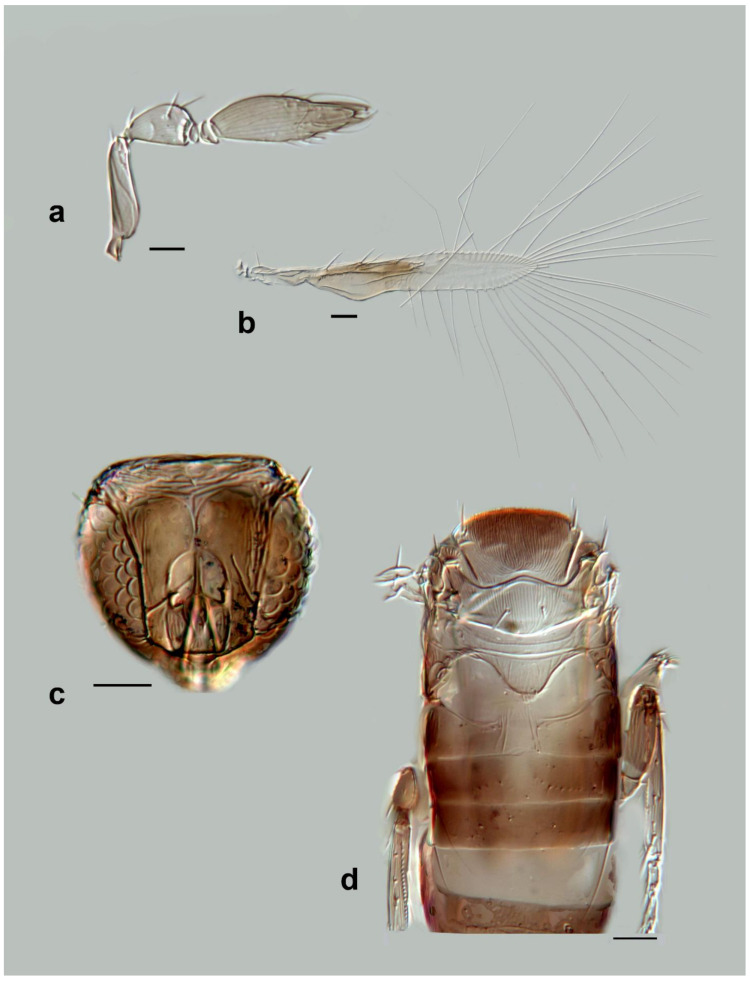
Photographs of *Megaphragma* species: (**a**) *M. antecessor*, female antenna (Holotype); (**b**) *M. antecessor*, female fore wing (Holotype); (**c**) *M. antecessor*, female head (Holotype); (**d**) *M. antecessor*, female dorsal meso- and metasoma (Holotype). Scale bars 20 µm.

***Megaphragma antecessor* Polaszek and Fusu sp. nov.** (Figure 11a–d)

urn:lsid:zoobank.org:act:A68D7B18-ADE4-4EDC-BB04-32A77E063469

*Description. Female*: Head (Figure 11c) with toruli vertical, in contact with each other medially; area below toruli with fine longitudinal sculpture, 1 min seta present laterally on each side. Antenna (Figure 11a) five-segmented (excluding anellus), transverse funicle present; clava two-segmented but these almost completely fused. **C1** with ≥6 **MT**, 3 **UST**; **C2** with 3 **MPS** and 2 **UST**; **SB** not detected but presumably present. Base of **C2** with one (apparent) **SS**.

Mid lobe of mesoscutum (Figure 11d) with fine longitudinal striation; vertical/ventral anterior mid lobe of mesoscutum with coarse, reticulate sculpture (Figure 11d); propodeum with subtriangular area centrally (Figure 11d) with 3–4 large crenulae; propodeum with hind margin arcuate. Fore wing (Figure 11b) 8.5× as long as maximum width, maximum distal width is 92× the maximum basal width; disc with a single short seta, and longest fringe seta 6.5× maximum discal width. Marginal vein with four setae, the second (from the wing base) robust and blunt; central setae equal in length. Campaniform sensillum present below second seta, a line joining the sensillum to the fourth seta. Stigmal vein with a row of three campaniform sensilla apically. Mesotibia with two large spines basally; metafemur with spine; metatibia with a row of fine, blunt setae extending almost the entire inner length, increasing abruptly in length at the distal tibia (exact length not visible in Figure 11d since setae positioned almost vertically; a similar row of setae is found in *M. momookherjeeae* and *M. uniclavum*, Figure 21j). **T1** with smooth area centrally, flanked by two or three longitudinal grooves and a longitudinal cell laterally, extending for the length of the tergum; **T1**–**T4** with very long setae laterally, each longer than its tergum; **T2** with a curved row of 6–8 spicules on each side. Ovipositor 1.6× as long as mesotibia.

Body brown. Occiput and face entirely brown, vertex paler. Antenna pale brown, pedicel paler. Mesosoma with the following brown: mid lobe of mesoscutum centrally, side lobes, axillae, propodeum laterally; remainder of mesosoma pale. Entire metasoma brown, except **T1** centrally pale. Fore wing distinctly infuscate basally, below, and including marginal vein.

*Male*: Unknown.

*Material examined*. Holotype ♀ (deposited in NHMUK). **COSTA RICA**: Puntarenas, Est. Biol. Monteverde, 10°19′ N 83°49′ W, 1540–1890 m, 26.ii.2007, J.S. Noyes BMNH(E) 2010-21, DNA: CRM2.2.

*Species group placement*: *M. antecessor*-group. *Megaphragma antecessor* clusters in different DNA sequence analyses with *M. momookherjeeae* **sp. nov.** (CO1 and combined analysis, strong support) or as basal to all other *Megaphragma* (28S, weak support). The shapes of the fore wing, propodeum, and to some extent the antenna, are strongly suggestive of the *ghesquierei*-group;presumably unique aspects of both DNA sequences prevent it from clustering with the species in that group.

*Distribution*: Costa Rica

*DNA data*: CO1: one sequence; 28S: one sequence.

*Etymology*: From the Latin *antecessor* (predecessor, precursor), in reference to the basal position of this species in the phylogenetic analyses. Noun in apposition.

**Figure 12:**
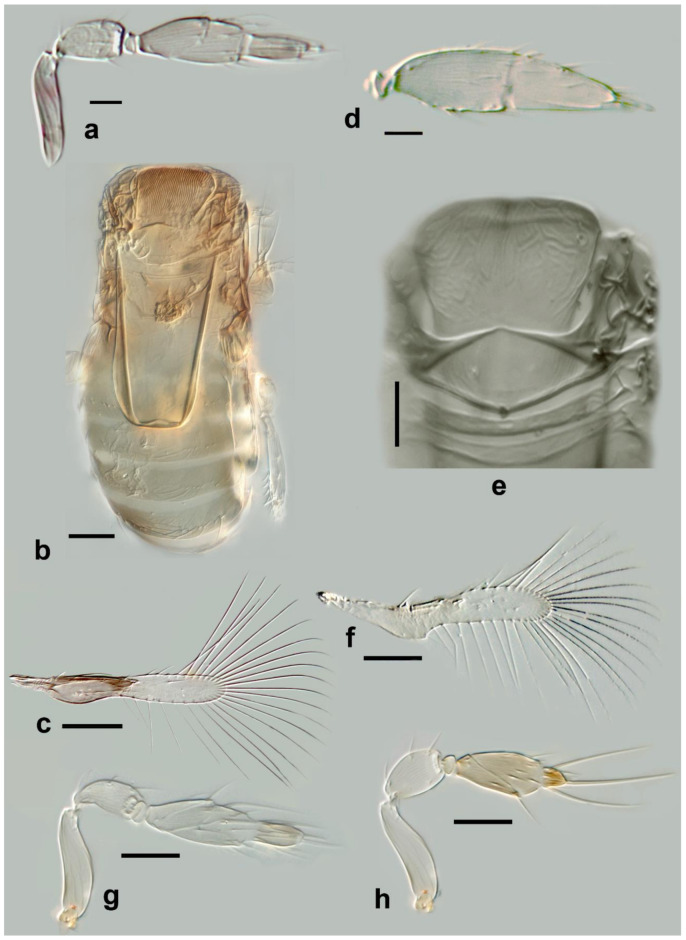
Photographs of *Megaphragma* species: (**a**) *M. breviclavum*, female antenna (Holotype); (**b**) *M. breviclavum*, female dorsal meso- and metasoma, composite image (Holotype); (**c**) *M. breviclavum*, female fore wing (Holotype); (**d**) *M. caribea*, female antenna (flagellum only) (Paratype); (**e**) *M. caribea*, female dorsal mesosoma (Paratype); (**f**) *M. caribea*, female fore wing (Paratype); (**g**) *M. chienleei*, female antenna (Holotype); (**h**) *M. chienleei*, male antenna (Paratype). Scale bars 20 µm except 50 µm for b, c, and f.

***Megaphragma breviclavum* Polaszek and Fusu sp. nov.** (Figure 12a–c)

urn:lsid:zoobank.org:act:FF10048F-0F8B-4B17-AF74-6E7C504C2ED4

*Description. Female*: Antenna (Figure 12a) five-segmented (excluding anellus); funicle absent; hence, clava three-segmented, with **C1** and **C2** almost fused. **C1** without **UST**; **C2** with 2 **UST**, and abundant **MT**; **C3** with 2–3 **MPS**, **SB**, and **SS**.

Mid lobe of mesoscutum (Figure 12b) with longitudinal striate sculpture extending to scutellum; propodeum (Figure 12b) with central area extended posteriorly, crenulae absent. Fore wing (Figure 12c) 7.5× as long as maximum width; the disc with a single long seta; longest fringe seta 4.5× as long as maximum discal width. Marginal vein with one long seta centrally, extending to apex of marginal vein. Stigmal vein moderately enlarged, with four sensilla apically. Mesotibiae with one large spine basally; metafemora with spine. **T1** with elongate cells laterally, 2–3× as long as wide; **T2**–**T4** without setae laterally; **T5** with long setae laterally. Ovipositor 1.7× as long as mesotibia.

Body largely brown, the following paler: legs except coxae and metafemur. Antenna with pedicel pale; scape and **C1**–**C3** darker. Fore wing strongly infuscate basally; stigmal and marginal vein brown; marginal vein very dark centrally.

*Male*: **C1** and **C2** with scattered **SS**; **C2** with 2–3 **MT** apically; **C3** with long apical and ventral **UST**. Colour and morphology largely as in female.

*Material examined*. Holotype ♀ (deposited in NHMUK). **MEXICO**: Tamaulipas, Alta Cima (nr Gómez Farias), 23°01′ N 99°09′ W, 2.ii.2009, A. Polaszek col. NHM(E) 2010-21, DNA: MXM2.

Paratypes: 1♀, 1♂ with same data as holotype, DNA: MXM3 and MXM4 (NHMUK).

*Species-group placement*: *M. ghesquierei*-group.

*Distribution*: Mexico.

*DNA data*: CO1: three sequences; 28S: three sequences.

*Etymology*: A noun in apposition referring to the comparatively short clava.

**Figure 13:**
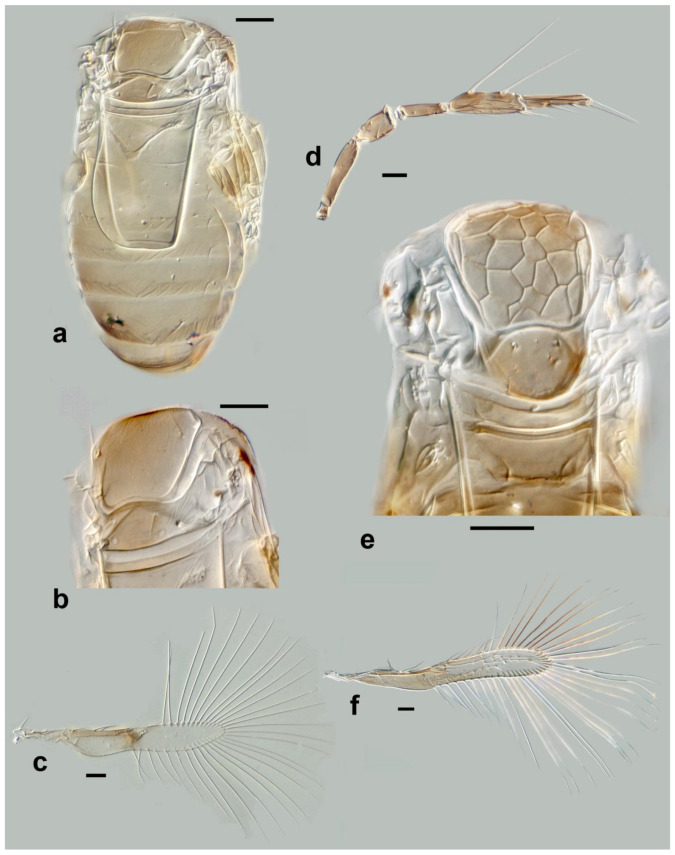
Photographs of *Megaphragma* species: (**a**) *M. chienleei*, male dorsal meso- and metasoma (Paratype); (**b**) *M. chienleei*, male dorsal mesosoma (Paratype); (**c**) *M. chienleei*, male fore wing (Paratype); (**d**) *M. cockerilli*, female antenna (Holotype); (**e**) *M. cockerilli*, female dorsal mesosoma (Holotype); (**f**) *M. cockerilli*, female fore wing (Holotype). Scale bars 20 µm.

***Megaphragma chienleei* Polaszek and Fusu sp. nov.** (Figure 12g,h and Figure 13a–c)

urn:lsid:zoobank.org:act:0ECA37D4-69CF-412F-B081-5F140B3EBA1D

*Description. Female*: Antenna (Figure 12g) five-segmented (excluding anellus); funicle absent (though anellus extremely large); hence, clava three-segmented, with **C1** and **C2** strongly overlapping, almost fused; **C1** with 1 elongate, apical **UST**; **C2** with ≥10 **MT**; and **C3** with 3 very long **UST**.

Mid lobe of mesoscutum (cf Figure 13b) smooth with some irregular longitudinal striate sculpture; propodeum (cf Figure 13a) elongate and curved centrally and posteriorly, crenulae present. **T1** without elongate cells laterally; **T2**–**T4** with short setae laterally. Ovipositor 2× as long as mesotibia. Mesotibia with one large spine basally; metafemur with spine. Fore wing (cf Figure 13c) 8× as long as maximum width, maximum distal width equal to maximum basal width; discal setae absent, longest fringe seta 4.7× as long as maximum discal width. Marginal vein with two long setae centrally, approximately equal in length. Stigmal vein moderately enlarged, with three sensilla apically.

Head and body uniformly very pale brown. **C3** darker than remainder of antenna (Figure 12g). Fore wing basally infuscate (cf Figure 13c).

*Male*: Characteristics as for female (Figure 13a–c) (except antenna and genitalia); although, metasoma darker than in female. Antenna as in Figure 12h, with a much shorter **C3** compared to the female.

*Material examined*. Holotype ♀ (deposited in AICF). **MALAYSIA**: Sabah (Borneo), Danum Valley, 05°01′ N 117°49′ E, 16.ix.2012, fogged tree, T. Cockerill, DNA: SAM12.

Paratypes: **MALAYSIA**: Sabah (Borneo), Maliau Basin Studies Centre, Belian Trail, 04°44′ N 116°58′ E, 20.ix.2012, screen-sweep, A. Polaszek NHM(E) 2010-21, DNA: SAM4 to SAM8 (4♂, 1♀, AICF, NHMUK).

*Species-group placement*: *M. ghesquierei*-group.

*Distribution*: Malaysia (Borneo, Sabah).

*DNA data*: CO1: four sequences; 28S: six sequences.

*Etymology*: Named for pitcher-plant (*Nepenthes*) botanist and wildlife photographer Chien C. Lee (Sarawak, Malaysian Borneo).

***Megaphragma cockerilli* Polaszek and Fusu sp. nov.** (Figure 13d–f)

urn:lsid:zoobank.org:act:09B45F53-180F-4204-9AC7-69DE9E352132

*Description*. *Female*: Antenna (Figure 13d) five-segmented (excluding anellus); pedicel as long as funicle; funicle 4× as long as wide; **C2** longer than **C1**. **C1** with two prominent dorsal **UST**, proximal **UST** almost as long as entire clava; **C2** with two **MT** and an **SB**, which is only slightly shorter than **C2**. **MPS** apparently absent.

Mid lobe of mesoscutum (Figure 13e) entirely with large, coarse reticulation; propodeum with a rhomboid, laterally arcuate central area, its hind margin truncate, with fine crenulae. **T1**–**T4** largely smooth, with scattered denticles and no setae laterally. **T5** and **T6** with a pair of long setae centrally. Ovipositor 1.1× as long as mesotibia. Mesotibia without spines basally; metafemur without spine; metatibia with a row of five spines within the distal inner half; a robust spine towards the apex of the outer surface. Fore wing (Figure 13f) 8.5× as long as maximum width, maximum distal width 1.4× maximum basal width; discal setae arranged in 3–4 rows, of 4–6 setae per row, longest fringe seta 5× as long as the maximum discal width. Marginal vein with two long setae centrally, of equal length. Stigmal vein with two sensilla apically.

Body largely dark brown, mesosoma paler laterally; antenna very dark brown. Fore wing infuscate basally.

*Male*: Unknown.

*Material examined*: Holotype ♀ (deposited in AICF): **MALAYSIA**: Sabah (Borneo), Danum Valley, 05°01′ N 117°49′ E, 16.ix.2012, fogged tree, T. Cockerill, DNA: SAM11.

*Species-group placement*: *polychaetum*-group. Very close to *M. polychaetum*, differing by the extremely elongate terminal sensillum basiconicum.

*Distribution*: Malaysia (Borneo, Sabah)

*DNA data*: CO1: one sequence; 28S: one sequence.

*Etymology*: Named for our colleague and friend, Dr Tim Cockerill, collector of this species (Falmouth University, UK).

**Figure 14:**
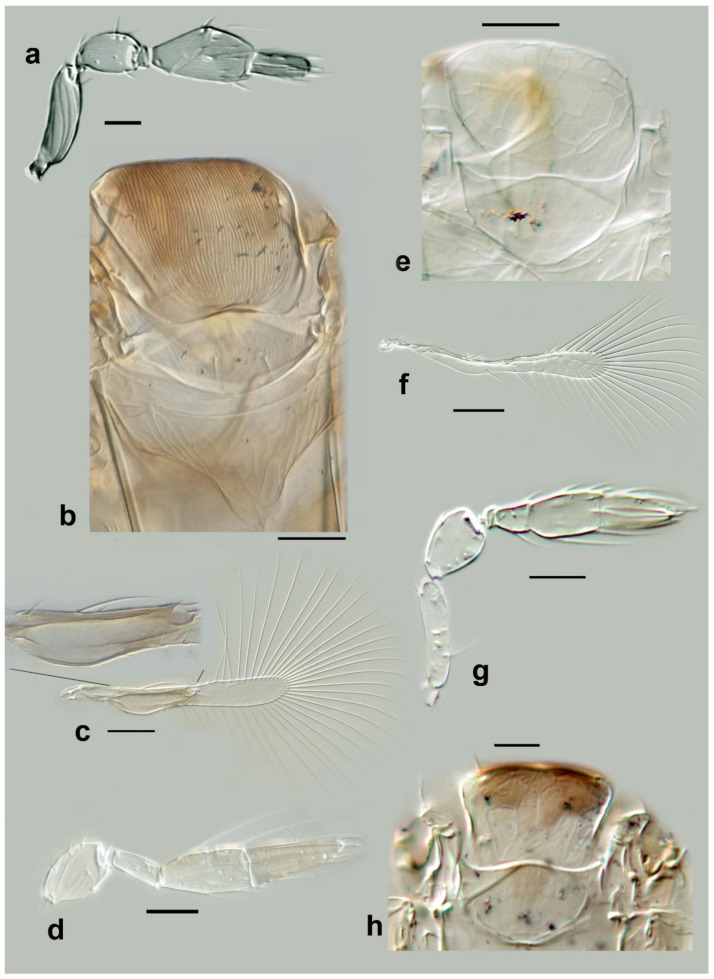
Photographs of *Megaphragma* species: (**a**) *M. digitatum*, female antenna (Holotype); (**b**) *M. digitatum*, female dorsal mesosoma (Holotype); (**c**) *M. digitatum*, female fore wing (Holotype); (**d**) *M. fanenitrakely*, female antenna (Holotype); (**e**) *M. fanenitrakely*, female mesosoma (Holotype); (**f**) *M. fanenitrakely*, female fore wing (Holotype); (**g**) *M. funiculatum*, female antenna (Holotype); (**h**) *M. funiculatum*, female mesosoma (Holotype). Scale bars 20 µm except 50 µm for c and f.

***Megaphragma digitatum* Polaszek and Fusu sp. nov.** (Figure 14a–c)

urn:lsid:zoobank.org:act:136D58D7-A1FC-4D46-8F63-A7780E4D0871

*Description. Female*: Antenna (Figure 14a) five-segmented (excluding anellus); funicle absent; hence, clava three-segmented, with **C1** and **C2** almost fused; **C1** with ≥4 **MT**, without **UST**; **C2** with 2 **UST** and abundant **MT**; **C3** with 2–3 **MPS**, **SB**, and **SS**.

Mid lobe of mesoscutum (Figure 14b) with longitudinal striate sculpture extending to scutellum; propodeum with central area extending posteriorly, crenulae present; **T1** with one elongate cell or groove laterally, 2–3× as long as wide; **T2**–**T4** without setae laterally; **T5** with long setae laterally. Ovipositor 1.5× as long as mesotibia. Mesotibia with one large spine basally; metafemur with spine. Fore wing (Figure 14c) 8.5× as long as maximum width, maximum distal width equal to maximum basal width; the disc with a single long seta;longest fringe seta 5× as long as maximum discal width. Marginal vein with two setae centrally; proximal seta 5–7× as long as distal seta, extending to the end of the marginal vein (in Figure 14c, the distal seta is barely visible in the space between the proximal one and the marginal vein). Stigmal vein moderately enlarged, with two sensilla apically.

Body largely brown, the following paler: legs except coxae and metafemora. Antenna with pedicel pale; scape and **C1**–**C3** darker. Fore wing strongly infuscate basally; stigmal and marginal vein brown; marginal vein very dark centrally.

*Male*: Largely as in female. **C1** and **C2** with scattered **SS**; **C2** with 2–3 **MT** apically; **C3** with long apical and ventral **UST**.

*Material examined*. Holotype ♀ (deposited in NHMUK). **COSTA RICA**: Puntarenas, Est. Biol. Monteverde, 10°19′ N 83°49′ W, 1540–1890 m, 26.ii.2007, J.S. Noyes BMNH(E) 2010-21, DNA: CRM2.1.

Paratypes: **COSTA RICA**: same data as holotype except DNA: CRM2.3, 2.6, 2.9, 2.12 (2♂, 2♀, AICF, MZUCR); Cartago, 12.5 km S Turrialba Rancho Naturalista, 1000 m, 9°50′’ N 83°34′’ W, 12-14.ii.2017, J.S. Noyes BMNH(E) 2017-39, DNA1681 (1♀, NHMUK). **ECUADOR**: Km 26.5 road Dura–Tambo, Estación Experimental Litoral Sur, INIAP, 21.xi.2017, ex *Chaetanaphothrips signipennis* on *Musa paradisiaca*, M. Arias col., DNA: ECU3 (3♀, 1♂ AICF, NHMUK, UCRC); same data except DNA: ECU4 (3♀ 2♂, AICF, NHMUK, UCRC); same data but ex *Frankliniella parvula*, DNA: ECU1 (2♀, NHMUK).

Non-type: same data as holotype (1♀, without wings, NHMUK).

*Species-group placement*: *ghesquierei*-group.

*Distribution*: Costa Rica, Ecuador.

*Hosts*: *Chaetanaphothrips signipennis* (Bagnall); *Frankliniella parvula* Hood.

*DNA data*: CO1: two sequences (Costa Rica); 28S: eight sequences (six Costa Rica, two Ecuador).

*Etymology*: The species name refers to the digitate **C3**.

***Megaphragma fanenitrakely* Polaszek and Fusu sp. nov.** (Figure 14d–f)

urn:lsid:zoobank.org:act:4DE1AD1D-C800-48C7-898B-24353F5355F0

*Description. Female*: Antenna (Figure 14d) five-segmented (excluding anellus), with pedicel slightly longer than funicle (12:8); clava two-segmented, **C1** with two **UST**; one **SB** at the apex of **C1** and **C2**; apex of **C2** (Figure 14d) also with two elongate **MPS** and a long **SB**.

Mesoscutum with mid lobe (Figure 14e) entirely with coarse, reticulate sculpture; metanotum and propodeum medially short. Metasoma with a row of microspines on each segment. **T1** without cells. Ovipositor 1.1× as long as mesotibia. Mesotibia without spines basally. Metafemur without prominent spine. Fore wing (Figure 14f) 9× as long as wide, maximum distal width 1.5× maximum basal width; the disc with 10 setae is irregularly arranged in 1–2 rows, and the longest fringe seta 6× maximum discal width. Marginal vein with two long setae centrally, of equal length. Stigmal vein moderately enlarged, with three sensilla apically.

Body largely pale brown, mesosoma paler laterally; antenna brown. Wings hyaline.

*Male*: Unknown.

*Material examined*. Holotype ♀ (deposited in NHMUK). **MADAGASCAR**: Nosy Komba, 13°27′45″ S 48°20′18″ E, 460 m, 22.vi.2015, screen-sweep, A. Polaszek col. BMNH(E)2015-122.

Paratypes: **MADAGASCAR**: Nosy Komba, closed canopy forest, 13°27′11″ S 48°20′4″ E, 170 m, 19.vi.2015, yellow pan trap, A. Polaszek col. BMNH(E)2015-122 (2♀, NHMUK).

*Species-group placement*: *longiciliatum*-group.

*Distribution*: Madagascar.

*DNA data*: no DNA sequences.

*Etymology*: A noun in apposition; “fanenitra kely” = “tiny wasp” (Malagasy).

**Figure 15:**
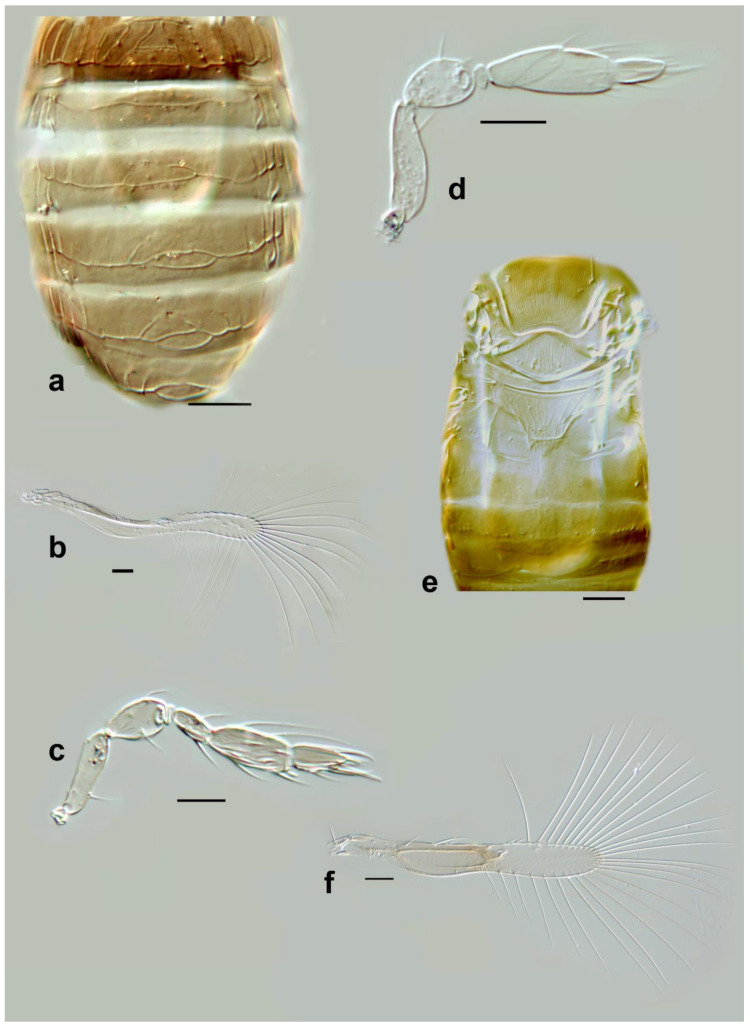
Photographs of *Megaphragma* species: (**a**) *M. funiculatum*, female metasoma (Holotype); (**b**) *M. funiculatum*, female fore wing (Holotype); (**c**) *M. giraulti*, female antenna (Holotype); (**d**) *M. hansoni*, female antenna (Holotype); (**e**) *M. hansoni*, female dorsal meso- and metasoma (Holotype); (**f**) *M. hansoni*, female fore wing (Holotype). Scale bars 20 µm.

***Megaphragma funiculatum* Fusu, Polaszek, and Viggiani sp. nov.** (Figure 14g,h and Figure 15a,b)

urn:lsid:zoobank.org:act:B6273DD4-14D8-4C91-9057-489883DA0DDE

*Description. Female*: Antenna (Figure 14g) five-segmented (excluding anellus), pedicel twice as long as funicle, the latter trapezoid, and slightly longer than wide; **C1** slightly shorter than **C2**; **C1** with 2 dorsal **UST**; **C2** with ≥3 **MPS** 1 **SB** and a short **SS**.

Mid lobe of mesoscutum smooth, with weakly impressed large subpolygonal sculpture (not visible in paratype which has been strongly macerated). Metanotum and propodeum relatively long centrally, each about half the length of scutellum. Propodeum with short central area, without crenulae. **T1** (Figure 15a) sculpture with cells converging centrally, lateral cells 2–3× as long as wide, without denticles (some denticles present on innermost cells). **T2**–**T4** without long setae laterally, each with similar sculpture comprising a central irregular oval cell and elongate lateral cells; those on **T3** and **T4** divided medially. Ovipositor 1.3× as long as mesotibia. Mesotibia with one large spine basally; metafemur without spine. Fore wing (Figure 15b) 10× as long as wide, maximum distal width 1.2× maximum basal width; the disc with a single irregular row of five setae; longest fringe seta 5.5× maximum discal width. Marginal vein with two long setae centrally.

Head, including antenna, pale; mesosoma largely pale, anterior half of mesoscutal mid lobe brown; metasoma entirely brown, **T1** darker than the remainder. Wings hyaline.

*Male*: Unknown.

*Material examined*. Holotype ♀ (deposited in NHMUK). **COSTA RICA**: Limón, Hitoy-Cerere Reserve, 100 m, 9°40′ N 83°02′ W, 24–26.ii.2008, J.S. Noyes col. NHM(E) 2010-21AQ, DNA: CRM 3.103.

Paratypes: same data as holotype, DNA: CRM 3.46 (1♀, AICF), CRM 3.100 (1♀, MZUCR).

*Species-group placement*: *mymaripenne*-group.

*Distribution*: Costa Rica.

*DNA data*: 28S: two sequences.

*Etymology*: Named for the distinctive funicle.

***Megaphragma giraulti* Viggiani, Fusu, and Polaszek sp. nov.** (Figure 4c–g, Figure 5a and Figure 15c)

urn:lsid:zoobank.org:act:9F02CA78-49F3-49D3-A4B8-DC4E3A3033EF

*Megaphragma* sp.: Huber and Noyes, 2013. *J. Hymenopt. Res.* 32: 37, Figs 49–51.

*Description. Female*: Antenna (Figure 4d and Figure 15c) five-segmented (excluding anellus), with pedicel slightly longer than funicle (12:8); clava two-segmented, **C1** 1.5× as **C2**, with ≥10 **MT** and 2 **UST**; one **SB** at the apex of **C1** and **C2**; apex of **C2** (Figure 4g) also with 2 elongate **MPS**, a long **SB** and **UST**.

Mid lobe of mesoscutum and scutellum without apparent sculpture; metanotum and propodeum medially short. Metasoma with a row of microspines on each segment. **T1** without cells (Figure 4f). Ovipositor 1.1× as long as mesotibia. Mesotibia without spines basally. Metafemur without prominent spine. Fore wing (Figure 5a) 9× as long as wide, maximum distal width 1.5× maximum basal width; disc with 10 setae irregularly arranged in 1–2 rows; fringe with longest seta 6× maximum discal width. Marginal vein with two long setae centrally, of equal length. Stigmal vein moderately enlarged, with three sensilla apically.

Body brown/yellow. Mesosoma largely pale, but mid lobe of mesoscutum brown anteriorly. Scape and pedicel pale, **C1**–**C3** brown. Fore wing slightly infuscate basally.

*Male*: As female, but antenna (Figure 4c) with **C1** approximately 2× **C2**. Metasoma (Figure 4e) with a row of microspines on each segment.

*Material examined*. Holotype ♀ (deposited in NHMUK). **COSTA RICA**: Puntarenas, Est. Biol. Monteverde, 10°19′ N 83°49′ W, 1540–1890 m, 26.ii.2007, J.S. Noyes BMNH(E) 2010-21, DNA: CRM2.4.

Paratypes: **COSTA RICA**: same data as holotype, DNA: CRM2.5 (1♀, AICF), CRM2.7, 2.8, 2.10, 2.11 (4♀, DACE, MZUCR, NHMUK); Limón, Hitoy-Cerere Reserve, 9°40′ N 83°02′ W, 100 m, 24–26.ii.2008, J.S. Noyes NHM(E) 2010-21AQ, DNA: CRM 3.23/B11, CRM 3.105, B9, E1 (2♀, 2♂, NHMUK); Cartago, 12.5 km S Turrialba, Rancho Naturalista, 1000 m, 9°50′’ N 83°34′’ W, 12–14.ii.2017, J.S. Noyes BMNH(E) 2017-39, DNA1683 (1♀, NHMUK); Heredia, La Selva Biol. Sta., 10°26′ N 84°01′ W, 75 m, 27–28.ii.2003, J. S. Noyes (1♂, DACE).

Non-types: **ARGENTINA**: Salta Prov. Orán, road to San Andres along Rio Blanca, 399 m, 23.09° S 63.37° W, 23.iii.2003, J. Munro 003-03-23-02 (1♀, UCRC). **USA**: Northampton, 7 km S Jackson, 23.ix–14.xi.1987, **MT**, Bald Cypress Swamp, BRC Hym Team (1♀, UCRC).

*Species-group placement*: *polychaetum*-group. The male antenna is very distinctive in the group, and *M. giraulti* male antenna agrees very well with several other species that definitely belong to the *polychaetum*-group (but without sequence data to back up this assertion).

*Distribution*: Argentina, Costa Rica, USA.

*DNA data*: CO1: four sequences; 28S: eight sequences (all Costa Rica).

*Etymology*: The species is named for A.A. Girault for his pioneering studies on the Trichogrammatidae.

***Megaphragma hansoni* Polaszek, Fusu, and Viggiani sp. nov.** (Figure 15d–f)

urn:lsid:zoobank.org:act:980B8787-BB40-4589-91D4-FB4262F4BA0F

*Description*. *Female*: Antenna (Figure 15d) five-segmented (excluding anellus); funicle absent; hence clava three-segmented, with **C1** and **C2** almost fused; **C1** with 1–2 **MT**; **C2** with some **MT** and 2 **UST**; **C3** with 2–3 **MT**, 2 **MPS**, and prominent **SB** and **SS**.

Mid lobe of mesoscutum (Figure 15e) with longitudinal striate sculpture extending to scutellum; propodeum (Figure 15e) with central area extended posteriorly, crenulae present; **T1** with elongate cells laterally, 2–3× as long as wide; **T2**–**T4** without setae laterally; **T5** with long setae laterally. Ovipositor 2× as long as mesotibia. Mesotibia with one large spine basally; metafemur with spine. Fore wing (Figure 15f) 7× as long as maximum width, maximum distal width equal to maximum basal width; disc with a single short seta; longest fringe seta 5× as long as maximum discal width. Marginal vein with one long seta centrally, extending almost to the end of the marginal vein; a minute additional seta next to it. Stigmal vein moderately enlarged, with four sensilla apically.

Body largely brown, the following paler: most of mesosoma except anterior half of mid lobe of mesoscutum, anterior half of **T1** and antenna. Fore wing slightly infuscate basally; stigmal and marginal vein pale brown.

*Male*: Largely as in female. **C1** and **C2** with scattered **SS**; **C2** with 2–3 **MT**, 1 apically; **C3** with long apical and ventral **UST**.

*Material examined*. Holotype ♀ (deposited in NHMUK). **COSTA RICA**: Limón, Hitoy-Cerere Reserve, 9°40′ N 83°02′ W, 100 m, 24–26.ii.2008, J.S. Noyes NHM(E) 2010-21AQ, DNA: CRM 3.101 (but no associated DNA sequence).

Paratypes: **COSTA RICA**: same data as holotype except DNA: CRM3.4, 3.17/B5, 3.40/D4, 3.97, 3.99, 3.104 (CRM3.40 lost after DNA extraction) (1♀, 4♂, AICF, NHMUK); Puntarenas, Est. Biol. La Gamba, 8°42′ N 83°12′ W, 150 m, 13–14.ii.2008, J.S. Noyes BMNH(E) 2010-21AQ, DNA: CRM1.1, 1.2, 1.4, 1.7, 1.12-1.19 (12♂, DACE, MZUCR, NHMUK, UCRC).

*Species-group placement*: *ghesquierei*-group.

*Distribution*: Costa Rica.

*DNA data*: 28S: seven sequences.

*Etymology*: Named for our colleague and co-author on this paper, Professor Paul Hanson, University of Costa Rica, San José.

***Megaphragma kinuthiae* Polaszek, Fusu, and Viggiani sp. nov.** (Figure 5b–d)

urn:lsid:zoobank.org:act:E6685BED-8C4E-46AB-9E70-9C0FBF6FCF6A

*Description. Female*: Antenna (Figure 5b) five-segmented (excluding anellus); pedicel as long as funicle; funicle 3× as long as wide; **C1** slightly longer or as long as **C2** with two dorsal **UST**; three elongate **MPS** extending beyond clava tip.

Head with toruli separated by about their own width. Mid lobe of mesoscutum smooth; propodeum with straight hind margin, without crenulae; **T1** non-reticulate; **T2**–**T4** with short setae laterally. Mesotibia with two large spines basally; metafemur with spine; metatibia with a row of fine, blunt setae extending almost their entire inner length, increasing abruptly in length distally. Fore wing (Figure 5d) about 9× as long as maximum width, maximum distal width 1.3× maximum basal width; discal setae arranged in two rows, each with 4–5 setae, longest fringe seta 10× as long as maximum discal width. Marginal vein with two long setae centrally. Stigmal vein with two sensilla apically. Ovipositor 1.1× as long as mesotibia.

Body entirely yellow, anterior mesosoma brown, posterior metasoma slightly darker. Wings hyaline.

*Male*: Unknown.

*Material examined*. Holotype ♀ (deposited in NHMUK). **KENYA**: Meru, vi. 1965, ID. No. 2851, CIE 233, BM 196. T. F. Crowe, ex tea leaves.

Paratypes: same data as holotype (6♀, NHMUK). All specimens are on the same slide; the holotype is circled in red.

*Species-group placement*: *polychaetum*-group. The species appears closest to *M. giraulti* based on morphology.

*Distribution*: Kenya.

*Host*: Not identified, but possibly *Scirtothrips dorsalis* (Hood), a species common on tea in Kenya.

*DNA data*: no DNA sequences.

*Etymology*: Named for our colleague and friend Dr Wanja Kinuthia, National Museums of Kenya, Nairobi.

**Figure 16:**
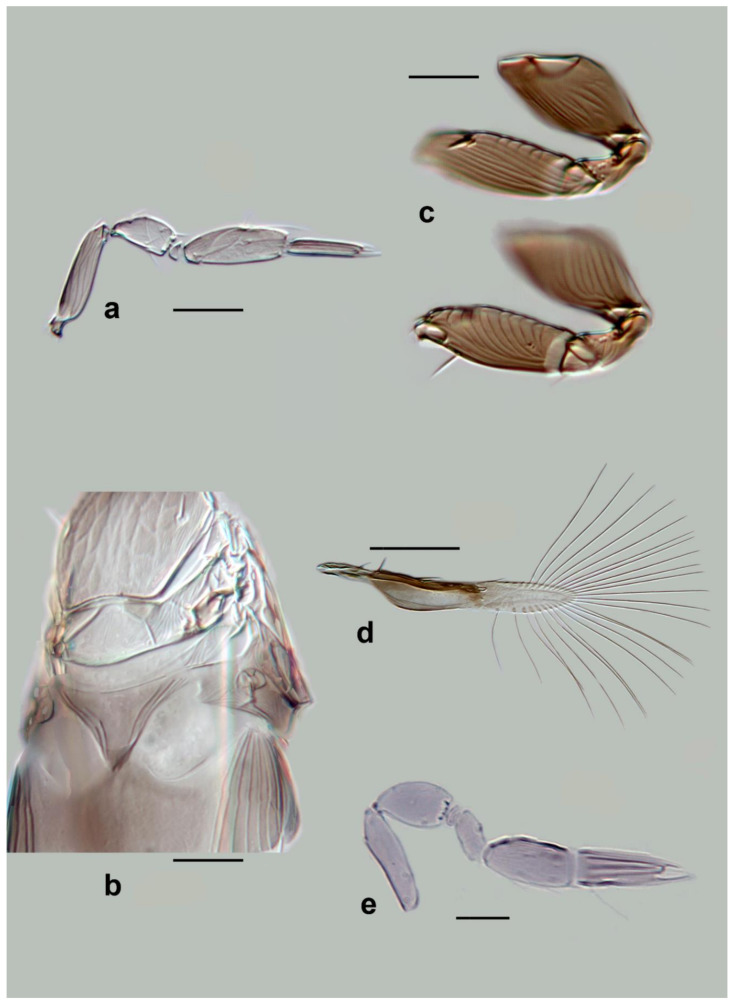
Photographs of *Megaphragma* species: (**a**) *M. liui*, female antenna; (**b**) *M. liui*, female dorsal meso- and metasoma (Holotype); (**c**) *M. liui*, female metacoxa and metafemur (Holotype); (**d**) *M. liui*, female fore wing (Holotype); (**e**) *M. longiciliatum*, female antenna (Holotype). Scale bars 20 µm except 50 µm for d.

***Megaphragma liui* Polaszek and Fusu sp. nov.** (Figure 16a–d)

urn:lsid:zoobank.org:act:35AD8001-C03B-4711-ADF5-22AFB681F184

*Description. Female*: Antenna (Figure 16a) five-segmented (excluding anellus), **C1** and **C2** strongly overlapping; **C3** elongate, more than half the length of **C1** and **C2**; 1 min **UST** on **C1**, two long **UST** on **C2**; three elongate **MPS** extending beyond clava tip.

Head with toruli very close together, separated by about one-third their own width. Mid lobe of mesoscutum (Figure 16b) with fine longitudinal striations, but also with distinct large reticulate cells; propodeum medially with strongly produced hind margin, with two crenulae (Figure 16b). **T1** smooth centrally, but with 8–10 elongate cells laterally (Figure 16b); **T2**–**T4** with short setae laterally, lateral cells present. Mesotibia without large spines basally, but a robust spine present at the apex of mesofemur; metafemur with spine; metatibia with a group of fine, sharp setae on inner surface apically. Metacoxa and metafemur (Figure 16c) with distinct longitudinal sculpture ventrally, contrasting with transverse sculpture dorsally. Fore wing (Figure 16d) 7× as long as maximum width, maximum distal width 1× maximum basal width; disc distally pointed, without setae (but one wing with a possible indication of a minute seta); longest fringe seta 4× as long as maximum discal width. Marginal vein with two setae centrally, the proximal one very robust, about 1.5× as long as distal. Stigmal vein with one elongate sensillum apically. Ovipositor 1.9× as long as mesotibia.

Body entirely brown, mesosoma pale posteriorly, **T1** with pale areas laterally. **C1** very dark, pedicel paler than the remainder of the antenna. Fore wing strongly infuscate basally. Legs dark, tarsi pale.

*Male*: Unknown.

*Material examined*. Holotype ♀ (deposited in UCRC). **BRUNEI**: Temburong Dist., Bukit Patoi trail, 41–290 m, 4°45′21″ N 115°10′30″ E, 4 July 2010, swp dipterocarp forest, J. Mottern M10-065, DNA1656.

*Species-group placement*: *ghesquierei*-group. In the concatenated and partitioned analysis, this species is not included in the group (Figure 2b), though in an unpartitioned analysis with a simple substitution model it is one of the most basal species of the *ghesquierei*-group (Figure 2a). It is also retrieved as part of the group in the tree based on the 28S sequences alone, where it is sister to *M. rivelloi* but on a very long branch (Supplementary Figure S3). Hence, morphology, and partly molecular analyses, indicate that our inclusion of the species is correct.

*Distribution*: Brunei.

*DNA data*: CO1: one sequence; 28S: one sequence.

*Etymology*: Named for our colleague and friend Prof. Shu-sheng Liu, Zhejiang University, Hangzhou, China.

**Figure 17:**
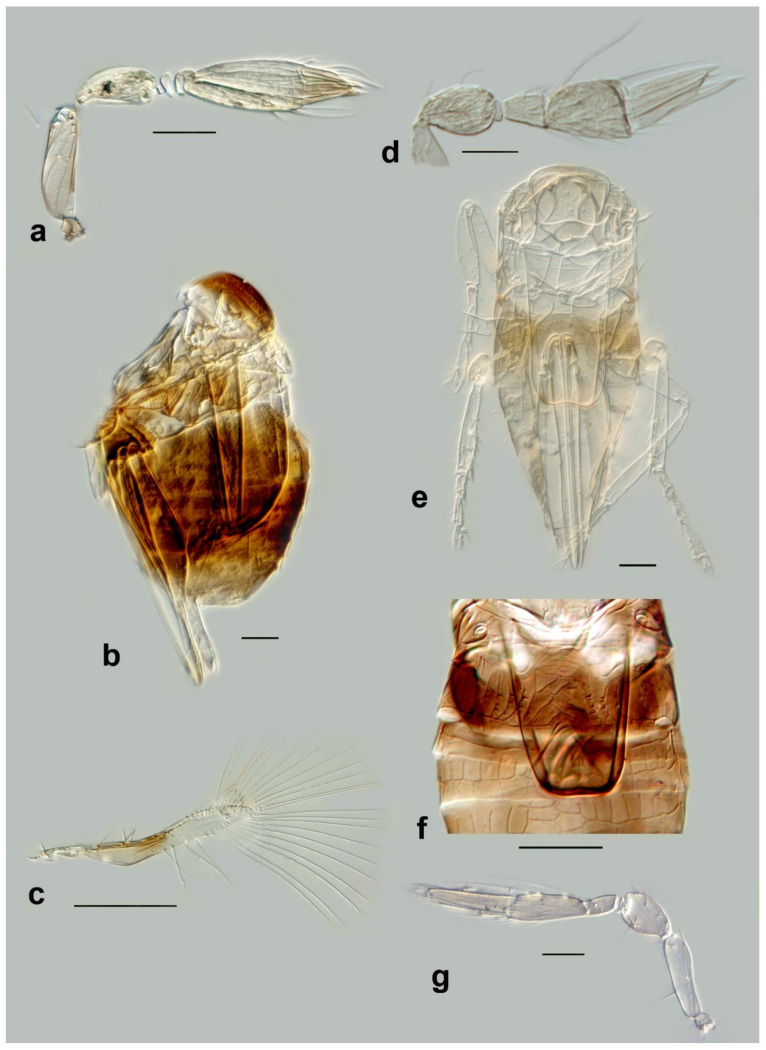
Photographs of *Megaphragma* species: (**a**) *M. momookherjeeae*, female antenna (Holotype); (**b**) *M. momookherjeeae*, female lateral meso-and metasoma (Holotype); (**c**) *M. momookherjeeae*, female fore wing (Holotype); (**d**) *M. mymaripenne*, female antenna (Holotype); (**e**) *M. nowickii*, female dorsal meso- and metasoma (Holotype); (**f**) *M. nowickii*, female dorsal base of metasoma (Holotype); (**g**) *M. nowickii*, female antenna (Holotype). Scale bars 20 µm except 100 µm for c.

***Megaphragma momookherjeeae* Polaszek and Fusu sp. nov.** (Figure 17a–c)

urn:lsid:zoobank.org:act:CC6535BA-271C-44CB-9697-CC048ED070E0

*Description. Female*: Antenna (Figure 17a) five-segmented (excluding anellus); transverse funicle present, clava two-segmented; **C1** longer than **C2**; **C1** with ≥5 **MT**, 2 **UST**, and with fine, longitudinal striation; **C2** with basal **SS**, 2 **UST**, and ≥4 **MPS**.

Mid lobe of mesoscutum smooth; propodeum without distinct central area. **T1**–**T4** largely smooth, **T2**–**T4** with long setae laterally. Ovipositor exserted, exceptionally long for the genus, more than 3× as long as mesotibia (Figure 17b). Mesotibia with a very robust spine basally, 0.4× tibial length; metafemur without spine; metatibia with a row of about 17 spines along almost the entire inner length, and 4 robust spines toward the apex of the outer surface. Fore wing (Figure 17c) 9× as long as maximum width;maximum distal width 1× maximum basal width; disc with a single, minute seta; longest fringe seta 6.5× as long as maximum discal width. Marginal vein apparently with three long setae centrally, of equal length. Stigmal vein with three sensilla apically.

Body largely dark brown; scutellum, propodeum, and lateral mesosoma paler (Figure 17b); antenna pale brown, **C2** darker. Fore wing basally infuscate.

*Male*: Unknown.

*Material examined*. Holotype ♀ (deposited in NHMUK). **COSTA RICA**: Cartago, 12.5 km S. Turrialba, Rancho Naturalista, 1000 m, 9°50′’ N 83°34′’ W, 12–14.ii.2017, J.S. Noyes BMNH(E) 2017-39, DNA1680.

*Species-group placement*: *M. antecessor*-group. Resembling also the *ghesquierei*-group in some features (e.g., fore wing with one seta), but clearly not clustering with that group in any molecular analyses. It is recovered as sister to *M. antecessor*, and the two clustered together as basal to all species-groups except the *ghesquierei*-group (partitioned analysis), or as the most basal species group of *Megaphragma* (unpartitioned analysis).

*Distribution*: Costa Rica.

*DNA data*: CO1: one sequence.

*Etymology*: Named for Mo Mookherjee, a friend of the first author (AP).

***Megaphragma nowickii* Polaszek, Fusu, and Viggiani sp. nov.** (Figure 7a–f and Figure 17e–g)

urn:lsid:zoobank.org:act:126687C2-A7AE-4FC2-810E-11DE6E6D6784

*Description. Female*: Antenna (Figure 7c and Figure 17g) five-segmented (excluding anellus); pedicel twice as long as funicle; funicle twice as long as wide; **C1** slightly shorter or as long as **C2**; **C1** with ≥7 **MT**; 2 **UST**; 3 elongate **MPS** extending beyond clava apex.

Mid lobe of mesoscutum smooth, anteriorly with subpolygonal sculpture (cf Figure 7b). Metanotum and propodeum narrow centrally, the latter without an extension or crenulae. **T1** sculpture (Figure 7a and Figure 17f) with cells converging centrally, lateral cells 3× as long as wide, each with 3–5 inward-pointing denticles; **T2**–**T4** without long setae laterally, all with coarse reticulate sculpture becoming lateral distally. Ovipositor 1.7× as long as mesotibia. Mesotibia with two large spines basally; metafemur with spine; metatibiae with a row of fine, blunt setae extending almost their entire inner length, increasing abruptly in length distally. Fore wing (Figure 7e) 9.5× as long as wide, maximum distal width 1.1× maximum basal width; disc with a single row of six setae; longest fringe seta 4.5× maximum discal width. Marginal vein with two long setae centrally.

Body entirely brown, metasoma slightly darker posteriorly. Pleural parts of mesosoma and hind legs except for tarsi lighter. Wings hyaline.

*Male*: Largely as in female. Antenna with **C1** slightly longer than **C2**; **T1** (Figure 7b) with an incomplete pattern of cells. Aedeagus as in Figure 7f.

*Material examined*. Holotype ♀ (deposited in NHMUK). **D. R. CONGO**: Province Orientale, Yangambi Biosphere Reserve, 15.v.2012, N 0°48.837′ E 24°30.287′, screen sweep, primary forest, A. Polaszek col, BMNH(E) 2012-88, DNA: COM1.16.

Paratypes: **D. R. CONGO**: same data as holotype except DNA: COM1.1–1.15, 1.17–1.23, 1.26, 1.27 (2♀, 22♂, AICF, DACE, IITA, NHMUK). **UGANDA**: Mabira Forest, N0°23′22″ E33°00′22″, 1250 m, 1.iii.2015, A. Polaszek, screen sweep, DNA1132/F11 (1♀, NHMUK); Mabira Forest, N0°23′22″ E33°00′22″, 1250 m, 1.iii.2015 A. Polaszek, screen sweep, DNA1116–1118, 1120–1124 (1♀, 7♂, NHMUK).

Non-types: **BENIN**: Dept. Zou, Zogbodomey, Massi, 18.xii.1989, ex egg *Megalurothrips sjostedti* on *Pueraria*, M. Tamo col. 275 (1♂, 1♀, NHMUK, IITA).

*Species-group placement*: *mymaripenne*-group.

*Distribution*: Benin, D. R. Congo, Uganda.

*Host*: *Megalurothrips sjostedti* (Trybom).

*DNA data*: CO1: 25 sequences from D. R. Congo; 28S: 30 sequences from 2 countries: D. R. Congo (21), Uganda (9).

*Etymology*: Named for S. Nowicki for his outstanding contribution to the knowledge of the Trichogrammatidae.

**Figure 18:**
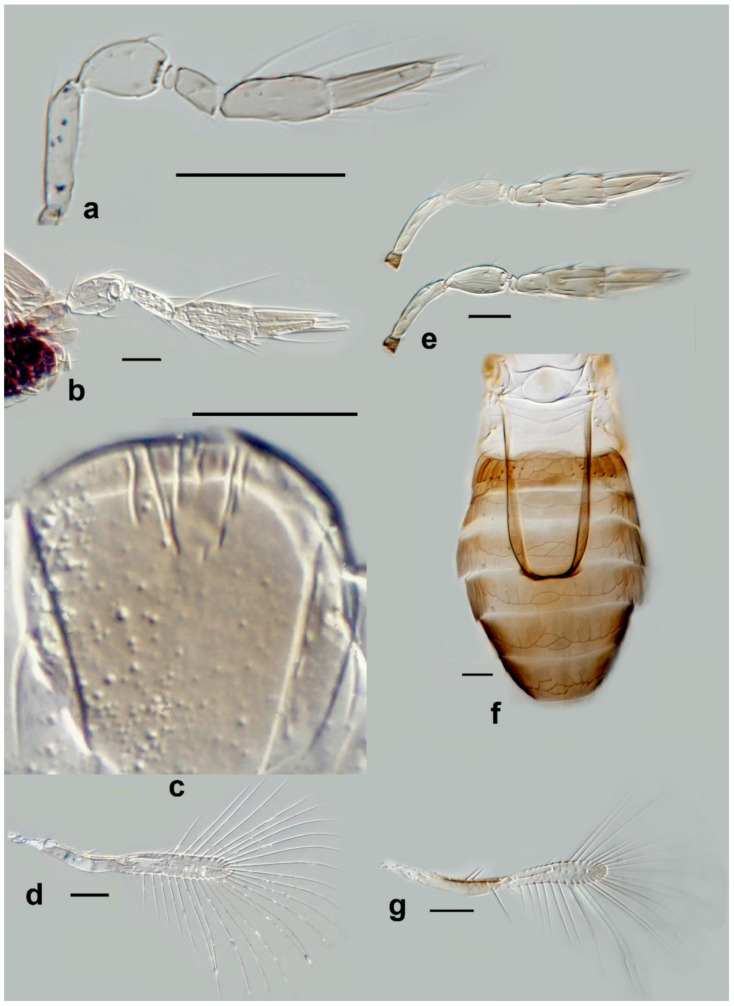
Photographs of *Megaphragma* species: (**a**) *M. noyesi*, female antenna (Holotype); (**b**) *M. pintoi*, female antenna (Holotype); (**c**) *M. pintoi*, female mesoscutum (Holotype); (**d**) *M. pintoi*, female fore wing (Holotype); (**e**) *M. polilovi*, female antenna (Holotype); (**f**) *M. polilovi*, female dorsal mesosoma (part) and metasoma (Holotype); (**g**) *M. polilovi*, female fore wing (Holotype). Scale bars 20 µm except 50 µm for a, d, and g.

***Megaphragma noyesi* Polaszek and Fusu sp. nov.** (Figure 18a)

urn:lsid:zoobank.org:act:63048F49-FCC2-49FF-9E2C-C0552BC1FBF4

*Description*. *Female*: Antenna (Figure 18a) five-segmented (excluding anellus); pedicel almost twice as long as funicle; funicle slightly longer than wide; **C1** shorter than **C2**; **C1** with two dorsal **UST**; **C2** with three elongate **MPS** extending beyond clava apex.

Mid lobe of mesoscutum and scutellum smooth. Metanotum and propodeum narrow centrally, the latter without an extension or crenulae.

Mesotibia without large spines basally; metafemur without spine; metatibia with a row of robust spines extending along the inner surface of distal half.

Fore wing 9× as long as wide, maximum distal width 1.3× maximum basal width; disc with a single row of six setae, and longest fringe seta 5× maximum discal width. Marginal vein with two long setae centrally, subequal in length (cf Figure 18g). **T1** sculpture with cells converging centrally, six lateral cells present, 2–3× as long as wide, each with 1–3 inward-pointing denticles; **T2**–**T4** with lateral cells indicated, with lateral setae not detected (cf Figure 18f). Ovipositor as long as mesotibia. Body largely brown, mesosoma largely pale. **T1** very dark brown in contrast to rest of body. Legs pale. Wings hyaline.

*Male*: Largely as in female. Antenna with **C1** longer than **C2**; **T1** with an incomplete pattern of cells. Genitalia tubular as in other species, 3× as long as wide.

*Material examined*. Holotype ♀ (deposited in NHMUK). **UK**: England, Surrey, Coulsdon Common, Happy Valley, 51°17′ N 0°07′ W, 168 m, viii.2013, J.S. Noyes BMNH(E) 2013-, DNA: UKM14.

Paratypes: **UK**: same data as holotype except DNA: UKM8–13 (UKM9 has DNA sequences but was lost during extraction) (4♀, 1♂, AICF, DACE, NHMUK); East Sussex, Brede High Wood, TQ79432018/19, 30.viii.2019–20.ix.2019, D. Binns col., DNA1612–1619 (1612, 1614 and 1618 have DNA sequences but were lost during extraction) (10♀, 3♂, NHMUK, UCRC). **HUNGARY**: Őrség Nemzeti Park, Barkás Lake, 46°52′ N 16°26′ E, 268 m, 28.vi.2010, J.S. Noyes screen sweeping, BMNH(Ent) 2010-63, DNA: HUM2 (1♀, NHMUK) (HUM3 has DNA sequences but was lost during extraction), HUM5 (1♀, AICF); 4–5 km SW Kőszeg, Meszes Völgy, 47°22′ N 16°31′ E, 431 m, 26.vi.2010, screen sweeping, J.S. Noyes BMNH(Ent) 2010-63, DNA: HUM6 (1♂, AICF); Őrség National Park, Lugosi Valley, 46°54′ N 16° 27′ E, 231 m, 28.vi.2010, J.S. Noyes, DNA: HUM 8–14, BMNH(Ent) 2010-63 (3♀ 4♂, NHMUK).

Non-type: **CZECH REPUBLIC**: Moravia, Vranov, River Dyje, ss riparian forest, 13.viii.1991, L. Masner (1♀, UCRC).

*Species-group placement*: *mymaripenne*-group.

*Distribution*: UK (England); Czech Republic, Hungary.

*DNA data*: CO1: 21 sequences from 2 countries; 28S: 23 sequences from 2 countries: Hungary, UK.

*Etymology*: Named for Dr John Noyes of the Natural History Museum, London, for his outstanding contribution to our knowledge of Chalcidoidea. As a chalcid collector, John is unmatched so far, and a major proportion of the material in this study was collected by him.

***Megaphragma pintoi* Viggiani sp. nov.** (Figure 18b–d)

urn:lsid:zoobank.org:act:9C3F4C6B-6290-4DB5-B4E2-6080C11F6C91

*Description*. *Female*: Antenna (Figure 18b) five-segmented (excluding anellus); pedicel slightly longer than funicle; funicle 3× as long as wide; **C1** slightly longer or as long as **C2**; **C1** with two **UST**; three **MPS** extending beyond **C2** apex.

Mid lobe of mesoscutum (Figure 18c) smooth, with 5–6 deep striae anteriorly; propodeum with straight hind margin, without crenulae. **T1** non-reticulate; **T2**–**T4** with short setae laterally. Ovipositor 1.1× as long as the mesotibia. Metafemur without spine but a prominent seta present. Fore wing (Figure 18d) 10× as long as maximum width, and maximum distal width 1.4× maximum basal width; discal setae arranged in 2–3 rows, each with 3–5 setae, longest fringe seta 5.5× as long as maximum discal width. Marginal vein with two long setae centrally. Stigmal vein with two sensilla apically.

Body entirely pale brown (eyes deep purple); legs and antenna pale. Wings hyaline.

*Male*: Unknown.

*Material examined*. Holotype ♀ (deposited in PFRNZ). **NEW ZEALAND**: Auckland, Mt Albert, Science Center, 17.v.1997, ex *Heliothrips haemorrhoidalis* ex acmena leaves, P. Stevens.

Non-type: **COLOMBIA**: Supata, 5°04′07″ N 74°16′48″ W, 1800 m, 31.12.2018, sweep, A.A. Polilov col. (1♀, AICF).

*Species-group placement*: *polychaetum*-group.

*Distribution*: Colombia, New Zealand.

*DNA data*: no DNA sequences.

*Etymology*: Named for Emeritus Professor John Pinto, formerly of University of California, Riverside, in recognition of his monumental contribution to our understanding of Trichogrammatidae.

*Comments*: Known so far from only two specimens to date; this species has an apparently extraordinary distribution, being known from Colombia and New Zealand. It seems very likely that it will turn up elsewhere and is probably another cosmopolitan *Megaphragma* species.

**Figure 19:**
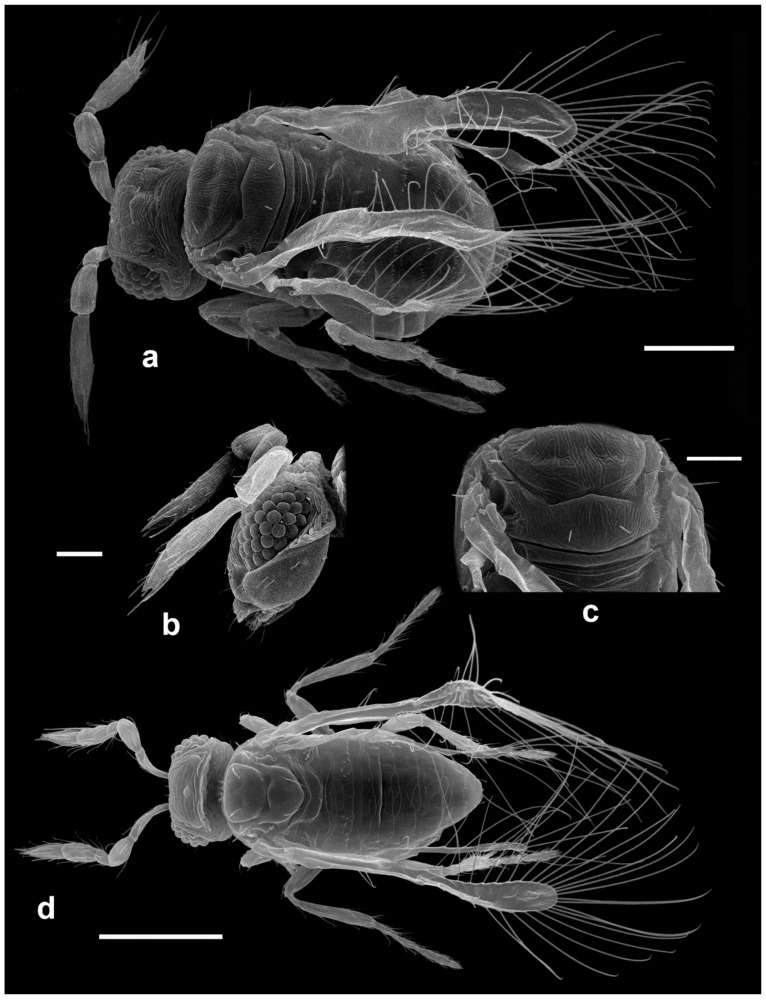
SEM micrographs of *Megaphragma* species (non-types): (**a**) *M. caribea*, male habitus; (**b**) *M. caribea*, male head and antennae; (**c**) *M. caribea*, male mesoscutum; (**d**) *M. polilovi*, female dorsal habitus. Scale bars 50 µm for a, 20 µm for b and c, and 100 µm for d.

**Figure 20:**
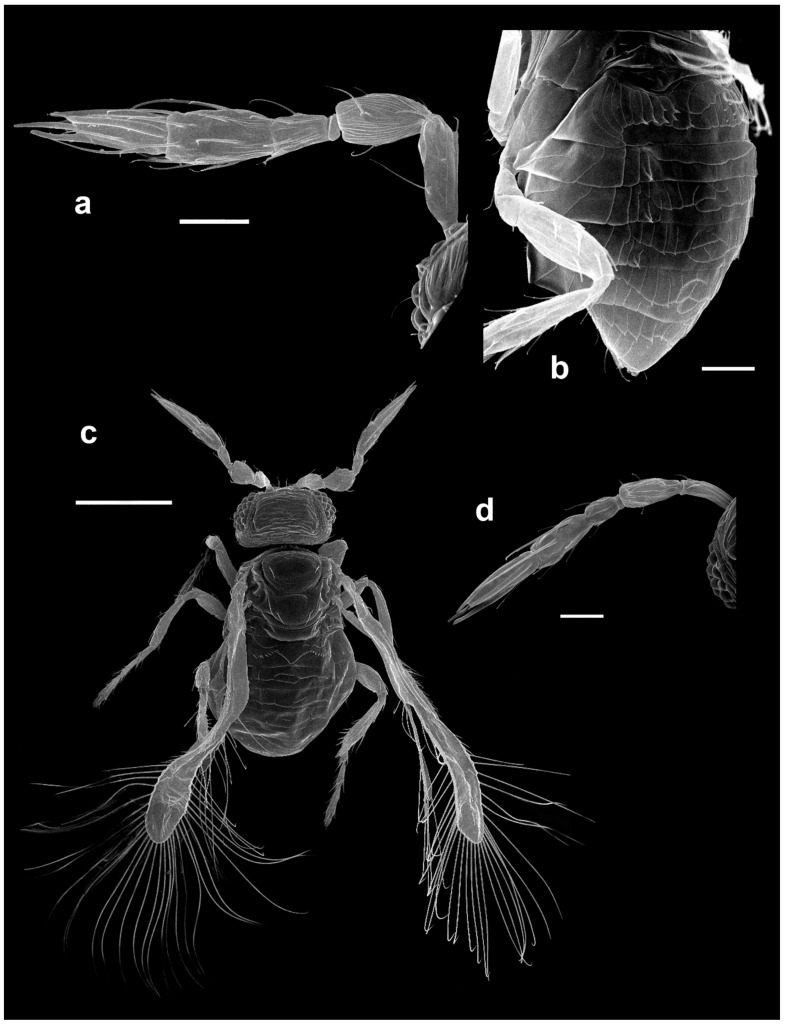
SEM micrographs of *Megaphragma* species (non-types): (**a**) *M. polilovi*, female antenna; (**b**) *M. polilovi*, female metasoma; (**c**) *M. viggianii*, female habitus; (**d**) *M. viggianii*, female antenna. Scale bars 20 µm for a, b and d, and 100 µm for c.

***Megaphragma polilovi* Polaszek, Fusu, and Viggiani sp. nov.** (Figure 18e–g, Figure 19d and Figure 20a,b)

urn:lsid:zoobank.org:act:7A812908-FC9A-4F84-9A1C-1699DC922132

*Megaphragma mymaripenne*: Viggiani and Bernardo, 1998. *Boll. Zool. agr. Bach. Ser. II* 29: 51–55; Bernardo and Viggiani, 2003. *Boll. Lab. Entomol. agr. Filippo Silvestri* 58 [2002]: 77–85; Polilov, 2012. *Arthropod Struct. Dev.* 41(1): 29–34; Makarova et al., 2015. *Arthropod Struct. Dev.* 44(1): 21–32; Polilov, 2016. *At the Size Limit—Effects of Miniaturization in Insects*; Polilov, 2017. *PLoS ONE* 12(5): e0175566; Diakova et al., 2018. *PeerJ* 6: e6005 (misidentifications).

*Description*. *Female*: Antenna (Figure 18e and Figure 20a) five-segmented (excluding anellus); pedicel almost twice as long as funicle; funicle slightly longer than wide, with 3 **MT** and 1 ASC; **C1** shorter than **C2**; **C1** with 17 **MT**, **SS**, and 2 **UST**; **C2** with **SB**, 2 **MT**, **SS**, and 4 elongate **MPS** extending beyond clava apex.

Mid lobe of mesoscutum and scutellum smooth (Figure 18f and Figure 19d). Propodeum with a distinct central area with lateral boundaries in line with those of mesoscutum and scutellum (Figure 19d); two lateral lobes present behind propodeum central area (Figure 19d); propodeum without crenulae. **T1** sculpture (Figure 18f and Figure 20b) with cells converging centrally, about six lateral cells, 2× as long as wide, mesal cells each with 2–3 inward-pointing denticles. **T2**–**T4** with 2–3 lateral cells, with short setae laterally. Ovipositor 1.3× as long as mesotibia. Mesotibia with two large spines basally; metafemur without spine; metatibia with a row of fine, blunt setae extending almost its entire inner length, increasing abruptly in length distally. Fore wing (Figure 18g) 9.5× as long as wide, maximum distal width 1.4× maximum basal width; disc with a single row of six setae, longest seta of fringe 5.8× maximum disc width. Marginal vein with two long setae centrally.

Antenna (Figure 18e) with radicle brown, very dark compared to the remainder of the antenna; remainder of body largely brown, mesosoma largely pale, mid lobe of mesoscutum brown anteriorly, an indistinct brown spot on the scutellum. Legs pale. Wings hyaline.

*Male*: Largely as in female. Antenna with **C1** slightly longer than **C2**; **T1** with an incomplete pattern of cells. Genitalia tubular as in other species, 3× as long as wide.

*Material examined*. Holotype ♀ (deposited in DACE). **ITALY**: Vietri sul Mare, Benincasa, 40°40′ N 14°44′ E, 17.vii.2013, G. Viggiani ex *Heliothrips haemorrhoidalis* on *Viburnum tinus*, DNA: ITM9.

Paratypes: **ITALY**: same data as holotype, DNA: ITM8, 11, 12, 14 (4♀, AICF, DACE, NHMUK).

*Species group placement*: *mymaripenne*-group.

*Distribution*: Italy.

*Host*: *Heliothrips haemorrhoidalis*. Males are very rare, and reproduction is normally thelytokous [14] (as *M. mymaripenne*).

*DNA data*: CO1: five sequences; 28S: five sequences; all from Italy.

*Etymology*: Named for our colleague Alexey Polilov, co-author of this paper, for his outstanding contribution to our knowledge of the Trichogrammatidae and miniaturization in insects.

*Comments*: Found in Italy at the same locality and on the same host as *M. viggianii*.

**Figure 21:**
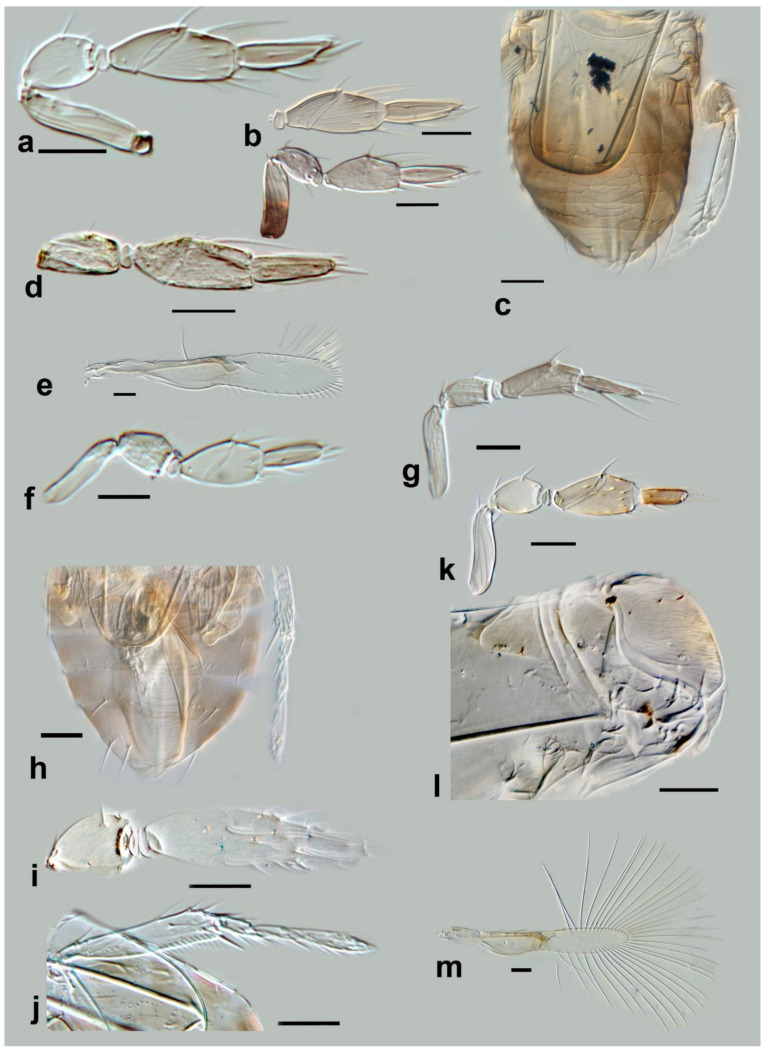
Photographs of *Megaphragma* species: (**a**) *M. rivelloi*, female antenna (Holotype); (**b**) *M. striatum*, female antenna (MXM1 above and Paratype below); (**c**) *M. striatum*, female metasoma (MXM1); (**d**) *M. tamoi*, female antenna (Holotype); (**e**) *M. tamoi*, female fore wing (Holotype); (**f**) *M. tridens*, female antenna (Holotype); (**g**) *M. tridens*, male antenna (Paratype); (**h**) *M. tridens*, female metasoma (Holotype); (**i**) *M. uniclavum*, female antenna (Holotype); (**j**) *M. uniclavum*, female metatibia (Holotype); (**k**) *M. vanlentereni*, female antenna (Holotype); (**l**) *M. vanlentereni*, female mesosoma (Holotype); (**m**) *M. vanlentereni*, female fore wing (Holotype). Scale bars 20 µm.

***Megaphragma rivelloi* Viggiani sp. nov.** (Figure 9a–d and Figure 21a)

urn:lsid:zoobank.org:act:4436D2E2-590F-4329-A65B-AB562F0E04ED

*Megaphragma* sp. Viggiani, 2002. *Boll. Zool. Agr. Bach. Ser. II* 34: 449–450.

*Description*. *Female*: Antenna (Figure 9a and Figure 21a) five-segmented (excluding anellus), pedicel approximately two-thirds the length of **C1** and **C2**; **C3** one-quarter shorter than **C2**; **C2** with a single **UST**; **C3** with **SB**, two **MPS**, **MT**, and apical **SB**.

Mid lobe of mesoscutum and scutellum with longitudinally striate sculpture; propodeum (Figure 9b) with a large subtriangular and crenulated central area. Metasomal tergites without reticulation but with transverse striations, laterally with a row of 2–7 microspines. Ovipositor approximately 1.3× as long as mesotibia. Fore wing (Figure 9c) 6–7× as long as wide; disc without setae; longest fringe seta 4–4.5× as long as the maximum discal width.

Body yellow with brown mostly on mesosoma; fore wing infuscate behind the venation.

*Male*: Largely as in female except for genitalia.

*Material examined*. Holotype ♀ (deposited in DACE): **ITALY**: Basilicata, Rivello (PZ), vii.2002, yellow sticky traps in a vineyard.

Paratypes: **ITALY**: same data as holotype (19♀, DACE, NHMUK). **INDIA**: UP, New Delhi, IARI; 220 m 28°37′51″ N, 77°09′50″ E, xi.5.2003, pan trap, J. Heraty col. H03-106 (1♀, UCRC). **INDONESIA**: W Java, Gunung Halimun NP, Tea-Forest Junction, 1066 m, 6°41′07″ S 106°31′16″ E, screen-sweep, 17.ix.2015, A. Polaszek, DNA1148 (1♀, NHMUK). **JAPAN**: Tokyo area, ?1984, Takagi col., ex *Scirtothrips dorsalis* on tea, A. Loomans leg. (5♀, AICF, NHMUK). **UK**: Surrey, Coulsdon Common, 25.viii.2002, J.S. Noyes, screen sweep (1♂, NHMUK). **VIETNAM**: Cat Tien NP, sweeping, N11°24′45″ E107°25′23″, 21.xi.2018, leg. A.A. Polilov, DNA1687 (1♀, AICF); Cat Tien NP, sweeping, N11°24′45″ E107°25′23″, 25.xi.2016, leg. A.A. Polilov (3♀, 2♂, AICF, UCRC).

Non-type: **CHINA**: 1♂ misidentified as *M. deflectum*, Wuyishan, Fujian, 19.x.1987 Wang Jiashe col. (FAU).

*Species group placement*: *ghesquierei*-group.

*Distribution*: China, India, Indonesia, Italy, Japan, UK, Vietnam.

*Host*: *Scirtothrips dorsalis* Hood.

*DNA data*: 28S: 1 sequence (Vietnam).

*Etymology*: Named for the ancient village of Rivello in Italy, where this extremely widespread species was first discovered. As noted above under *M. deflectum*, one paratype (male “allotype”) of that species is in fact *M. rivelloi*.

***Megaphragma tamoi* Polaszek, Fusu, and Viggiani sp. nov.** (Figure 21d,e)

urn:lsid:zoobank.org:act:8F82A348-AFC5-4618-A0C8-416867AC501C

*Description*. *Female*: Antenna (Figure 21d) five-segmented (excluding anellus); funicle absent; hence, clava three-segmented, with **C1** and **C2** almost fused; **C1** without **UST**; **C2** with one elongate **UST**, reaching more than half the length of **C3**; **C3** with **MPS**, **SB**, and **SS**.

Mid lobe of mesoscutum with longitudinal striate sculpture extending to scutellum (cf Figure 14b); propodeum with central area extended posteriorly, crenulae present (cf Figure 15e). **T2**–**T4** without setae laterally. Ovipositor 1.7× as long as mesotibia. Mesotibia with one large spine basally; metafemur with spine (cf Figure 16c, upper). Fore wing 6× as long as the maximum width, maximum distal width 1.2× maximum basal width; disc with a single long seta (Figure 21e), longest fringe seta 3× maximum discal width. Marginal vein with two setae centrally, equal in length, extending to the end of the marginal vein. Stigmal vein moderately enlarged, with two sensilla apically.

Largely brown, the following paler: legs except coxae and metafemur. Pedicel pale; scape, **C1**–**C3** darker. Fore wing strongly infuscate basally; stigmal and marginal vein brown; marginal vein very dark centrally.

*Male*: Largely as in female. **C1** and **C2** with scattered **SS**; **C2** with 2–3 **MT** apically; **C3** with long apical and ventral **UST**. **C3** is darker than preceding segments.

*Material examined*. Holotype ♀ (deposited NHMUK). **BENIN**: Agbotagon, 6°49′ N 2°12′ E, 22.iv.1990, M. Tamo 351.

Paratypes: **BENIN**: same data as holotype (8♀, DACE, IITA); Mono Province, 7.x.1988, M. Tamo, D-Vac on cowpea *Vigna unguiculata* (1♀, NHMUK); Mono Province, 25.ii.1988, M. Tamo, emergence cage *Megalurothrips sjostedti* on *Vigna unguiculata* (1♀, IITA); Dept Zou, Zogbodomey, 22.i.1990, ex egg *Megalurothrips sjostedti*, M. Tamo col. 275, 342 (2♀, IITA); Cotonou, IITA Station, 16.i.1990, ex egg *Megalurothrips sjostedti* on *Pueraria*, M. Tamo col. 336 (1♀, NHMUK).

*Species group placement*: *ghesquierei*-group.

*Distribution*: Benin.

*Host*: *Megalurothrips sjostedti*.

*DNA data*: no DNA sequences.

*Etymology*: Named for our colleague and friend Manu Tamo (IITA, Benin), collector of many *Megaphragma* specimens.

***Megaphragma tridens* Fusu and Polaszek sp. nov.** (Figure 21f–h)

urn:lsid:zoobank.org:act:C77515FA-E1EA-4C74-B7DA-CE244A2BD05F

*Description*. *Female*: Antenna (Figure 21f) five-segmented (excluding anellus); funicle absent; hence, clava three-segmented, with **C1** and **C2** almost fused; **C1** apparently without **UST**; **C2** with 1 prominent **UST**, abundant **MT**, and 1 apical **MPS**; **C3** with **MT**, 2-3 **UST**, **SB**, and **SS**.

Mid lobe of mesoscutum with longitudinal striate sculpture (cf Figure 14b); propodeum with central area extended posteriorly, crenulae absent (cf Figure 21l). **T1** with elongate cells laterally, 2–3× as long as wide (cf Figure 12b); **T2**–**T4** with a short, robust seta near lateral margin; **T5** centrally with subparallel striations and with long setae laterally (Figure 21h). Ovipositor 1.7× as long as the mesotibia. Mesotibia with one large spine basally; metafemur with spine. Fore wing 8.5× as long as the maximum width, maximum distal width equal to maximum basal width; disc with a single long seta (cf Figure 12c), longest fringe seta 3.5× as long as maximum discal width. Marginal vein with one long seta centrally, extending to the end of the marginal vein. Stigmal vein moderately enlarged, with four sensilla apically.

Body largely brown, the following paler: legs except coxae and metafemur. Pedicel pale; scape, **C1**–**C3** darker. Fore wing slightly infuscate basally; stigmal and marginal vein brown.

*Male*: Largely as in female. Antennal clava (Figure 21g) with **C1** and **C2** with scattered **SS**; **C2** with 2-3 **MT** apically; **C3** with long apical and ventral **UST**.

*Material examined*. Holotype ♀ (deposited in NHMUK). **COSTA RICA**: Puntarenas, La Gamba Biol. Sta., 150 m, 8°42′ N 83°12′ W, 13–14.ii.2008, J.S. Noyes BMNH(E) 2010-21AQ, DNA: CRM1.10.

Paratypes: **COSTA RICA**: same data as holotype, DNA: CRM1.5, 1.8 (1♀, 1♂, AICF, MZUCR); Limón, Hitoy-Cerere Reserve, 9°40′ N 83°02′ W, 100 m, 24–26.ii.2008, J.S. Noyes NHM(E) 2010-21AQ, DNA: CRM 3.41, 3.102 (1♀ 1♂, AICF, NHMUK).

*Species-group placement*: *ghesquierei*-group.

*Distribution*: Costa Rica.

*DNA data*: no DNA sequences.

*Etymology*: From the Latin word *tridens*, in reference to the three apical sensilla on **C3**, resembling a longer central and two shorter lateral teeth of a trident (from Latin *tri*=three and *dens*=tooth). Noun in apposition.

***Megaphragma uniclavum* Polaszek and Fusu sp. nov.** (Figure 21i,j)

urn:lsid:zoobank.org:act:1F77A0D1-B9C0-46D2-A553-3176AAF76708

*Description*. *Female*: Antenna (Figure 21i) four-segmented (excluding anellus); transverse funicle present; clava one-segmented (unique so far for the genus, though *antecessor* approaches this condition); 2 **UST**; 3–4 long **MPS** extending beyond half the clava.

Head with few features discernible due to mounting position, and extensively obscured by eye pigment. Mid lobe of mesoscutum with fine longitudinal striation (cf Figure 14b); vertical/ventral anterior mid lobe of mesoscutum with coarse, reticulate sculpture (cf Figure 3g); propodeum elongate centrally, longitudinally striate with three large crenulae. **T2**–**T4** with very long setae laterally, each longer than its tergum; **T3** and **T4** with large irregular cells laterally. Mesotibia with two large spines basally; metafemur with spine; metatibia with a row of fine, elongate setae extending almost their entire inner length (Figure 21j). Fore wing 5× as long as maximum width; longest fringe seta 6.7× as long as maximum discal width, maximum distal width versus maximum basal width is unclear; disc (cf Figure 11b) with single short seta. Other details of disc and venation are unclear. Stigmal vein with a row of three sensilla apically. Ovipositor 2× as long as the mesotibia.

Body uniformly pale. Distal metasoma darker. Wings hyaline.

*Male*: Unknown.

*Material examined*. Holotype ♀ (deposited in NHMUK). **COSTA RICA**: Heredia, La Selva BS., 10°26′ N 84°01′ W, 75 m, 28–29.ii.2008, J.S. Noyes BMNH(E) 2010-21 AQ.

*Species-group placement*: *antecessor*-group. *Megaphragma uniclavum* is so far unique for the genus having a single claval segment. Despite the very unusual fore-wing structure, it is clearly affiliated with *M. antecessor* (similar mesoscutal sculpture and row of setae on metatibia).

*Distribution*: Costa Rica.

*DNA data*: no DNA sequences.

*Etymology*: This species is so far the only *Megaphragma* known with a single claval segment; hence, the name *uniclavum*. Noun in apposition.

***Megaphragma vanlentereni* Polaszek and Fusu sp. nov.** (Figure 21k–m)

urn:lsid:zoobank.org:act:E76B71C9-D7A7-4005-A7E1-66EC26A146BB

*Description*. *Female*: Antenna (Figure 21k) five-segmented (excluding anellus); funicle absent; hence, clava three-segmented, with **C1** and **C2** almost fused; **C1** with 2 **UST**; **C3** with three **MPS** extending beyond apex of clava.

Mid lobe of mesoscutum (Figure 21l) with irregular longitudinal striate sculpture; propodeum (Figure 21l) elongate, curved centrally and posteriorly; crenulae present. **T1** without cells; **T2**–**T4** without setae laterally. Ovipositor 1.7× as long as mesotibia. Mesotibia with two large spines basally. Fore wing (Figure 21m)6.5× as long as maximum width, maximum distal width 1.1× maximum basal width; disc with one short seta, longest fringe seta 4× as long as maximum discal width. Marginal vein with two long setae centrally, of equal length. Stigmal vein moderately enlarged, with three sensilla apically.

Body largely pale with dorsal mesosoma, including propodeum, pale brown. Scape and pedicel pale, **C1** and **C2** brown, **C3** very dark brown in contrast. Fore wing infuscate basally; stigmal and marginal vein distally brown.

*Male*: Unknown.

*Material examined*: Holotype ♀ (deposited in NHMUK). **MALAYSIA**, Sabah, Maliau Basin Studies Centre, Knowledge Trail, 04°44′ N 116°58′ E, 22.ix.2012, A. Polaszek, screen-sweep, NHM(E) 2010-21, DNA: SAM3.

*Species group placement*: *ghesquierei*-group.

*Distribution*: Malaysia (Borneo, Sabah).

*DNA data*: CO1: one sequence; 28S: one sequence (both Malaysia, Sabah).

*Etymology*: Named for our colleague and friend Joop van Lenteren, a pioneer of biocontrol, especially in greenhouses.

**Figure 22:**
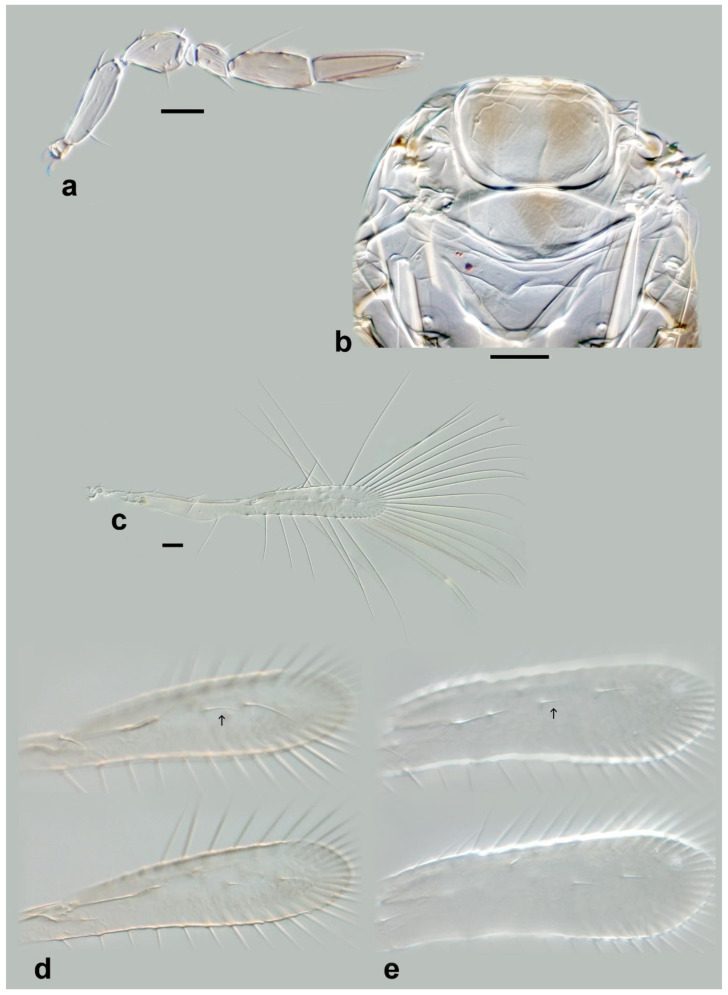
Photographs of *Megaphragma* species: (**a**) *M. viggianii*, female antenna (Holotype); (**b**) *M. viggianii*, female mesosoma (Holotype); (**c**) *M. viggianii*, female fore wing (Holotype); (**d**) *M. viggianii*, female ventral (above) and dorsal (below) wing surface, (Holotype); (**e**) *M. priesneri*, female ventral (above) and dorsal (below) wing surface (Neotype). Arrows point at the penultimate seta on ventral surface. Scale bars 20 µm.

***Megaphragma viggianii* Fusu, Polaszek, and Polilov sp. nov.** (Figure 20c,d and Figure 22a–d)

urn:lsid:zoobank.org:act:05F51567-04A0-45D8-91F7-787A041BEB08

*Megaphragma amalphitanum*: Nedoluzhko et al. 2016. *Mitochondrial DNA Part A* 27(6): 4526–4527; Polilov, 2016. *At the Size Limit—Effects of Miniaturization in Insects*; Nedoluzhko et al., 2017. *Genom. Data* 11: 87–88; Polilov, 2017. *PLoS ONE* 12(5): e0175566; Prokhortchouk et al., 2017. *Mosc. Univ. Biol. Sci. Bull.* 72(1): 30–32; Diakova et al., 2018. *PeerJ* 6: e6005; Sharko et al., 2019. *PLoS ONE* 14(12): e0226485; Polilov et al., 2021. *Sci. Rep.* 11(1): 4717; Boudinot et al., 2020. *J. Zool.* 313(2): 99–113; Diakova and Polilov, 2021. *J. Hymenopt. Res.* 84: 69–73 (misidentifications).

*Description*. *Female*: Antenna (Figure 20d and Figure 22a) five-segmented (excluding anellus); pedicel 2× as long as funicle; funicle slightly longer than wide; **C2** longer than **C1**; **C1** with 1 ASC, 2 **MT**; **C2** with 13 **MT**, 2 **UST**; **C3** with 2 **MPS**, 1 **SS**, and 3 **UST** extending beyond clava tip.

Mid lobe of mesoscutum (Figure 22b) posteriorly smooth, anteriorly with large, coarse reticulation; propodeum with broad, truncate hind margin, with two widely separated lobes distally and laterally, without crenulae. **T1** with a central “V” composed of minute denticles, a row of coarser denticles laterally (Figure 20c), and one elongate cell laterally, about 2× as long as wide. Metasoma dorsally with rows of denticles laterally on **T2** and **T3**; **T2**–**T4** with moderately long setae laterally. Ovipositor as long as the mesotibia.

Mesotibia without spines basally, a single seta present; metafemur without spine but with three robust setae; metatibia with a row of five or six fine setae extending along half its inner length. Fore wing (Figure 22c) 6× as long as maximum width; maximum distal width 1.5× maximum basal width and more than 2× width measured at the apex of the marginal vein; longest fringe seta 4× as long as the maximum discal width; disc with setae in one or two rows of 4–6 setae, and setae on ventral surface long, the penultimate one reaching to the base of the distal (Figure 22d). Marginal vein with two setae centrally, the proximal approximately 2× the length of the distal. Stigmal vein has four sensilla apically.

Head and metasoma dark brown; central/posterior mesoscutal mid lobe and lateral scutellum paler. Remainder of body, including legs, pale. Flagellum darker than remainder; wings hyaline.

*Male*: Antenna with pedicel almost as long as scape; funicle with two small setae; clava two-segmented; **C1** >2× **C2**; **C1** with three setae; **C2** with three flagelliform setae; two multiporous plate sensilla and a short terminal process present. Metasoma dorsally with rows of denticles laterally on **T1**–**T3**. One individual with funicle apparently fused with **C1**.

*Material examined*: Holotype ♀ (deposited in DACE). **ITALY**: Naples, Massa Lubrense, S. Agata sui Due Golfi, 24.x.2012, 40°36′ N 14°20′ E, 114 m, ex *Heliothrips haemorrhoidalis* in leaves of *Viburnum tinus* (G. Viggiani), DNA: ITM1.

Paratypes: **ITALY**: same data as holotype except DNA: ITM2 (1♂), ITM3 (1♂), ITM4 (1♂), ITM5 (1♂), ITM6 (1♀), ITM7 (1♀) (AICF, NHMUK); same data but without DNA extraction codes (2♀ NHMUK); Vietri sul mare, Benincasa, 40°40′ N 44°20′ E, 17.vii.2013, ex *Heliothrips haemorrhoidalis* on *Viburnum tinus* (G. Viggiani), DNA: ITM13 (1♀, NHMUK); Liguria, Santa Margherita, iv.2015, A. Polilov, ex *Heliothrips haemorrhoidalis* on *Viburnum tinus*, DNA1095, DNA1096 (2♀, NHMUK). **GREECE**: Koutsoupia, 39.81N 22.80E, 22.viii.2016, Leg. A.A. Polilov, ex eggs *Heliothrips haemorrhoidalis* on *Arbutus* sp. (2♀, 1♂, AICF).

*Species-group placement*: *longiciliatum*-group.

*Distribution*: Greece, Italy.

*Host*: *Heliothrips haemorrhoidalis*.

*DNA data*: CO1: eight sequences; 28S: eight sequences.

*Etymology*: Named for Professor Gennaro Viggiani, specialist of microhymenoptera and biocontrol, and instigator and co-author of this paper.

*Comments*: Found in Italy at the same locality and on the same host as *M. polilovi*. According to the CO1 sequence of the mitochondrial genome deposited on GenBank by Nedoluzhko et al. [23], their species is not *M*. *amalphitanum* (=*M. longiciliatum*) but *M. viggianii*. Our molecular analysis retrieves the two as sister species. Both have the mid lobe of the mesoscutum reticulate anteriorly, and long lateral setae on **T2**–**T4**, though these are longer in the former. In *M. longiciliatum* **T2**–**T4** have very long setae, at least as long as the tergite (Figure 5h), whereas in *M. viggianii* **T2**–**T4** have shorter setae, much shorter than the tergite (cf Figure 8f).

**Key to species of *Megaphragma***

**1**
Fore wing with a single discal seta (Figure 9h and Figure 10c) or without any discal setae (Figure 3f and Figure 4a)……………………………**2**
**-**
Fore wing with at least one line of 3 or more setae (Figure 3c and Figure 5d)…………………………………………………………………………**17**
**2**
Fore wing without discal seta (Figure 3f)…………….………………………………….……………………………………………………………………**3**
**-**
Fore wing with one discal seta (Figure 9h)……………………………………….…….……………………………………………………………………**6**
**3**
Mid lobe of mesoscutum entirely with regular longitudinally striate sculpture (Figure 3d)…………………………………………………………**4**
**-**
Mid lobe of mesoscutum with reticulate sculpture anteriorly (Figure 3g), or with irregular longitudinal sculpture (Figure 13b)………………**5**
**4**
Female: ovipositor more than 1.5× as long as mesotibia. Male: **C3** with terminal sensillum less than 2× length **C3**……………………***deflectum***
**-**
Female: ovipositor less than 1.5× as long as mesotibia. Male: **C3** with terminal sensillum more than 2× length **C3**…………………………***rivelloi***
**5**
Mid lobe of mesoscutum with reticulate sculpture anteriorly (Figure 3g)…………………………………………………………………***ghesquierei***
**-**
Mid lobe of mesoscutum with irregular longitudinal sculpture (Figure 13b)…………………………………………………………………***chienleei***
**6**
Fore wing with discal seta short, ≤distance between 2 proximal wing fringe setae (Figure 11b)……………………………………………………**7**
**-**
Fore wing with discal seta long, ≥distance between 3 proximal wing fringe setae (Figure 10c)……………………………………………………**12**
**7**
Female: ovipositor strongly exserted, more than 3× mesotibia (Figure 17b)…………………………………………………………***momookherjeeae***
**-**
Ovipositor slightly or not exserted, less than 2.5× mesotibia……………………………………………………………………………………………**8**
**8**
Clava 1-segmented (Figure 21i). Marginal vein with both central setae minute……………………………………………………………***uniclavum***
**-**
Clava 2- or 3-segmented (if 2-segmented, **C1** partially fused with **C2**). Marginal vein with at least one of the central setae elongate…………**9**
**9**
Marginal vein with 2 central setae, the distal one minute, less than 0.2× as long as the proximal seta (Figure 15f)………………………***hansoni***
**-**
Marginal vein with 2 central setae, either subequal in length (Figure 11b), or the distal one about 0.5× as long as the proximal seta (Figure 16d).……………………………………………………………………………………………………………………………………………………………**10**
**10**
Funicle present, transverse (Figure 11a); clava 2-segmented…………………………………………………………………………………***antecessor***
**-**
Funicle absent; clava 3-segmented…………………………………………………………………………………………………………………………**11**
**11**
Mid lobe of mesoscutum entirely with evident regular longitudinally striate sculpture while reticulate cells hardly visible (Figure 21l)…………………………………………………………………………………………………………………………………………………***vanlentereni***
**-**
Mid lobe of mesoscutum entirely with fine regular longitudinally striate sculpture, surface divided into obvious reticulate cells (Figure 16b)……………………………………………………………………………………………………………………………………………………………***liui***
**12**
Marginal vein with one central seta (Figure 12c)…………………………………………………………………………………………………………**13**
**-**
Marginal vein with two central setae………………………………………………………………………………………………………………………**15**
**13**
**T2**–**T4** of metasoma each with a robust seta near lateral margin; **T5** centrally with subparallel striations (Figure 21h). Male: **C3** elongate, length more than 3× basal width, with very long sensilla (Figure 21g)…………………………………………………………………………………………***tridens***
**-**
**T2**–**T4** of metasoma without a seta near lateral margin; **T5** centrally with reticulate sculpture (Figure 12b and Figure 21c). Male: **C3** elongate or short, with less prominent sensilla (Figure 10b)……………………………………………………………………………………………………………**14**
**14**
**T2**–**T5** each with rows of prominent denticles laterally; **T3** and **T4** centrally with reticulate sculpture (Figure 21c). Female: **C3** long (Figure 21b). Male: **C3** long, length more than 2× basal width (Figure 10b)…………………………………………………………………………………***striatum***
**-**
**T2**–**T5** with denticles reduced to faint protrusions, especially on **T3** and **T4**; **T3** and **T4** centrally with sculpture consisting of faint striae (Figure 12b). Female: **C3** short (Figure 12a). Male: **C3** short, length less than 2× basal width……………………………………………………………***breviclavum***
**15**
Clava 2-segmented………………………………………………………………………………………………………………………………***stenopterum***
**-**
Clava 3-segmented……………………………………………………………………………………………………………………………………………**16**
**16**
Marginal vein proximal central seta 2–3× length of distal central seta (Figure 21e)………………………………………………………………***tamoi***
**-**
Marginal vein proximal central seta about 7× longer than distal seta (Figure 14c)……………………………………………………………***digitatum***
**17**
Antenna 4-segmented (excluding anellus)…………………………………………………………………………………………………………………**18**
**-**
Antenna 5-segmented (excluding anellus)…………………………………………………………………………………………………………………**19**
**18**
Female: **C1** with 2 very short **UST**, much shorter than **C1** (Figure 6a). Male: **C1** 2× as long as maximum width …………………***macrostigmum***
**-**
Female: **C1** with 2 long **UST**, each as long as **C1** (Figure 3a). Male: **C1** 3× as long as maximum width………………………………………***caribea***
**19**
**T1** with characteristic sculpture consisting of distinct cells with denticulate margins (Figure 1a, Figure 17f and Figure 18f)…………………**20**
**-**
**T1** various, but not as above; elongate cells may be present on sides of **T1** (Figure 12b and Figure 16b)…………………………………………**24**
**20**
Female: **T2** sculpture with many closed cells (Figure 17f); ovipositor 1.7× mesotibia…………………………………………………………***nowickii***
**-**
Female: **T2** sculpture without closed cells (occasionally a single cell in noyesi); ovipositor 1.3× mesotibia or shorter…………………………**21**
**21**
Mid lobe of mesoscutum reticulate with comparatively large cells (Figure 14h)…………………………………………………………***funiculatum***
**-**
Mid lobe of mesoscutum smooth, or appearing longitudinally striate…………………………………………………………………………………**22**
**22**
Female: **C1** trapezoid in lateral view, length 1.5× maximum width or less. Male: funicle trapezoid, slightly longer than wide………………………………………………………………………………………………………………………………………………***mymaripenne***
**-**
Female: **C1** parallel sided and elongate, 2× as long as wide or more. Male: funicle elongate, approximately 2× as long as wide (male unknown in *M. polilovi*)……………………………………………………………………………………………………………………………………………………………**23**
**23**
Scape 4× maximum width; radicle concolorous with scape, both pale (Figure 18a); ovipositor length 1× mesotibia……………………………***noyesi***
**-**
Scape 6× maximum width; radicle darker compared to scape (Figure 18e); ovipositor length 1.3× mesotibia…………………………………***polilovi***
**24**
Mid lobe of mesoscutum coarsely reticulate over most of its surface (Figure 13e and Figure 14e)………………………………………**25**
**-**
Mid lobe of mesoscutum reticulate only anteriorly (Figure 3g), or sculpture different (smooth Figure 4f, striate Figure 3d)………………………**27**
**25**
Fore wing with base hyaline (not infuscate; Figure 14f)………………………………………………………………………………………***fanenitrakely***
**-**
Fore wing with base infuscate (Figure 13f)……………………………………………………………………………………………………………………**26**
**26**
**C2** with apical **SB** only slightly shorter than **C2** (Figure 13d) (male unknown)…………………………………………………………………***cockerilli***
**-**
**C2** with apical **SB** less than ½ length of **C2** (Figure 8a)…………………………………………………………………………………………***polychaetum***
**27**
**T2**–**T4** with very long setae laterally (Figure 5h), at least as long as the tergite…………………………………………………………………………**28**
**-**
**T2**–**T4** with short setae laterally (Figure 8f), much shorter than the tergite………………………………………………………………………………**29**
**28**
**T1** with long lateral setae. Female: funicle with long, robust **UST** (Figure 15c)……………………………………………………………………***giraulti***
**-**
**T1** without long lateral setae (Figure 5h). Female: funicle without long, robust ventral **UST** (Figure 5e and Figure 16e)………………***longiciliatum***
**29**
Mid lobe of mesoscutum reticulate anteriorly (Figure 8f and Figure 22b)…………………………………………………………………………………**30**
**-**
Mid lobe of mesoscutum with different sculpture (Figure 18c), or smooth………………………………………………………………………………**31**
**30**
Fore wing with maximum distal width <2× width measured at apex of marginal vein (Figure 8g); setae on ventral fore wing disc short, penultimate one not reaching to the base of the distal (Figure 22e)…………………………………………………………………………………………………***priesneri***
**-**
Fore wing with maximum distal width >2× width measured at apex of marginal vein (Figure 22c); setae on ventral fore wing disc long, penultimate one reaching to the base of the distal (Figure 22d)……………………………………………………………………………………………………***viggianii***
**31**
Mid lobe of mesoscutum smooth. Female: proximal **UST** attached close to the mid point of **C1**, shorter than **C1** (Figure 5b)……………***kinuthiae***
**-**
Mid lobe of mesoscutum with anterior striae (Figure 18c). Female: proximal **UST** attached close to the base of **C1**, longer than **C1** (Figure 18b)……………………………………………………………………………………………………………………………………………………………***pintoi***


## 4. Discussion

The present study is the culmination of more than 10 years of intensive collecting and examination of several thousand *Megaphragma* specimens from all over the world, including type material of all but one of the previously described species. Without the molecular dimension, our conclusions would have been very different. For example, the separation of *M. noyesi* and *M. polilovi* from *M. mymaripenne* would not have been possible, and these species have been confused in the past. Within the *ghesquierei*-group, morphological differences between species that are very distinct based on DNA, are completely undetectable in many cases. Some of this is no doubt due to the limitations of light microscopy, even when using techniques such as Nomarski differential interference contrast (DIC), coupled with focus stacking. Morphological evolution at species level is apparent for several structures, most notably the antenna, but also the setae and spines on the middle and hind legs, the fore wing, the structure of the propodeum, and that of metasomal terga showing variation in microsculpture and chaetotaxy (of diagnostic value). Species can be relatively easily grouped based on features of these morphological characters, and the *ghesquierei*-group, in particular, can be defined based on features of three characters: fusion of the funicle with the clava, development of the central propodeum, and metafemoral spine. The *mymaripenne*-group is less easily defined, with the loss of the otherwise characteristic sculpture of **T1** having occurred in the *longiciliatum* subgroup. The shape of the fore wing, and its discal and marginal ciliation appear to be critical in reflecting species evolution in this genus. Future studies should carefully assess the setation of the upper and underside of the fore wing, something that is very difficult once the specimen has been slide-mounted. No doubt future studies, including more scanning electron microscope imaging, will reveal additional patterns of morphological variation in the genus.

Perhaps the most surprising discovery of this study is the extraordinary distribution of some species. *Megaphragma longiciliatum*, under our new and broader definition, is found from Southeast Asia to Northwest Europe, as well as in North America, the Congo, and the Middle East. In this respect, as well as in terms of their physical size, the *Megaphragma* species parallel some Protozoa. The phrase “everything is everywhere, but the environment selects” [69], originally applied to Protozoa, certainly seems to apply to several *Megaphragma* species. Previous theories attempting to explain ubiquitous distributions of particular species of organisms have attributed this to their large population sizes, rather than to any inherent properties of such groups [70]. This argument appears to be so entirely back to front (i.e., “some species are cosmopolitan because they have huge populations”) that it can be easily dismissed. It is precisely the inherent property of minuteness, among other attributes discussed below, that is the main reason for these species having cosmopolitan distributions. Minuteness is directly related to dispersive ability, which can be largely passive for minute organisms; although, *Megaphragma* are known to be good at directional flight [28]. Our study, despite being very patchy in terms of the sample sizes of most species, suggests a mixed pattern of dispersal and distribution, with ubiquitous species as well as apparently endemic ones, as shown previously for about 200 Protozoa species [71]. Minuteness also directly affects the relationship between the species and its immediate microhabitat. We can assume that for an organism whose adults are around one-quarter of a millimeter, and whose developmental stages are entirely within a closed environment (the thrips egg), the macroecological factors of climate and temperature are less important, at least for some species. Hence, e.g., *M. rivelloi* appears to be as suited to the humid rainforests of SE Asia as it is to the much drier countryside of Southern England, and the same must be true for other species. Humidity, and especially avoidance of desiccation, are critical for the survival of minute terrestrial organisms, and *Megaphragma* species are known to have very thin cuticle [18] (see also Supplementary Figure S1). Even if air masses could transport minute hymenopterans quickly across the globe, desiccation would be a major impediment [72]. However, given that for most of their adult life *Megaphragma* species are likely to be in close proximity to living plant tissue, and hence access to moisture, it is probable that these immediate microclimatic conditions override the macroecological conditions already mentioned, and hence some of the remarkable latitudinal distributions of, e.g., *M. longiciliatum*, *M. pintoi,* and *M. rivelloi*. As well as both direct and passive colonization of new geographical areas, human movement of plant material containing thrips eggs, and *Megaphragma* life stages is inevitable, and has certainly contributed to a large extent to the cosmopolitan distribution of some species. Adaptation to, and dependence on, microhabitat conditions, especially humidity, have undoubtedly also contributed to their human-mediated distribution through the movement of plant material. Another explanation for species of egg parasitoids having extremely wide distributions has been their assumed defencelessness, and the relative uniformity of their hosts [73]. Whilst the former appears to be generally true, the latter clearly is not. Parasitoids that parasitize eggs are in some cases able to additionally parasitize Lepidoptera larvae, braconid cocoons, and even act as hyperparasitoids of other egg parasitoids. This extreme range of hosts has been reliably documented in *Centrodora darwini* (Girault) [74]. Other egg parasitoids appear to be extremely specific in which species they will either attack and/or develop successfully on, and this is particularly true of many species in the hyperdiverse genus *Telenomus* (e.g., [75]). Thus, a huge range of levels of host species specificity exists across egg parasitoids. In several taxa, eggs are entirely free of any parasitoids (aphids, whiteflies, and scale insects), while their (relatively) close relatives (leafhoppers and their relatives) are very heavily parasitized [76]. Clearly, the ability to evade or resist being parasitized at the egg stage is highly heterogeneous across the insects. In the case of *Megaphragma,* it is difficult to tell how host specific they are; in the few cases where there are rearing records from more than one host, they belong to different genera of the same family. Most likely as in other groups of parasitoid wasps, members of *Megaphragma* are a mix of generalists and specialist species [77].

As stated above, in several lineages *Megaphragma* has diversified to produce numerous cryptic species, many of which appear indistinguishable morphologically, at least using the techniques employed in this study. All future studies of these and similar organisms must rely to an extent on DNA data, and it may be that species will be described solely on differences in DNA where these differences can be demonstrated to be reasonably accurate proxies for biological species distinctions.

## Data Availability

All sequences were deposited in GenBank. Voucher specimens are deposited in the collections listed under Specimens and depository abbreviations. All data and materials will be publicly available.

## Supplementary Materials

The following supporting information can be downloaded at: https://www.mdpi.com/article/10.3390/insects13060561/s1, Figure S1: Confocal laser micrograph of *M. longiciliatum* (female from Oman). Blue areas are the least sclerotized and yellow the most strongly sclerotized (Photo A. P.); Figure S2: Phylogenetic tree of CO1 in *Megaphragma*, unpartitioned analysis in RAxML-NG; Figure S3. Phylogenetic tree of 28S in *Megaphragma*, unpartitioned analysis in RAxML-NG; Figure S4: a *Heliothrips haemorrhoidalis*, the host of *Megaphragma* sp., b eggs of *Heliothrips haemorrhoidalis* with exit hole of *Megaphragma* sp., c pupa of *Megaphragma* sp. in egg of *Heliothrips haemorrhoidalis* (Photo G. Viggiani).

## Appendix A

Details of the specimens used for the molecular analyses are provided in Table A1.

**Table A1:** Voucher specimens, their repository, and accession numbers for the 28S and CO1 sequences.

Nr	Voucher	Species	Country	Type status	Repository	28S		CO1	
						bp + gaps	Accession nr	bp	Accession nr
1	**COM1 1**	*M. nowickii*	D. R. Congo	Paratype	DACE	1095 (212 indels)	ON555486	295	ON557406
2	**COM1 2**	*M. nowickii*	D. R. Congo	Paratype	DACE	1095 (211 indels)	ON555494	652	ON557417
3	**COM1 3**	*M. nowickii*	D. R. Congo	Paratype	DACE	1095 (211 indels)	ON555502	652	ON557426
4	**COM1 4**	*M. nowickii*	D. R. Congo	Paratype	DACE	1000 (211 indels)	ON555503	652	ON557427
5	**COM1 5**	*M. nowickii*	D. R. Congo	Paratype	DACE	1092 (211 indels)	ON555504	652	ON557428
6	**COM1 6**	*M. nowickii*	D. R. Congo	Paratype	DACE	1085 (211 indels)	ON555505	652	ON557429
7	**COM1 7**	*M. nowickii*	D. R. Congo	Paratype	NHMUK	-	-	592	ON557430
8	**COM1 8**	*M. nowickii*	D. R. Congo	Paratype	NHMUK	1087 (212 indels)	ON555506	652	ON557431
9	**COM1 9**	*M. nowickii*	D. R. Congo	Paratype	NHMUK	1095 (211 indels)	ON555507	366	ON557432
10	**COM1 10**	*M. nowickii*	D. R. Congo	Paratype	NHMUK	1087 (211 indels)	ON555487	652	ON557407
11	**COM1 11**	*M. nowickii*	D. R. Congo	Paratype	NHMUK	414 (121 indels)	ON555488	652	ON557408
12	**COM1 12**	*M. nowickii*	D. R. Congo	Paratype	AICF	1093 (212 indels)	ON555489	652	ON557409
13	**COM1 13**	*M. nowickii*	D. R. Congo	Paratype	DACE	1048 (212 indels)	ON555490	652	ON557410
14	**COM1 14**	*M. nowickii*	D. R. Congo	Paratype	DACE	1049 (209 indels)	ON555491	652	ON557411
15	**COM1 15**	*M. nowickii*	D. R. Congo	Paratype	DACE	-	-	652	ON557412
16	**COM1 16**	*M. nowickii*	D. R. Congo	**Holotype**	NHMUK	1106 (211 indels)	ON555492	652	ON557413
17	**COM1 17**	*M. nowickii*	D. R. Congo	Paratype	DACE	-	-	652	ON557414
18	**COM1 18**	*M. nowickii*	D. R. Congo	Paratype	DACE	1106 (211 indels)	ON555493	652	ON557415
19	**COM1 19**	*M. nowickii*	D. R. Congo	Paratype	DACE	-	-	652	ON557416
20	**COM1 20**	*M. nowickii*	D. R. Congo	Paratype	DACE	1106 (212 indels)	ON555495	652	ON557418
21	**COM1 21**	*M. nowickii*	D. R. Congo	Paratype	AICF	1106 (212 indels)	ON555496	652	ON557419
22	**COM1 22**	*M. nowickii*	D. R. Congo	Paratype	IITA	1106 (208 indels)	ON555497	652	ON557420
23	**COM1 23**	*M. nowickii*	D. R. Congo	Paratype	IITA	1106 (209 indels)	ON555498	522	ON557421
24	**COM1 24**	*Megaphragma* sp.	D. R. Congo	NA	NHMUK	-	-	369	ON557422
25	**COM1 25**	*Megaphragma* sp.	D. R. Congo	NA	NHMUK	684 (245 indels)	ON555499	369	ON557423
26	**COM1 26**	*M. nowickii*	D. R. Congo	Paratype	IITA	1106 (211 indels)	ON555500	652	ON557424
27	**COM1 27**	*M. nowickii*	D. R. Congo	Paratype	IITA	1106 (211 indels)	ON555501	652	ON557425
28	**COM2 1**	*M. longiciliatum*	D. R. Congo	NA	AICF	918 (206 indels)	ON555508	652	ON557433
29	**COM2 2**	*Megaphragma* sp.	D. R. Congo	NA	NHMUK	1045 (239 indels)	ON555509	391	ON557434
30	**COM2 3**	*M. longiciliatum*	D. R. Congo	NA	NHMUK	896 (206 indels)	ON555510	-	-
31	**COM2 4**	*Megaphragma* sp.	D. R. Congo	NA	NHMUK	680 (124 indels)	ON555511	652	ON557435
32	**CRM1 4**	*M. hansoni*	Costa Rica	Paratype	NHMUK	534 (117 indels)	ON555513	-	-
33	**CRM1 16**	*M. hansoni*	Costa Rica	Paratype	NHMUK	1106 (255 indels)	ON555512	-	-
34	**CRM2 1**	*M. digitatum*	Costa Rica	**Holotype**	NHMUK	1062 (264 indels)	ON555514	-	-
35	**CRM2 2**	*M. antecessor*	Costa Rica	**Holotype**	NHMUK	647 (123 indels)	ON555518	366	ON557437
36	**CRM2 3**	*M. digitatum*	Costa Rica	Paratype	MZUCR	1106 (265 indels)	ON555519	652	ON557438
37	**CRM2 4**	*M. giraulti*	Costa Rica	**Holotype**	NHMUK	679 (158 indels)	ON555520	-	-
38	**CRM2 5**	*M. giraulti*	Costa Rica	Paratype	AICF	689 (159 indels)	ON555521	291	ON557439
39	**CRM2 6**	*M. digitatum*	Costa Rica	Paratype	MZUCR	1036 (264 indels)	ON555522	-	-
40	**CRM2 7**	*M. giraulti*	Costa Rica	Paratype	DACE	1048 (269 indels)	ON555523	291	ON557440
41	**CRM2 8**	*M. giraulti*	Costa Rica	Paratype	MZUCR	679 (158 indels)	ON555524	-	-
42	**CRM2 9**	*M. digitatum*	Costa Rica	Paratype	MZUCR	679 (151 indels)	ON555525	-	-
43	**CRM2 10**	*M. giraulti*	Costa Rica	Paratype	NHMUK	646 (158 indels)	ON555515	296	ON557436
44	**CRM2 11**	*M. giraulti*	Costa Rica	Paratype	MZUCR	490 (156 indels)	ON555516	-	-
45	**CRM2 12**	*M. digitatum*	Costa Rica	Paratype	AICF	646 (151 indels)	ON555517	-	-
46	**CRM3 4**	*M. hansoni*	Costa Rica	Paratype	NHMUK	938 (250 indels)	ON555530	-	-
47	**CRM3 17**	*M. hansoni*	Costa Rica	Paratype	NHMUK	1106 (255 indels)	ON555528	-	-
48	**CRM3 23**	*M. giraulti*	Costa Rica	Paratype	NHMUK	1043 (269 indels)	ON555529	-	-
49	**CRM3 40**	*M. hansoni*	Costa Rica	NA	lost	691 (374 indels)	ON555531	-	-
50	**CRM3 46**	*M. funiculatum*	Costa Rica	Paratype	AICF	1101 (233 indels)	ON555532	-	-
51	**CRM3 97**	*M. hansoni*	Costa Rica	Paratype	NHMUK	473 (137 indels)	ON555533	-	-
52	**CRM3 103**	*M. funiculatum*	Costa Rica	**Holotype**	NHMUK	761 (210 indels)	ON555526	-	-
53	**CRM3 104**	*M. hansoni*	Costa Rica	Paratype	NHMUK	1025 (255 indels)	ON555527	-	-
54	**DNA ECU1**	*M. digitatum*	Ecuador	Paratype	NHMUK	679 (152 indels)	ON555534	-	-
55	**DNA ECU4**	*M. digitatum*	Ecuador	Paratype	AICF	849 (321 indels)	ON555535	-	-
56	**DNA980**	*Megaphragma* sp.	D. R. Congo	NA	NHMUK	1106 (261 indels)	ON555584	-	-
57	**DNA1111**	*M. longiciliatum*	USA	NA	NHMUK	1013 (225 indels)	ON555536	-	-
58	**DNA1112**	*M. longiciliatum*	USA	NA	NHMUK	1030 (225 indels)	ON555537	-	-
59	**DNA1113**	*M. longiciliatum*	USA	NA	NHMUK	912 (266 indels)	ON555538	-	-
60	**DNA1114**	*M. mymaripenne*	USA	NA	NHMUK	1056 (228 indels)	ON555539	-	-
61	**DNA1116**	*M. nowickii*	Uganda	Paratype	NHMUK	1085 (211 indels)	ON555540	-	-
62	**DNA1117**	*M. nowickii*	Uganda	Paratype	NHMUK	1085 (211 indels)	ON555541	-	-
63	**DNA1118**	*M. nowickii*	Uganda	Paratype	NHMUK	1085 (212 indels)	ON555542	-	-
64	**DNA1119**	*Megaphragma* sp.	Uganda	NA	NHMUK	1031 (238 indels)	ON555543	-	-
65	**DNA1120**	*M. nowickii*	Uganda	Paratype	NHMUK	473 (93 indels)	ON555544	-	-
66	**DNA1121**	*M. nowickii*	Uganda	Paratype	NHMUK	806 (181 indels)	ON555545	-	-
67	**DNA1122**	*M. nowickii*	Uganda	Paratype	NHMUK	1052 (211 indels)	ON555546	-	-
68	**DNA1123**	*M. nowickii*	Uganda	Paratype	NHMUK	1085 (211 indels)	ON555547	-	-
69	**DNA1124**	*M. nowickii*	Uganda	Paratype	NHMUK	1085 (212 indels)	ON555548	-	-
70	**DNA1132**	*M. nowickii*	Uganda	Paratype	NHMUK	1085 (209 indels)	ON555549	-	-
71	**DNA1147**	*M. longiciliatum*	Indonesia	NA	NHMUK	1097 (202 indels)	ON555550	-	-
72	**DNA1612**	*M. noyesi*	UK	NA	lost	1067 (213 indels)	ON555551	-	-
73	**DNA1613**	*M. noyesi*	UK	Paratype	NHMUK	1106 (213 indels)	ON555552	607	ON557441
74	**DNA1614**	*M. noyesi*	UK	NA	lost	1106 (213 indels)	ON555553	-	-
75	**DNA1615**	*M. noyesi*	UK	Paratype	NHMUK	908 (189 indels)	ON555554	607	ON557442
76	**DNA1616**	*M. noyesi*	UK	Paratype	NHMUK	1106 (213 indels)	ON555555	607	ON557443
77	**DNA1617**	*M. noyesi*	UK	Paratype	UCRC	1106 (213 indels)	ON555556	-	-
78	**DNA1618**	*M. noyesi*	UK	NA	lost	1065 (213 indels)	ON555557	-	-
79	**DNA1619**	*M. noyesi*	UK	Paratype	UCRC	1106 (213 indels)	ON555558	607	ON557444
80	**DNA1626**	*Megaphragma* sp.	Brunei	NA	NHMUK	1067 (271 indels)	ON555559	-	-
81	**DNA1628**	*Megaphragma* sp.	Singapore	NA	NHMUK	1096 (266 indels)	ON555560	394	ON557445
82	**DNA1630**	*Megaphragma* sp.	Singapore	NA	NHMUK	-	-	394	ON557446
83	**DNA1632**	*Megaphragma* sp.	Singapore	NA	NHMUK	-	-	394	ON557447
84	**DNA1638**	*Megaphragma* sp.	Brunei	NA	NHMUK	1071 (265 indels)	ON555561	-	-
85	**DNA1640**	*Megaphragma* sp.	Singapore	NA	NHMUK	1046 (156 indels)	ON555562	394	ON557448
86	**DNA1641**	*Megaphragma* sp.	Singapore	NA	NHMUK	1106 (269 indels)	ON555563	394	ON557449
87	**DNA1642**	*Megaphragma* sp.	Brunei	NA	NHMUK	1106 (269 indels)	ON555564	394	ON557450
88	**DNA1643**	*Megaphragma* sp.	Brunei	NA	NHMUK	975 (264 indels)	ON555565	394	ON557451
89	**DNA1644**	*Megaphragma* sp.	Brunei	NA	NHMUK	1106 (271 indels)	ON555566	394	ON557452
90	**DNA1645**	*Megaphragma* sp.	Brunei	NA	NHMUK	1042 (268 indels)	ON555567	394	ON557453
91	**DNA1650**	*Megaphragma* sp.	Brunei	NA	NHMUK	1072 (270 indels)	ON555568	-	-
92	**DNA1651**	*Megaphragma* sp.	Brunei	NA	NHMUK	1048 (222 indels)	ON555569	394	ON557454
93	**DNA1652**	*Megaphragma* sp.	Brunei	NA	NHMUK	1106 (267 indels)	ON555570	394	ON557455
94	**DNA1655**	*Megaphragma* sp.	Brunei	NA	NHMUK	1000 (270 indels)	ON555571	394	ON557456
95	**DNA1656**	*M. liui*	Brunei	**Holotype**	UCRC	900 (556 indels)	ON555572	370	ON557457
96	**DNA1659**	*Megaphragma* sp.	Brunei	NA	NHMUK	1038 (270 indels)	ON555573	-	-
97	**DNA1661**	*Megaphragma* sp.	Brunei	NA	NHMUK	1106 (266 indels)	ON555574	-	-
98	**DNA1665**	*Megaphragma* sp.	Singapore	NA	NHMUK	1106 (266 indels)	ON555575	394	ON557458
99	**DNA1668**	*Megaphragma* sp.	Brunei	NA	NHMUK	1106 (267 indels)	ON555576	394	ON557459
100	**DNA1674**	*Megaphragma* sp.	Brunei	NA	NHMUK	1106 (265 indels)	ON555577	-	-
101	**DNA1678**	*Megaphragma* sp.	Singapore	NA	NHMUK	1095 (266 indels)	ON555578	394	ON557460
102	**DNA1679**	*Megaphragma* sp.	Brunei	NA	NHMUK	1106 (218 indels)	ON555579	-	-
103	**DNA1680**	*M. momookherjeeae*	Costa Rica	**Holotype**	NHMUK	-	-	394	ON557461
104	**DNA1681**	*M. digitatum*	Costa Rica	Paratype	NHMUK	1106 (265 indels)	ON555580	367	ON557462
105	**DNA1682**	*Megaphragma* sp.	Costa Rica	NA	NHMUK	932 (257 indels)	ON555581	-	-
106	**DNA1683**	*M. giraulti*	Costa Rica	Paratype	NHMUK	1055 (270 indels)	ON555582	394	ON557463
107	**DNA1686**	*Megaphragma* sp.	Vietnam	NA	NHMUK	-	-	652	ON557464
108	**DNA1687**	*M. rivelloi*	Vietnam	Paratype	AICF	1106 (259 indels)	ON555583	-	-
109	**DNAMO3**	*M. longiciliatum*	Oman	NA	NHMO	420 (102 indels)	ON555588	-	-
110	**DNAMO13**	*M. longiciliatum*	Oman	NA	NHMO	1106 (224 indels)	ON555585	-	-
111	**DNAMO20**	*M. longiciliatum*	Oman	NA	NHMO	1106 (224 indels)	ON555586	-	-
112	**DNAMO22**	*M. longiciliatum*	Oman	NA	NHMO	1106 (224 indels)	ON555587	-	-
113	**FRM2**	*M. longiciliatum*	France	NA	NHMUK	1106 (213 indels)	ON555589	652	ON557465
114	**FRM3**	*M. longiciliatum*	France	NA	NHMUK	1106 (213 indels)	ON555590	652	ON557466
115	**FRM4**	*M. longiciliatum*	France	NA	AICF	1106 (213 indels)	ON555591	652	ON557467
116	**FRM5**	*M. longiciliatum*	France	NA	NHMUK	1106 (213 indels)	ON555592	652	ON557468
117	**FRM6**	*M. longiciliatum*	France	NA	NHMUK	1106 (213 indels)	ON555593	652	ON557469
118	**HUM1**	*M. longiciliatum*	Hungary	NA	NHMUK	679 (122 indels)	ON555594	652	ON557470
119	**HUM2**	*M. noyesi*	Hungary	Paratype	NHMUK	1060 (213 indels)	ON555599	650	ON557476
120	**HUM3**	*M. noyesi*	Hungary	NA	lost	1061 (213 indels)	ON555600	652	ON557477
121	**HUM4**	*Megaphragma* sp.	Hungary	NA	NHMUK	1010 (216 indels)	ON555601	652	ON557478
122	**HUM5**	*M. noyesi*	Hungary	Paratype	AICF	-	-	652	ON557479
123	**HUM6**	*M. noyesi*	Hungary	Paratype	AICF	1060 (213 indels)	ON555602	614	ON557480
124	**HUM8**	*M. noyesi*	Hungary	Paratype	NHMUK	641 (152 indels)	ON555603	652	ON557481
125	**HUM9**	*M. noyesi*	Hungary	Paratype	NHMUK	-	-	296	ON557482
126	**HUM10**	*M. noyesi*	Hungary	Paratype	NHMUK	679 (125 indels)	ON555595	652	ON557471
127	**HUM11**	*M. noyesi*	Hungary	Paratype	NHMUK	649 (125 indels)	ON555596	603	ON557472
128	**HUM12**	*M. noyesi*	Hungary	Paratype	NHMUK	1040 (213 indels)	ON555597	295	ON557473
129	**HUM13**	*M. noyesi*	Hungary	Paratype	NHMUK	-	-	574	ON557474
130	**HUM14**	*M. noyesi*	Hungary	Paratype	NHMUK	763 (192 indels)	ON555598	643	ON557475
131	**ITM1**	*M. viggianii*	Italy	**Holotype**	DACE	1106 (219 indels)	ON555604	652	ON557483
132	**ITM2**	*M. viggianii*	Italy	Paratype	NHMUK	1106 (219 indels)	ON555610	652	ON557488
133	**ITM3**	*M. viggianii*	Italy	Paratype	NHMUK	934 (217 indels)	ON555611	631	ON557489
134	**ITM4**	*M. viggianii*	Italy	Paratype	NHMUK	1106 (219 indels)	ON555612	652	ON557490
135	**ITM5**	*M. viggianii*	Italy	Paratype	AICF	1050 (219 indels)	ON555613	652	ON557491
136	**ITM6**	*M. viggianii*	Italy	Paratype	AICF	1106 (219 indels)	ON555614	652	ON557492
137	**ITM7**	*M. viggianii*	Italy	Paratype	NHMUK	1047 (219 indels)	ON555615	296	ON557493
138	**ITM8**	*M. polilovi*	Italy	Paratype	DACE	1080 (208 indels)	ON555616	641	ON557494
139	**ITM9**	*M. polilovi*	Italy	**Holotype**	DACE	1088 (208 indels)	ON555617	641	ON557495
140	**ITM10**	*M. longiciliatum*	Italy	NA	NHMUK	1081 (213 indels)	ON555605	-	-
141	**ITM11**	*M. polilovi*	Italy	Paratype	AICF	1080 (208 indels)	ON555606	641	ON557484
142	**ITM12**	*M. polilovi*	Italy	Paratype	NHMUK	1081 (208 indels)	ON555607	652	ON557485
143	**ITM13**	*M. viggianii*	Italy	Paratype	NHMUK	1081 (219 indels)	ON555608	641	ON557486
144	**ITM14**	*M. polilovi*	Italy	Paratype	NHMUK	1080 (208 indels)	ON555609	641	ON557487
145	**MXM1**	*M. striatum*	Mexico	NA	NHMUK	995 (297 indels)	ON555618	652	ON557496
146	**MXM2**	*M. breviclavum*	Mexico	**Holotype**	NHMUK	1010 (298 indels)	ON555619	652	ON557497
147	**MXM3**	*M. breviclavum*	Mexico	Paratype	NHMUK	878 (514 indels)	ON555620	652	ON557498
148	**MXM4**	*M. breviclavum*	Mexico	Paratype	NHMUK	1000 (298 indels)	ON555621	575	ON557499
149	**SAM1**	*Megaphragma* sp.	Malaysia	NA	NHMUK	1106 (269 indels)	ON555622	296	ON557500
150	**SAM2**	*Megaphragma* sp.	Malaysia	NA	NHMUK	1065 (248 indels)	ON555626	356	ON557503
151	**SAM3**	*M. vanlentereni*	Malaysia	**Holotype**	NHMUK	1017 (263 indels)	ON555627	652	ON557504
152	**SAM4**	*M. chienleei*	Malaysia	Paratype	NHMUK	1106 (268 indels)	ON555628	-	-
153	**SAM5**	*M. chienleei*	Malaysia	Paratype	NHMUK	1106 (268 indels)	ON555629	652	ON557505
154	**SAM6**	*M. chienleei*	Malaysia	Paratype	AICF	1106 (268 indels)	ON555630	652	ON557506
155	**SAM7**	*M. chienleei*	Malaysia	Paratype	NHMUK	1106 (268 indels)	ON555631	652	ON557507
156	**SAM8**	*M. chienleei*	Malaysia	Paratype	NHMUK	1106 (268 indels)	ON555632	652	ON557508
157	**SAM9**	*Megaphragma* sp.	Malaysia	NA	NHMUK	1106 (271 indels)	ON555633	652	ON557509
158	**SAM10**	*Megaphragma* sp.	Malaysia	NA	NHMUK	1080 (225 indels)	ON555623	641	ON557501
159	**SAM11**	*M. cockerilli*	Malaysia	**Holotype**	AICF	1073 (222 indels)	ON555624	460	ON557502
160	**SAM12**	*M. chienleei*	Malaysia	**Holotype**	AICF	1074 (268 indels)	ON555625	-	-
161	**SRM1**	*M. longiciliatum*	Malaysia	NA	NHMUK	513 (287 indels)	ON555634	652	ON557510
162	**SRM2**	*Megaphragma* sp.	Malaysia	NA	NHMUK	1044 (248 indels)	ON555635	624	ON557511
163	**SRM3**	*Megaphragma* sp.	Malaysia	NA	NHMUK	679 (124 indels)	ON555636	601	ON557512
164	**UKM8**	*M. noyesi*	UK	Paratype	NHMUK	647 (125 indels)	ON555642	-	-
165	**UKM9**	*M. noyesi*	UK	Paratype	lost	1106 (213 indels)	ON555643	652	ON557518
166	**UKM10**	*M. noyesi*	UK	Paratype	AICF	1106 (213 indels)	ON555637	652	ON557513
167	**UKM11**	*M. noyesi*	UK	Paratype	NHMUK	1106 (213 indels)	ON555638	652	ON557514
168	**UKM12**	*M. noyesi*	UK	Paratype	DACE	1106 (213 indels)	ON555639	652	ON557515
169	**UKM13**	*M. noyesi*	UK	Paratype	DACE	1106 (213 indels)	ON555640	652	ON557516
170	**UKM14**	*M. noyesi*	UK	**Holotype**	NHMUK	1047 (213 indels)	ON555641	652	ON557517
171	**-**	“*M. amalphitanum*”	**GenBank**	NA	-	-	-	652	KT373787
172	**D1224**	*Megaphragma* sp.	**GenBank**	NA	-	1048 (230 indels)	AY623543	-	-
173	**D1229**	*Megaphragma* sp.	**GenBank**	NA	-	1106 (260 indels)	AY623544	-	-
174	**D1243**	*Megaphragma* sp.	**GenBank**	NA	-	1106 (240 indels)	AY623545	-	-
175	**O19**	*Oligosita* sp.	**GenBank**	NA	-	659 (195 indels)	MG785509	603	MG904913
176	**D1219**	*Epoligosita* sp.	**GenBank**	NA	-	1106 (301 indels)	AY623546	-	-
177	**D0760**	*Oligosita sanguinea*	**GenBank**	NA	-	1106 (291 indels)	AY623551	-	-
178	**D0886**	*Probrachista* sp.	**GenBank**	NA	-	1106 (288 indels)	AY623553	-	-
