# Supplementary material for: Revision of the World Species of Megaphragma Timberlake (Hymenoptera: Trichogrammatidae)
Source: Insects. 2022 Jun 20;13(6):561. doi: 10.3390/insects13060561 (PMC9225605; doi:10.3390/insects13060561)
Supplement: Supplementary file 1 [file insects-13-00561-s001.zip › insects-1708174-supplementary.pdf]

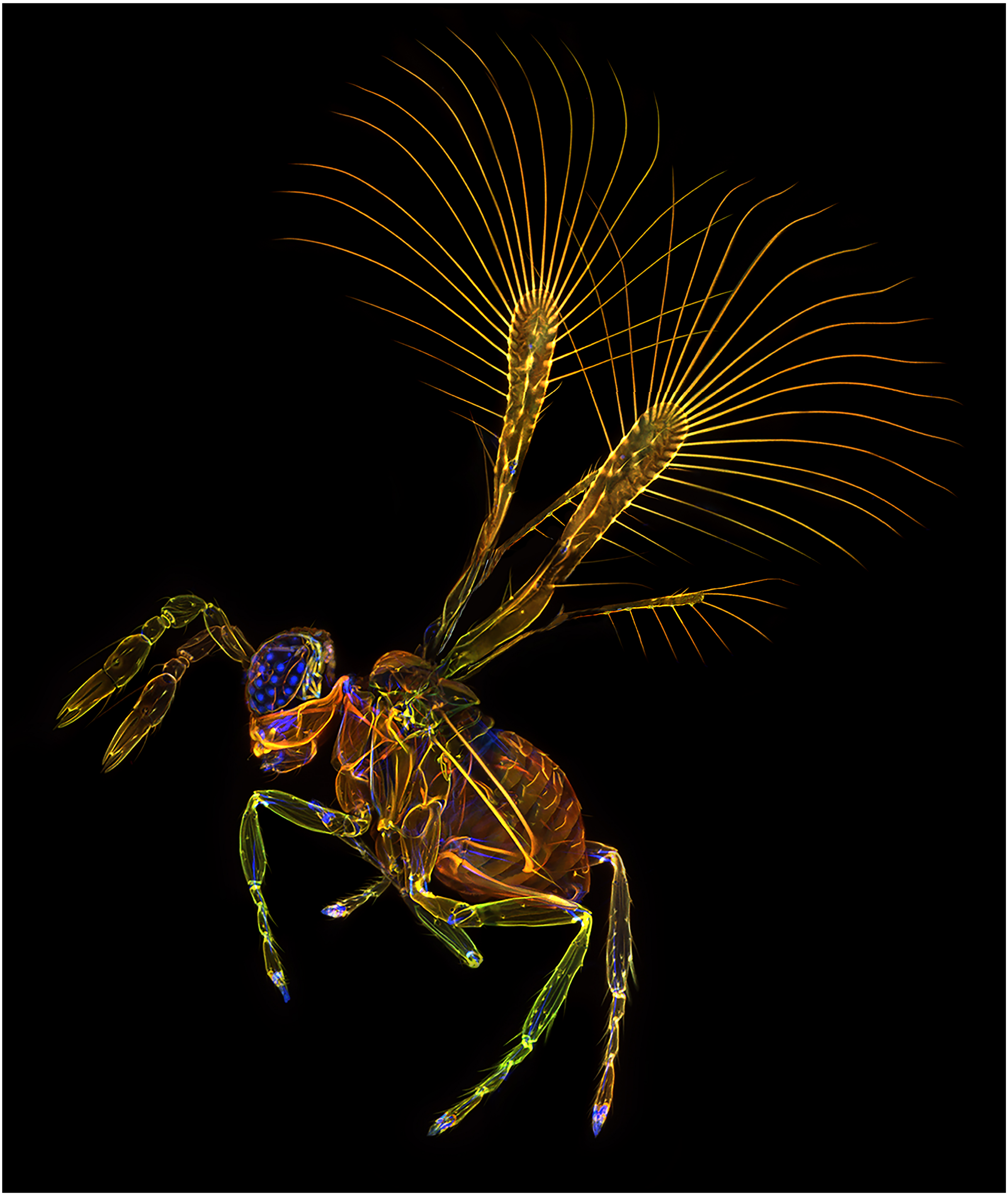

Figure S1. Confocal laser micrograph of *Megaphragma longiciliatum* (female from Oman). Blue areas are the least sclerotised and yellow the most strongly sclerotized (Photo A. Polaszek).

Figure S2. Phylogenetic tree of CO1 in ***Megaphragma***, unpartitioned analysis in RAxML-NG, GTR model.

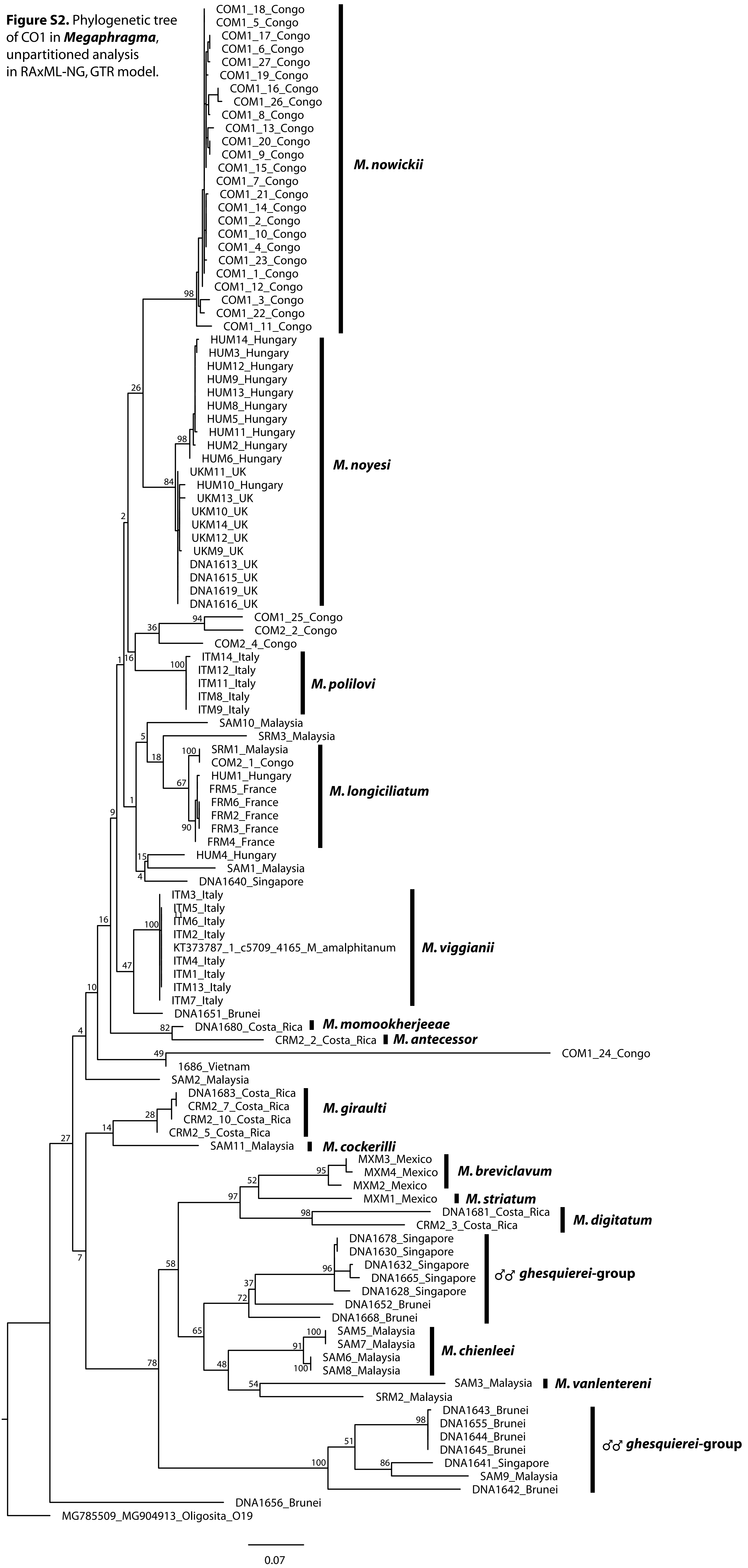

Figure S3. Phylogenetic tree of 28S in *Megaphragma*, unpartitioned analysis in RAxML-NG, K2P model.

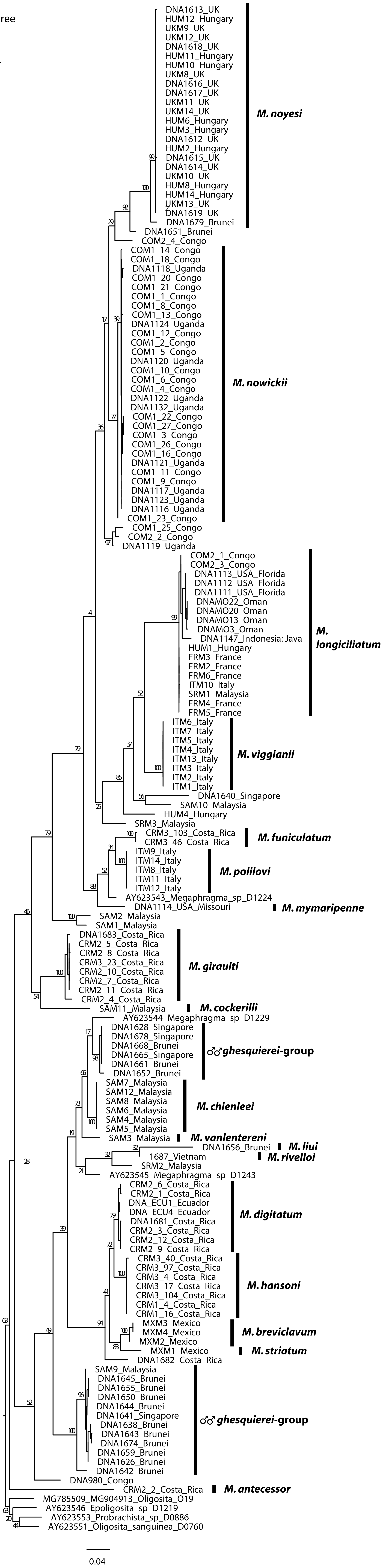

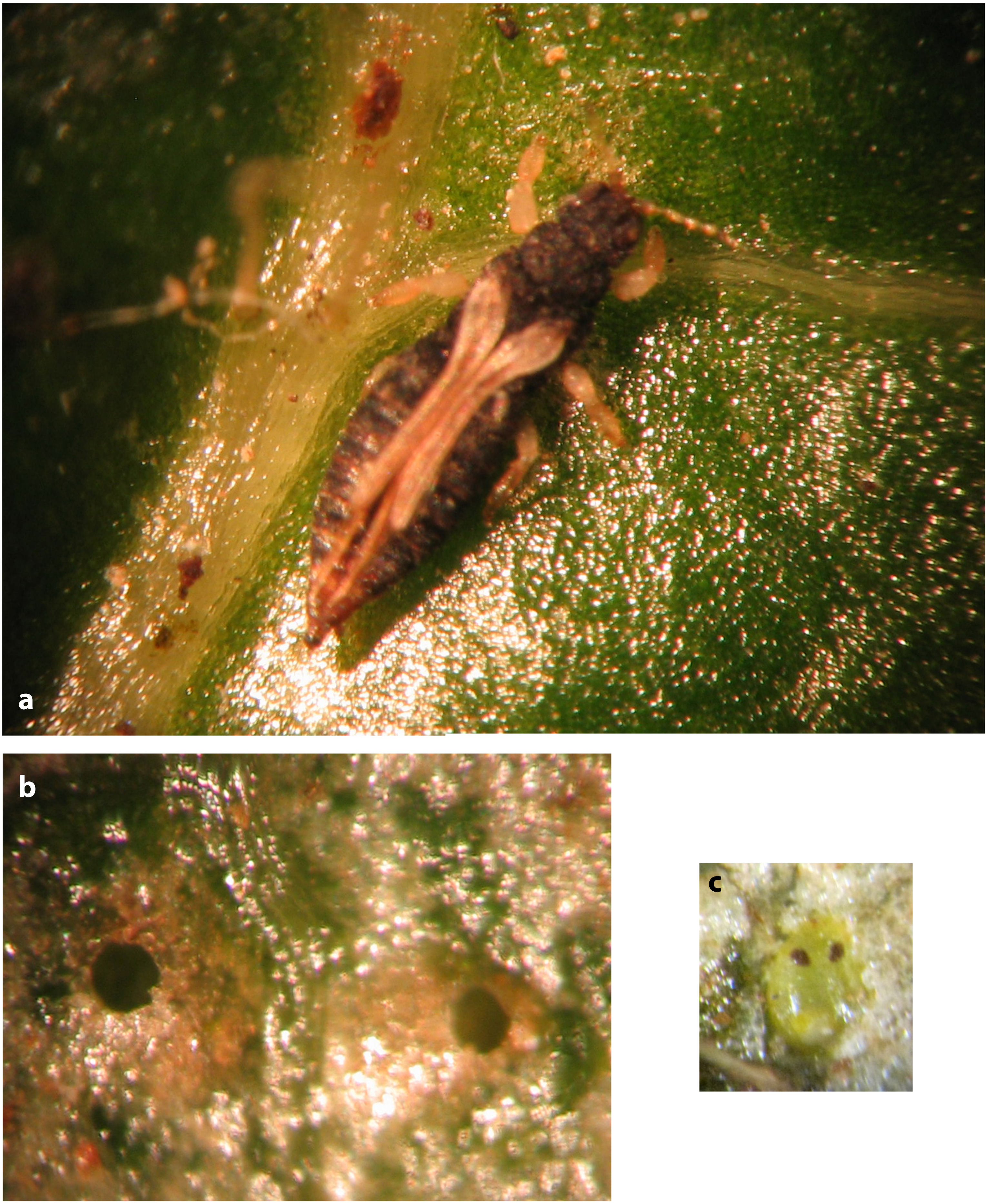

Figure S4: a *Heliothrips haemorrhoidalis*, the host of *Megaphragma* sp.,
b eggs of *Heliothrips haemorrhoidalis* with exit hole of *Megaphragma* sp.,
c pupa of *Megaphragma* sp. in egg of *Heliothrips haemorrhoidalis* (Photo G. Viggiani).

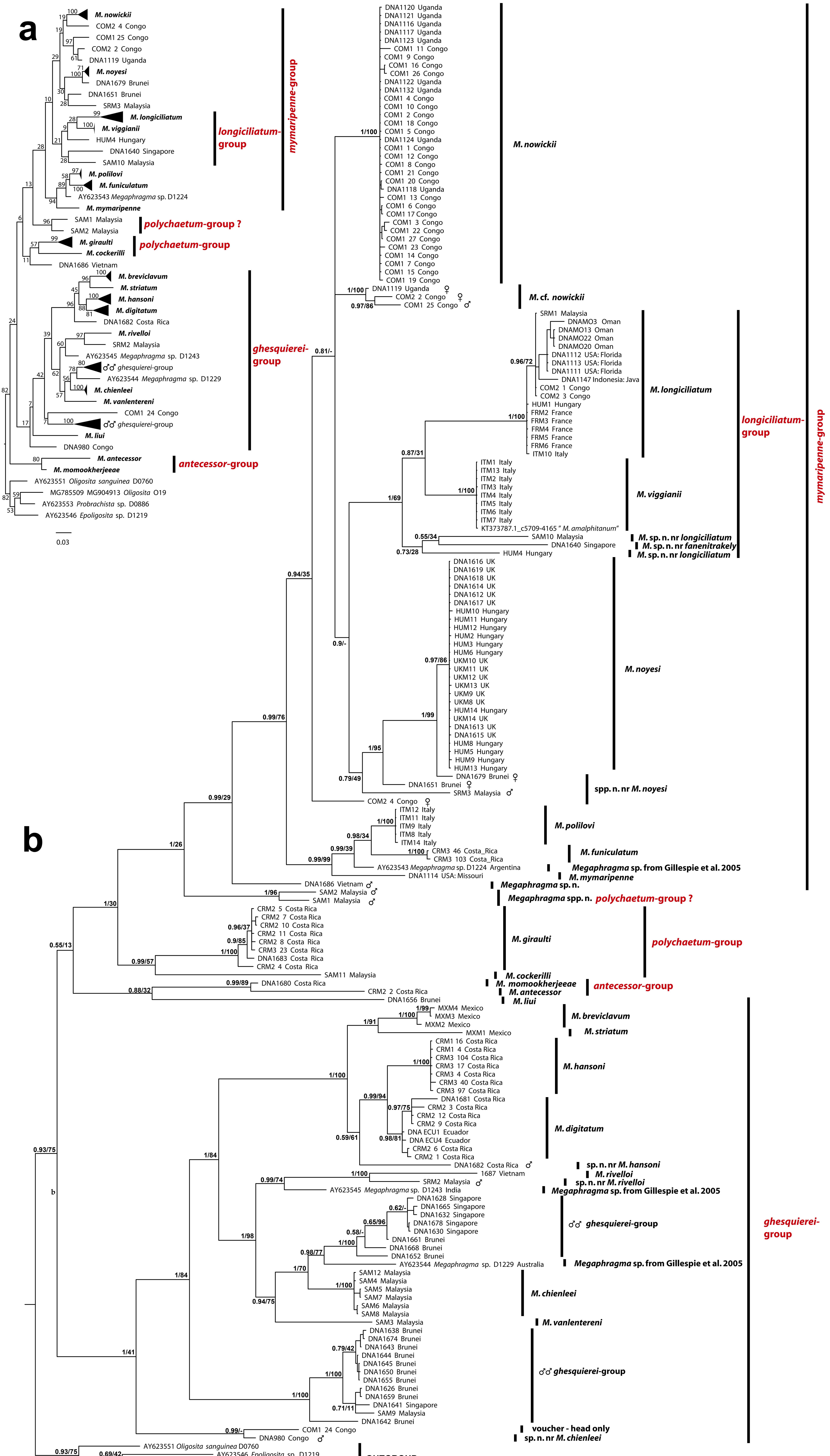

Figure 2/S5. Combined CO1 and 28S sequences analysis of 174 *Megaphragma* specimens (170 from this study, 4 from GenBank) with species delineated and species-groups indicated.
a unpartitioned ML analysis (bootstrap values indicated at nodes);
b partitioned BI analysis (both posterior probabilities and bootstrap values indicated at nodes).
